# Supplementary material for: New Diterpenes and Diterpene Glycosides with Antibacterial Activity from Soft Coral Lemnalia bournei
Source: Mar Drugs. 2024 Mar 29;22(4):157. doi: 10.3390/md22040157 (PMC11051049; doi:10.3390/md22040157)
Supplement: Supplementary file 1 [file marinedrugs-22-00157-s001.zip › marinedrugs-2937748-supplementary.pdf]

Supporting Information

New Diterpenes and Diterpene Glycosides with Antibacterial Activity from Soft Coral *Lemnalia bournei*

Xiao Han[3], Huiting Wang[1], Bing Li[1], Xiaoyi Chen[3], Te Li[3], Xia Yan[1], Wenhan Lin[4], Han Ouyang[2,]* and Shan He[1,4]*

[1] *Li Dak Sum Yip Yio Chin Kenneth Li Marine Biopharmaceutical Research Center, Health Science Center, Ningbo University, Ningbo 315211, Zhejiang, China; 212746480@qq.com (H.W.); 212746480@qq.com (B.L.); yanxia@nbu.edu.cn (X.Y.); heshan@nbu.edu.cn (S.H.)*

[2] *Institute of Drug Discovery Technology, Ningbo University, Ningbo, Zhejiang 315211, China; ouyanghan@nbu.edu.cn (H.O.)*

[3] *Department of Marine Pharmacy, College of Food Science and Engineering, Ningbo University, Ningbo 315800, Zhejiang, China; 1209420905@qq.com (X.H.); chennimo@163.com (X.C.); 1209420905@qq.com (Y.W.); telinbu@163.com (T.L.)*

[4] *Ningbo Institute of Marine Medicine, Peking University, Ningbo, Zhejiang 315800, China; whlin@bjmu.edu.cn (W.L.)*

**Correspondence: ouyanghan@nbu.edu.cn (H.O.); heshan@nbu.edu.cn (S.H.)*

Table of Contents

Fig. S1 ^{1}H NMR spectrum (600 MHz) of compound **1** in $CDCl_3$. 4
Fig. S2 ^{13}C NMR spectrum (150 MHz) of compound **1** in $CDCl_3$. 4
Fig. S3 DEPT spectrum (150 MHz) of compound **1** in $CDCl_3$. 5
Fig. S4 ^{1}H-^{1}H COSY spectrum (600 MHz) of compound **1** in $CDCl_3$. 5
Fig. S5 HSQC spectrum (600 MHz) of compound **1** in $CDCl_3$. 6
Fig. S6 HMBC spectrum (600 MHz) of compound **1** in $CDCl_3$. 6
Fig. S7 ^{1}D -NOE (δ_H0.83) spectrum (600 MHz) of compound **1** in $CDCl_3$. 7
Fig. S8 ^{1}D -NOE (δ_H1.74) spectrum (600 MHz) of compound **1** in $CDCl_3$. 7
Fig. S9 ^{1}D -NOE (δ_H1.20) spectrum (600 MHz) of compound **1** in $CDCl_3$. 8
Fig. S10 ^{1}D -NOE (δ_H2.23) spectrum (600 MHz) of compound **1** in $CDCl_3$. 8
Fig. S11 HR-ESIMS spectrum of compound **1**. 9
Fig. S12 Infrared spectrum of compound **1**. 9
Fig. S13 ^{1}H NMR spectrum (600 MHz) of compound **2** in $CDCl_3$. 10
Fig. S14 ^{13}C NMR spectrum (150 MHz) of compound **2** in $CDCl_3$. 10
Fig. S15 DEPT spectrum (150 MHz) of compound **2** in $CDCl_3$. 11
Fig. S16 ^{1}H-^{1}H COSY spectrum (600 MHz) of compound **2** in $CDCl_3$. 11
Fig. S17 HSQC spectrum (600 MHz) of compound **2** in $CDCl_3$. 12
Fig. S18 HMBC spectrum (600 MHz) of compound **2** in $CDCl_3$. 12
Fig. S19 ^{1}D-NOE (δ_H2.23) spectrum (600 MHz) of compound **2** in $CDCl_3$. 13
Fig. S20 ^{1}D -NOE (δ_H0.85) spectrum (600 MHz) of compound **2** in $CDCl_3$. 13
Fig. S21 ^{1}D -NOE (δ_H1.80) spectrum (600 MHz) of compound **2** in $CDCl_3$. 14
Fig. S22 ^{1}D -NOE (δ_H2.05) spectrum (600 MHz) of compound **2** in $CDCl_3$. 14
Fig. S23 ^{1}D -NOE (δ_H1.30) spectrum (600 MHz) of compound **2** in $CDCl_3$. 15
Fig. S24 HR-ESIMS spectrum of compound **2**. 15
Fig. S25 Infrared spectrum of compound **2**. 16
Fig. S26 ^{1}H NMR spectrum (600 MHz) of compound **3** in $CDCl_3$. 16
Fig. S27 ^{13}C NMR spectrum (150 MHz) of compound **3** in $CDCl_3$. 17
Fig. S28 DEPT spectrum (150 MHz) of compound **3** in $CDCl_3$. 17
Fig. S29 ^{1}H-^{1}H COSY spectrum (600 MHz) of compound **3** in $CDCl_3$. 18
Fig. S30 HSQC spectrum (600 MHz) of compound **3** in $CDCl_3$. 18
Fig. S31 HMBC spectrum (600 MHz) of compound **3** in $CDCl_3$. 19
Fig. S32 ^{1}D -NOE (δ_H1.29) spectrum (600 MHz) of compound **3** in $CDCl_3$. 19
Fig. S33 ^{1}D -NOE (δ_H1.72) spectrum (600 MHz) of compound **3** in $CDCl_3$. 20
Fig. S34 ^{1}D -NOE (δ_H0.79) spectrum (600 MHz) of compound **3** in $CDCl_3$. 20
Fig. S35 ^{1}D -NOE (δ_H2.03) spectrum (600 MHz) of compound **3** in $CDCl_3$. 21
Fig. S36 HR-ESIMS spectrum of compound **3**. 21
Fig. S37 Infrared spectrum of compound **3**. 22
Fig. S38 ^{1}H NMR spectrum (600 MHz) of compound **4** in $CDCl_3$. 22
Fig. S39 ^{13}C NMR spectrum (150 MHz) of compound **4** in $CDCl_3$. 23
Fig. S40 DEPT spectrum (150 MHz) of compound **4** in $CDCl_3$. 23
Fig. S41 ^{1}H-^{1}H COSY spectrum (600 MHz) of compound **4** in $CDCl_3$ 24
Fig. S42 HSQC spectrum (600 MHz) of compound **4** in $CDCl_3$. 24

Fig. S43 HMBC spectrum (600 MHz) of compound **4** in $CDCl_3$. 25
Fig. S44 ^{1}D -NOE (δ_H6.76) spectrum (600 MHz) of compound **4** in $CDCl_3$. 25
Fig. S45 ^{1}D -NOE (δ_H1.83) spectrum (600 MHz) of compound **4** in $CDCl_3$. 26
Fig. S46 ^{1}D -NOE (δ_H1.14) spectrum (600 MHz) of compound **4** in $CDCl_3$. 26
Fig. S47 ^{1}D -NOE (δ_H0.79) spectrum (600 MHz) of compound **4** in $CDCl_3$. 27
Fig. S48 ^{1}D -NOE (δ_H1.76) spectrum (600 MHz) of compound **4** in $CDCl_3$. 27
Fig. S49 ^{1}D -NOE (δ_H1.10) spectrum (600 MHz) of compound **4** in $CDCl_3$. 28
Fig. S50 HR-ESIMS spectrum of compound **4**. 28
Fig. S51 Infrared spectrum of compound **4**. 29
Fig. S53 ^{13}C NMR spectrum (150 MHz) of compound **5** in $CDCl_3$. 30
Fig. S54 DEPT spectrum (150 MHz) of compound **5** in $CDCl_3$. 30
Fig. S55 ^{1}H-^{1}H COSY spectrum (600 MHz) of compound **5** in $CDCl_3$. 31
Fig. S56 HSQC spectrum (600 MHz) of compound **5** in $CDCl_3$. 31
Fig. S57 HMBC spectrum (600 MHz) of compound **5** in $CDCl_3$. 32
Fig. S59 ^{1}D -NOE (δ_H2.04) spectrum (600 MHz) of compound **5** in $CDCl_3$. 33
Fig. S60 ^{1}D -NOE (δ_H0.83) spectrum (600 MHz) of compound **5** in $CDCl_3$. 33
Fig. S61 ^{1}D -NOE (δ_H6.73) spectrum (600 MHz) of compound **5** in $CDCl_3$. 34
Fig. S62 HR-ESIMS spectrum of compound **5**. 34
Fig. S63 Infrared spectrum of compound **5**. 35
Fig. S64 ^{1}H NMR spectrum (600 MHz) of compound **6** in $CDCl_3$. 35
Fig. S65 ^{13}C NMR spectrum (150 MHz) of compound **6** in $CDCl_3$. 36
Fig. S66 DEPT spectrum (150 MHz) of compound **6** in $CDCl_3$. 36
Fig. S68 HSQC spectrum (600 MHz) of compound **6** in $CDCl_3$. 37
Fig. S69 HMBC spectrum (600 MHz) of compound **6** in $CDCl_3$. 38
Fig. S70 ^{1}D -NOE (δ_H2.03) spectrum (600 MHz) of compound **6** in $CDCl_3$. 38
Fig. S71 ^{1}D -NOE (δ_H0.81) spectrum (600 MHz) of compound **6** in $CDCl_3$. 39
Fig. S72 ^{1}D -NOE (δ_H4.60) spectrum (600 MHz) of compound **6** in $CDCl_3$. 39
Fig. S73 ^{1}D -NOE (δ_H4.41) spectrum (600 MHz) of compound **6** in $CDCl_3$. 40
Fig. S74 ^{1}D -NOE (δ_H4.88) spectrum (600 MHz) of compound **6** in $CDCl_3$. 40
Fig. S75 HR-ESIMS spectrum of compound **6**. 41
Fig. S76 Infrared spectrum of compound **6**. 41
Fig. S77 ^{1}H NMR spectrum (600 MHz) of compound **7** in $CDCl_3$. 42
Fig. S78 ^{13}C NMR spectrum (150 MHz) of compound **7** in $CDCl_3$. 42
Fig. S79 DEPT spectrum (150 MHz) of compound **7** in $CDCl_3$. 43
Fig. S80 ^{1}H-^{1}H COSY spectrum (600 MHz) of compound **7** in $CDCl_3$. 43
Fig. S81 HSQC spectrum (600 MHz) of compound **7** in $CDCl_3$. 44
Fig. S82 HMBC spectrum (600 MHz) of compound **7** in $CDCl_3$. 44
Fig. S83 ^{1}D -NOE (δ_H0.80) spectrum (600 MHz) of compound **7** in $CDCl_3$. 45
Fig. S84 ^{1}D -NOE (δ_H1.49) spectrum (600 MHz) of compound **7** in $CDCl_3$. 45
Fig. S85 HR-ESIMS spectrum of compound **7**. 46
Fig. S86 Infrared spectrum of compound **7**. 46

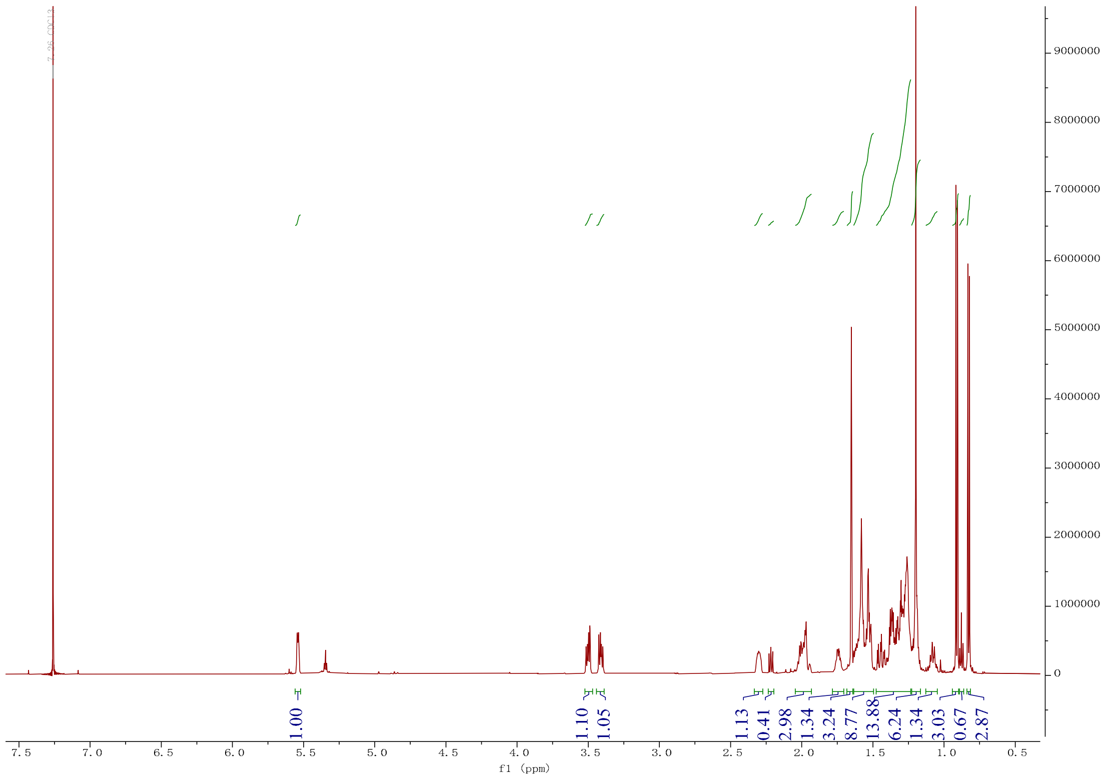

Fig. S1 ^{1}H NMR spectrum (600 MHz) of compound **1** in $CDCl_3$.

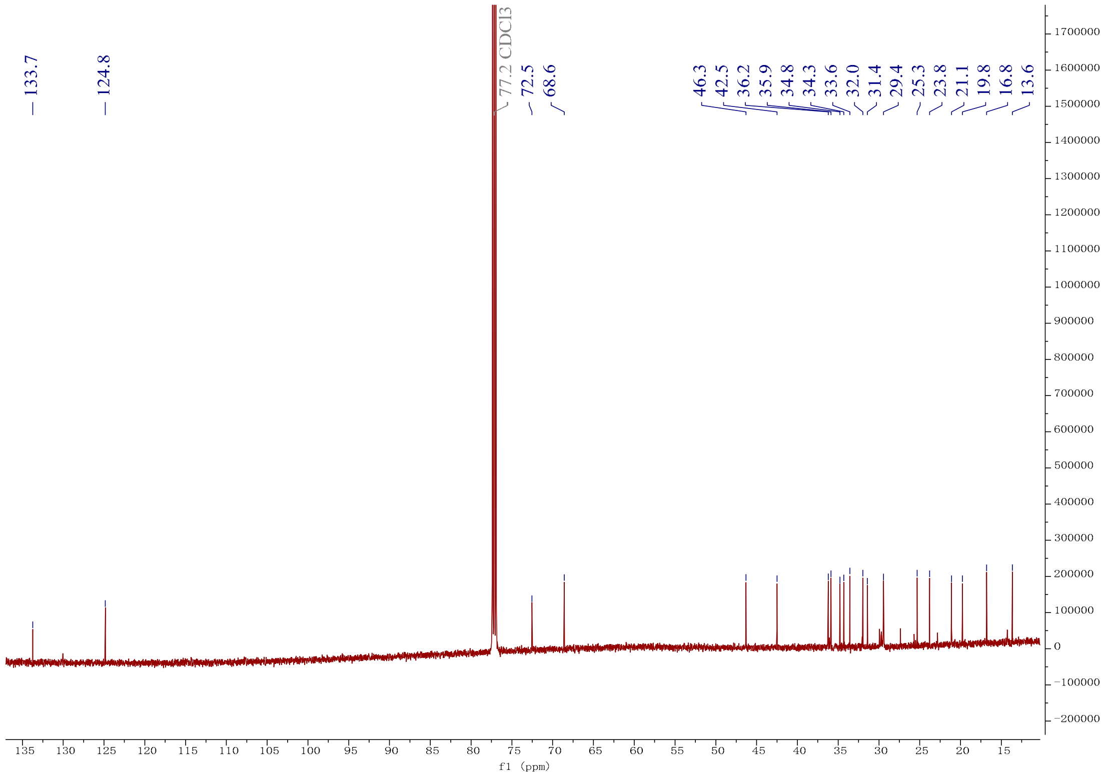

Fig. S2 ^{13}C NMR spectrum (150 MHz) of compound **1** in $CDCl_3$.

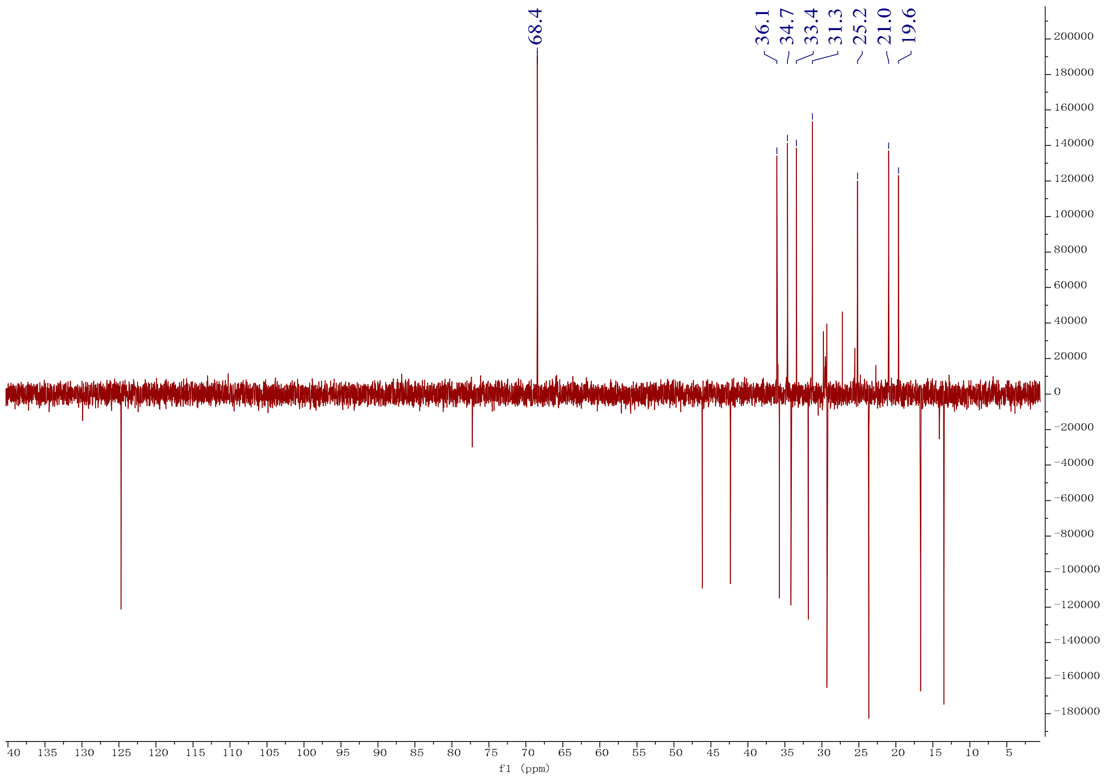

Fig. S3 DEPT spectrum (150 MHz) of compound **1** in $CDCl_3$.

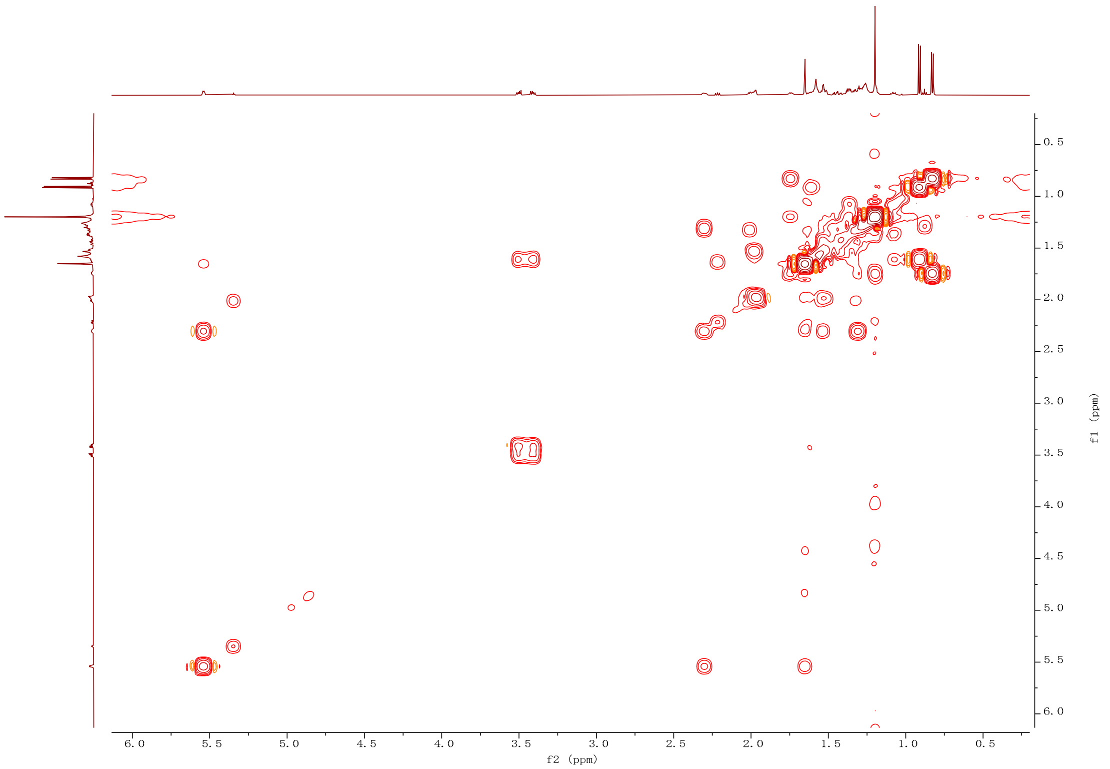

Fig. S4 ^{1}H-^{1}H COSY spectrum (600 MHz) of compound **1** in $CDCl_3$.

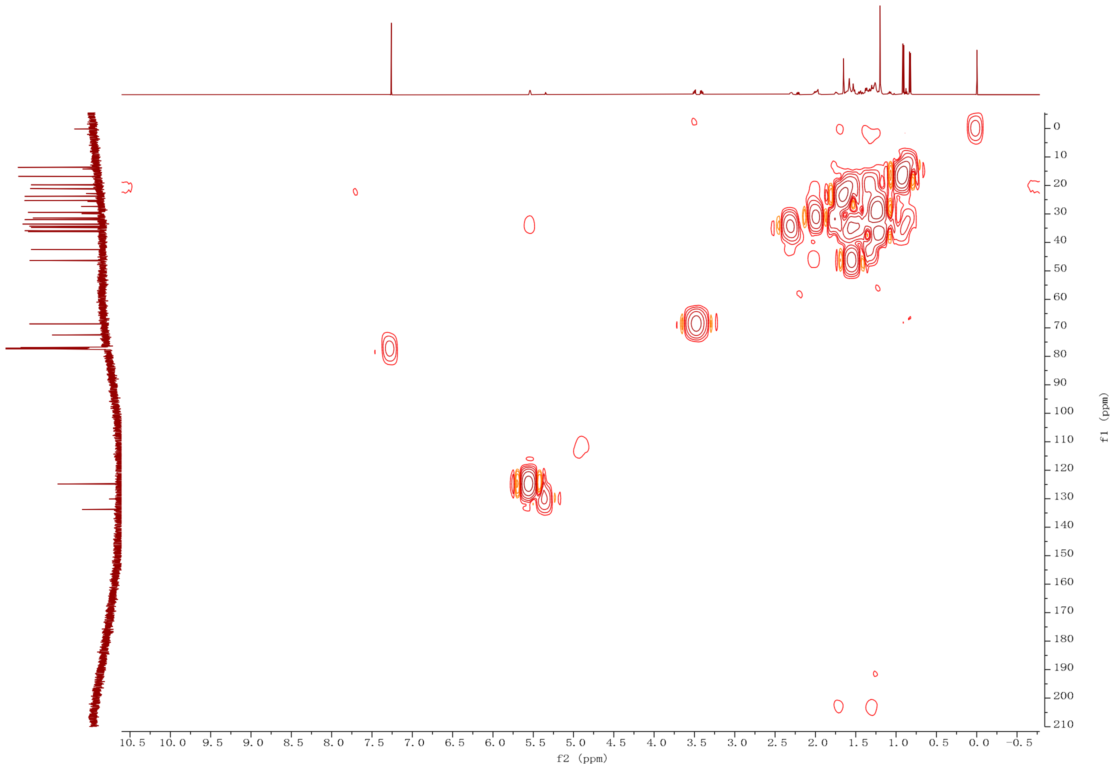

Fig. S5 HSQC spectrum (600 MHz) of compound **1** in $CDCl_3$.

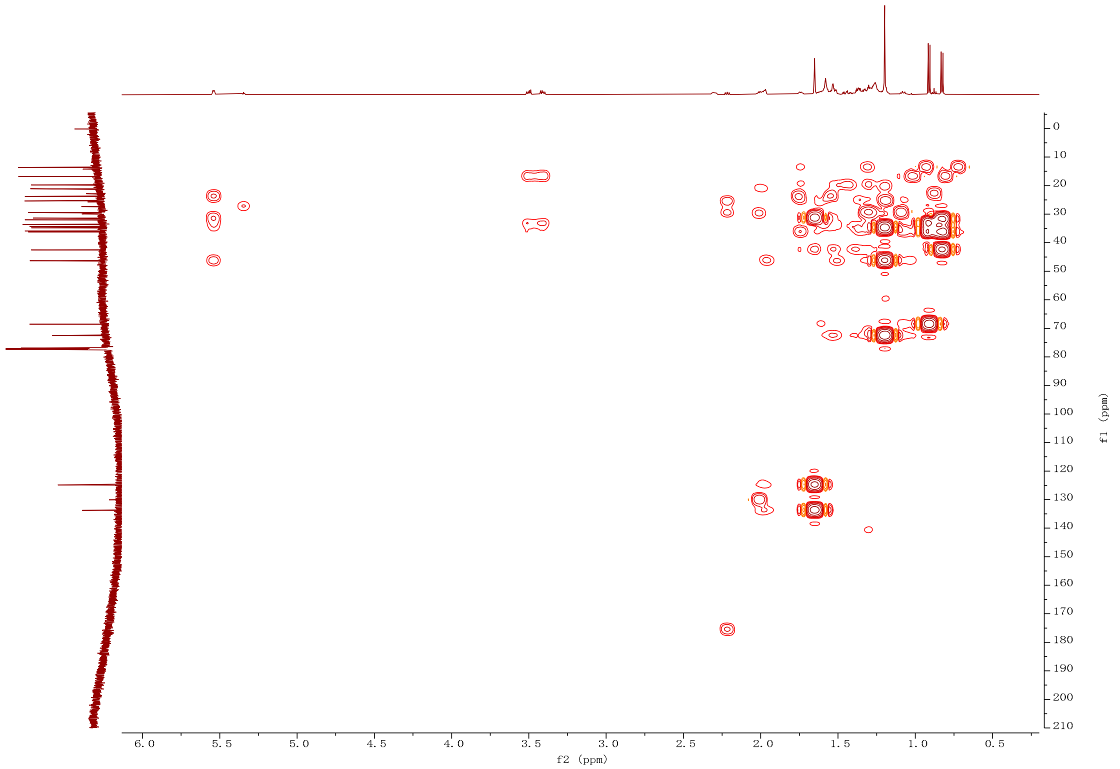

Fig. S6 HMBC spectrum (600 MHz) of compound **1** in $CDCl_3$.

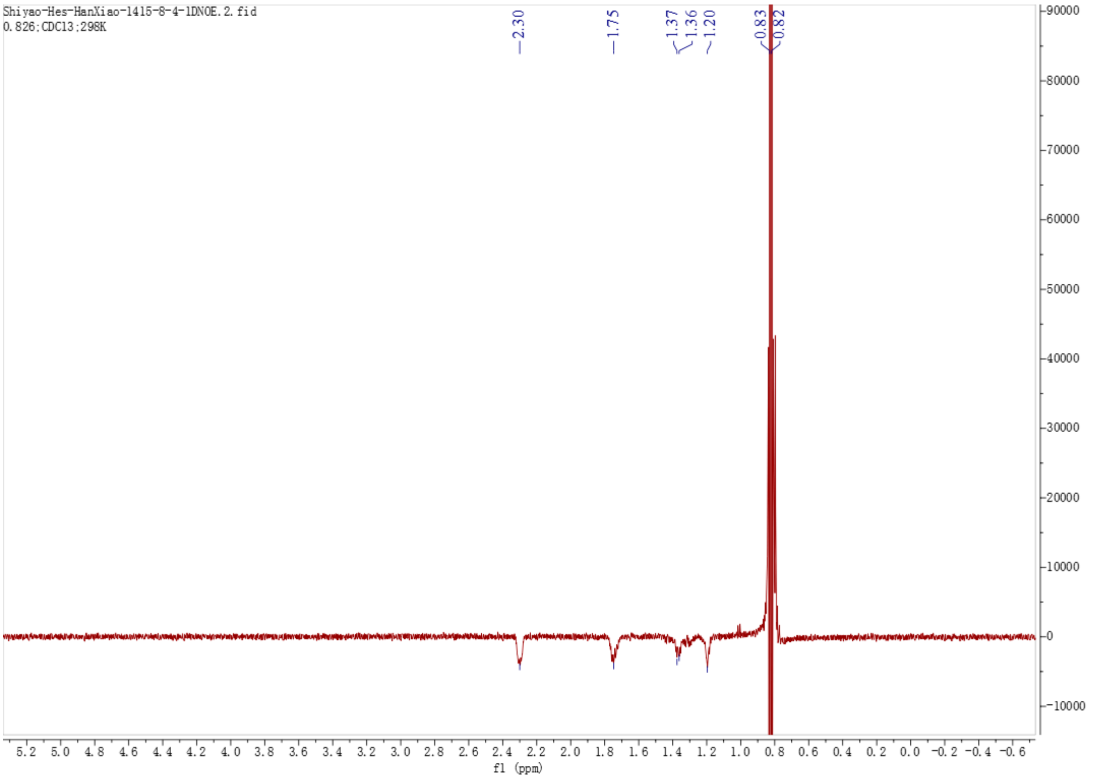

Fig. S7 [1]D -NOE (δ_H0.83) spectrum (600 MHz) of compound **1** in $CDCl_3$.

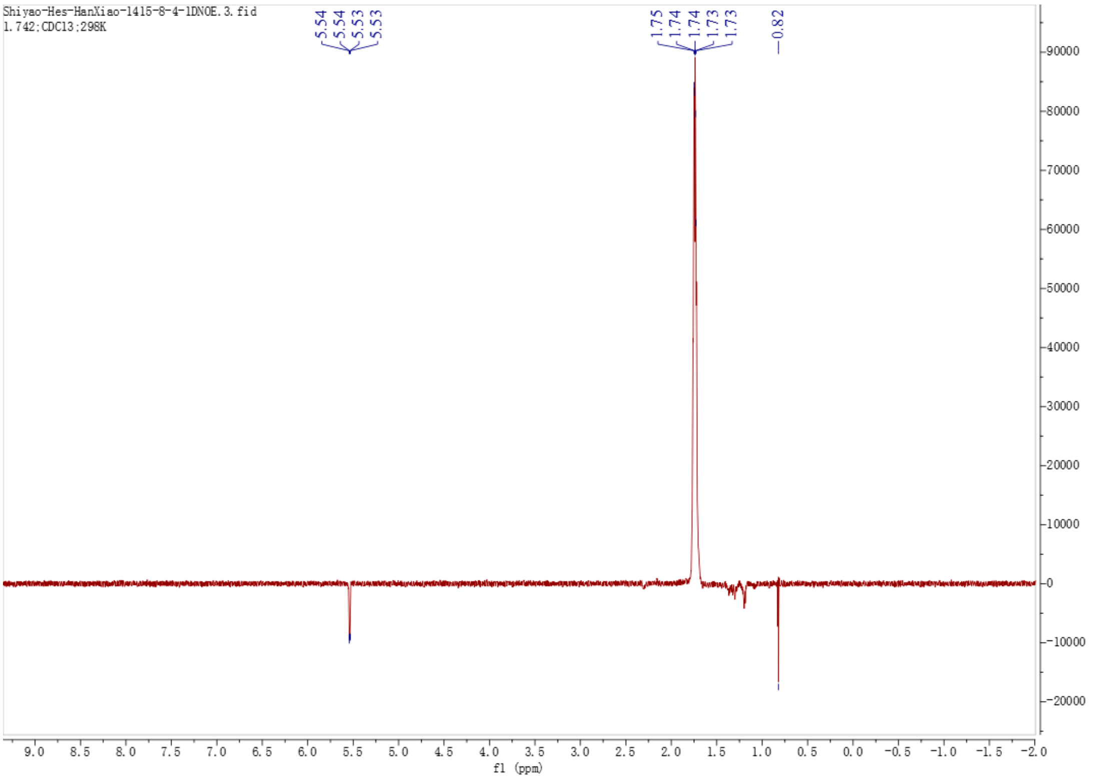

Fig. S8 [1]D -NOE (δ_H1.74) spectrum (600 MHz) of compound **1** in $CDCl_3$.

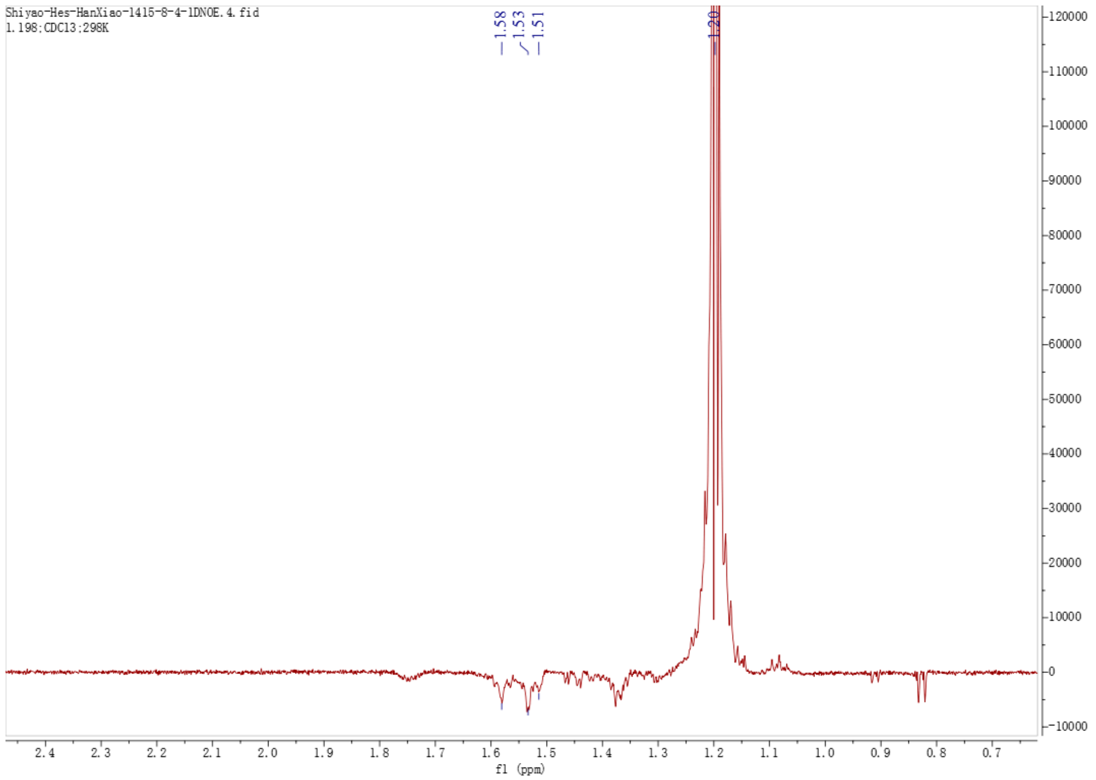

Fig. S9 [1]D -NOE (δ_H1.20) spectrum (600 MHz) of compound **1** in $CDCl_3$.

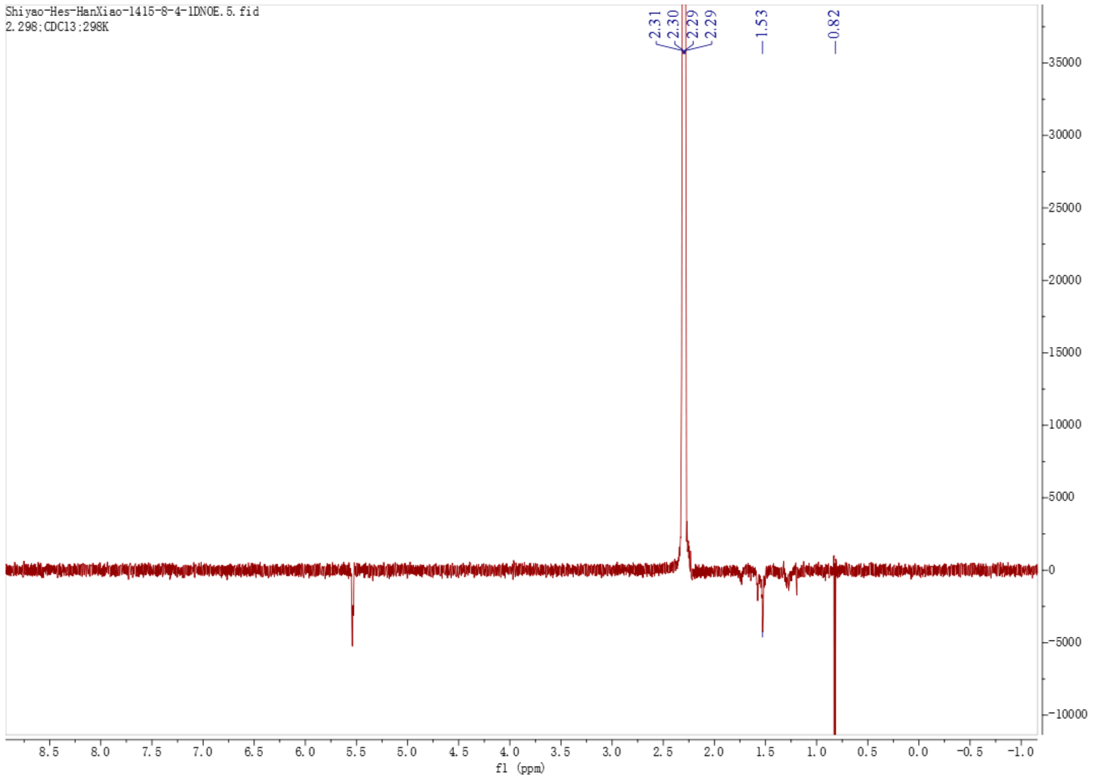

Fig. S10 [1]D -NOE (δ_H2.23) spectrum (600 MHz) of compound **1** in $CDCl_3$.

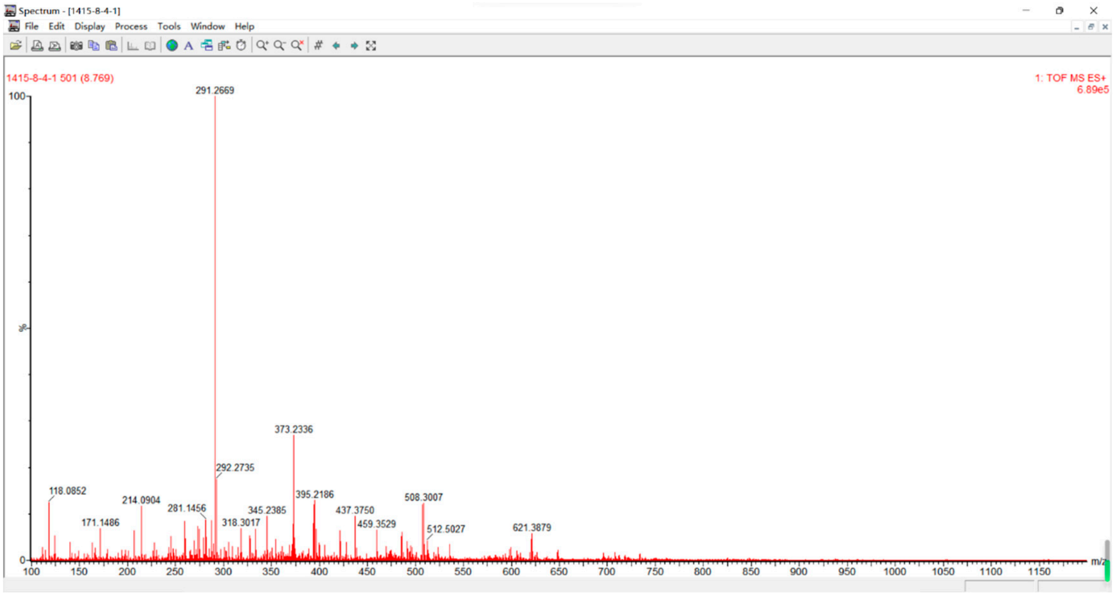

Fig. S11 HR-ESIMS spectrum of compound **1**.

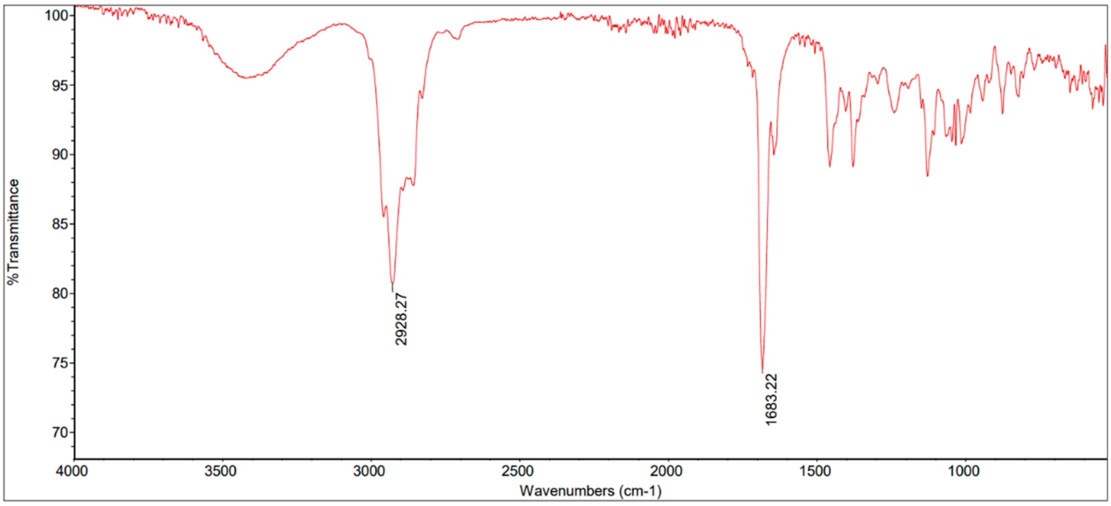

Fig. S12 Infrared spectrum of compound **1**.

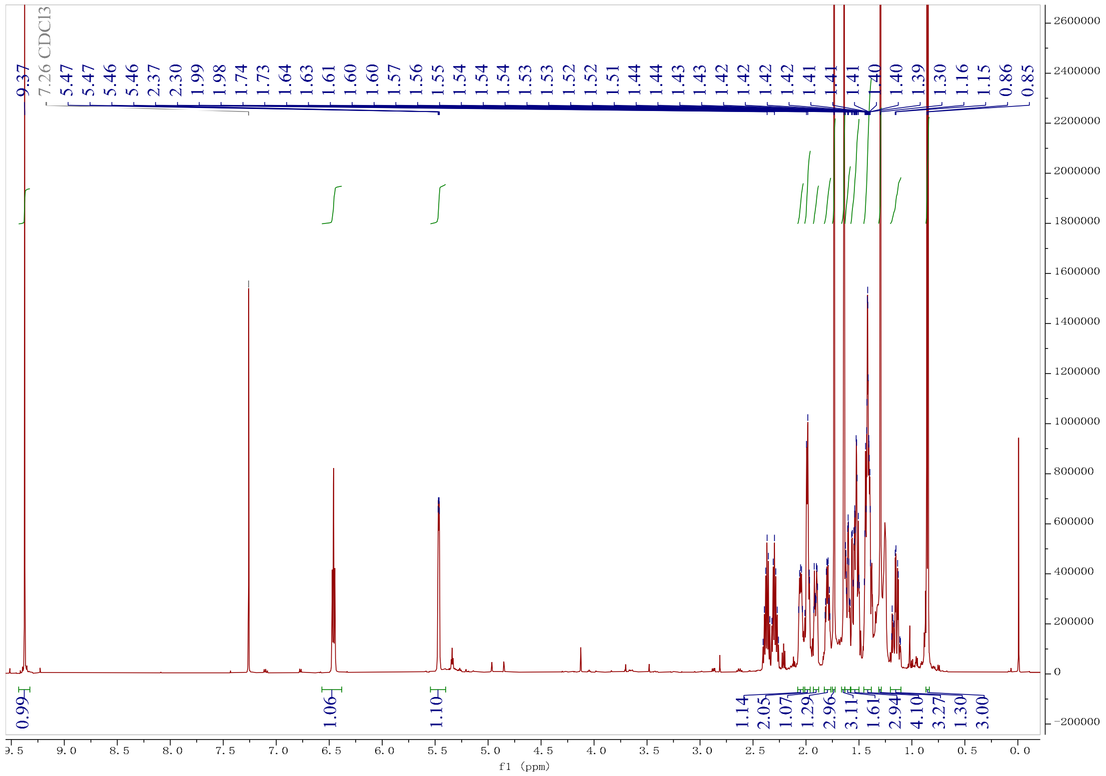

Fig. S13 ^{1}H NMR spectrum (600 MHz) of compound **2** in $CDCl_3$.

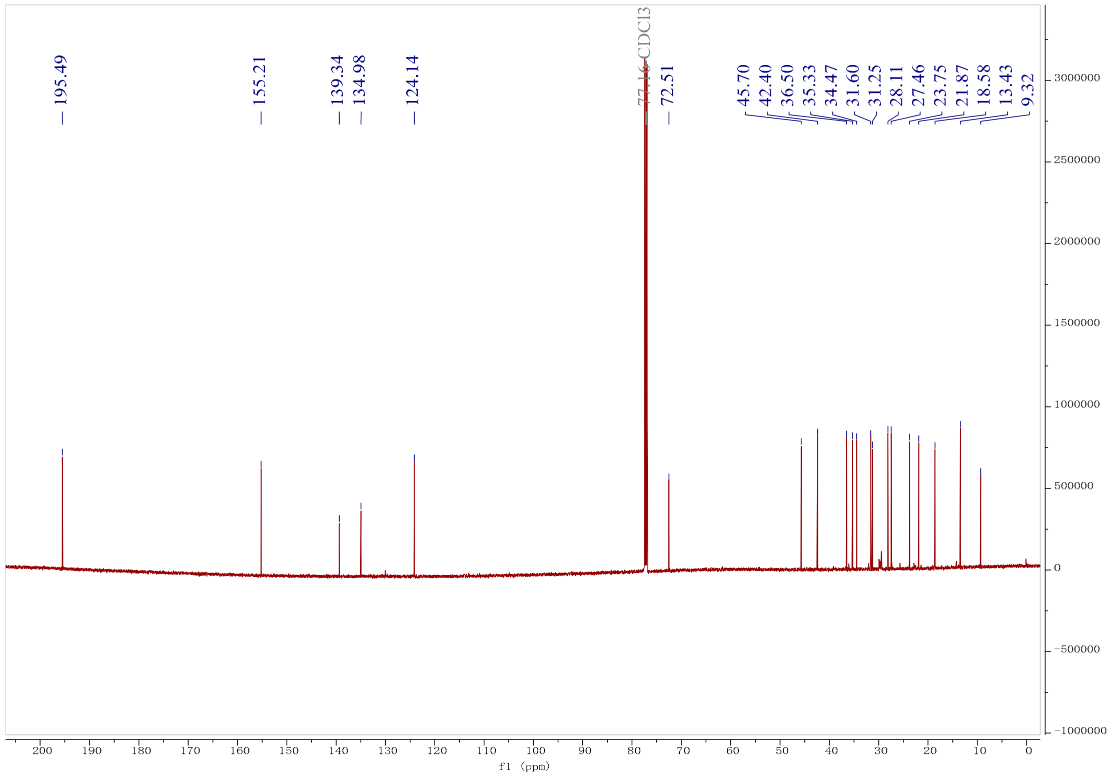

Fig. S14 ^{13}C NMR spectrum (150 MHz) of compound **2** in $CDCl_3$.

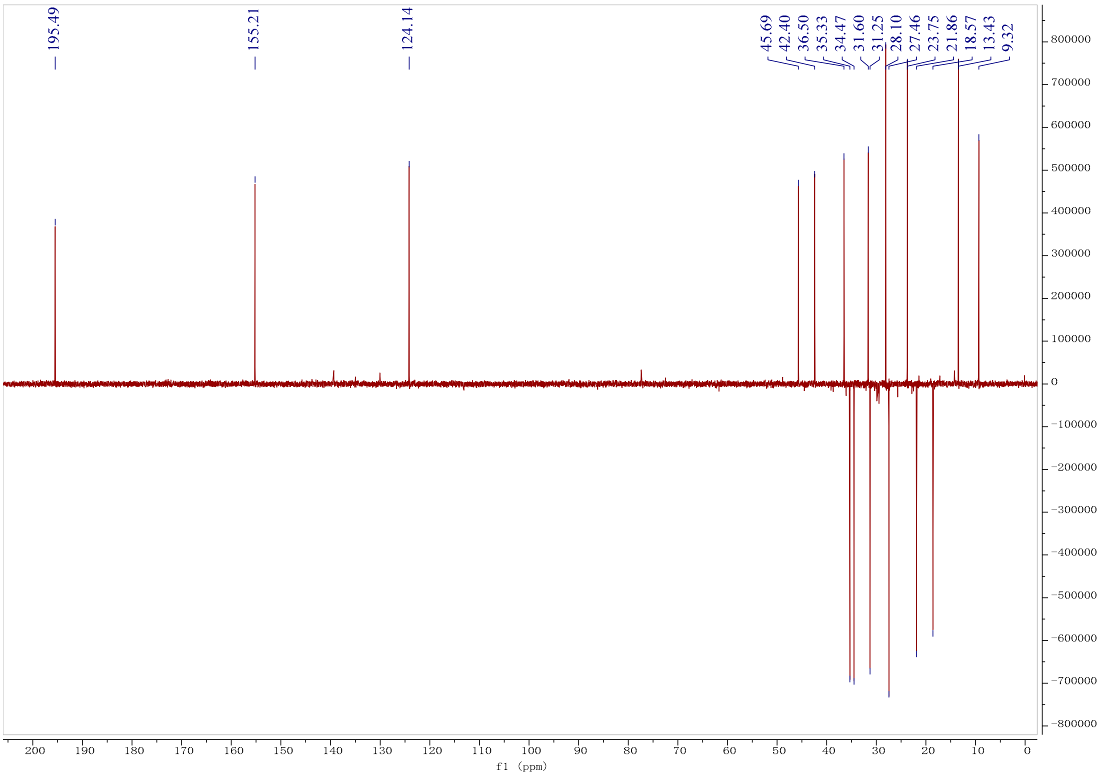

Fig. S15 DEPT spectrum (150 MHz) of compound **2** in $CDCl_3$.

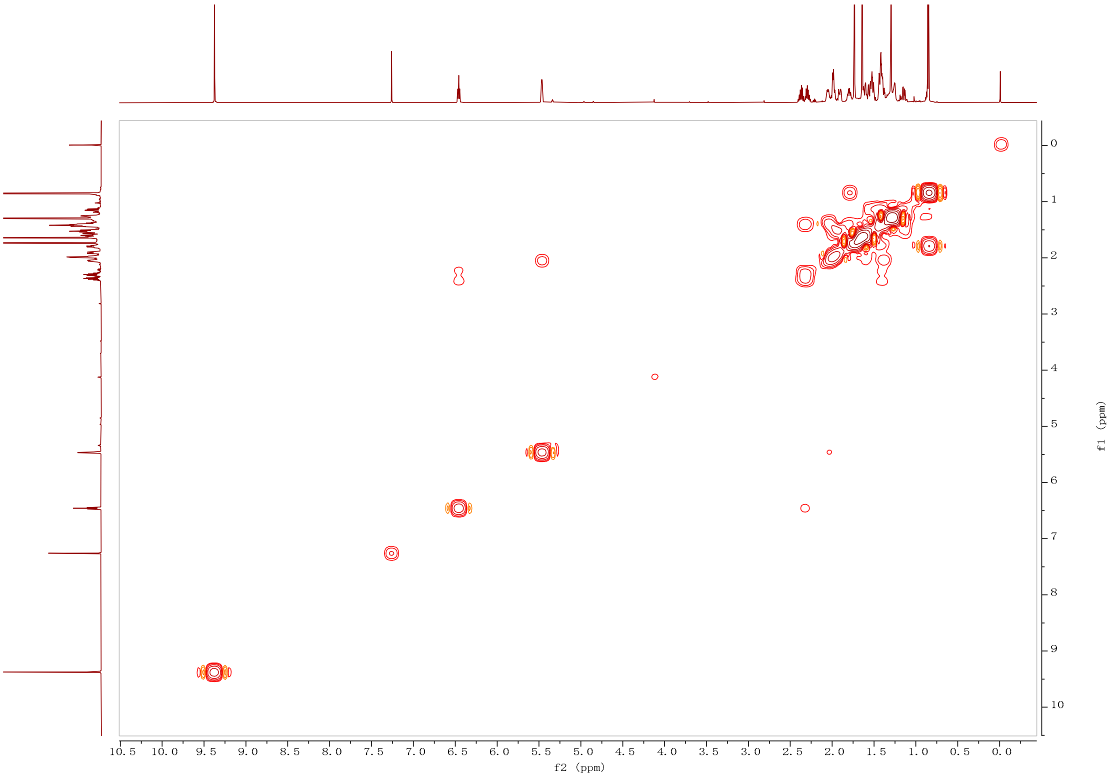

Fig. S16 ^{1}H-^{1}H COSY spectrum (600 MHz) of compound **2** in $CDCl_3$.

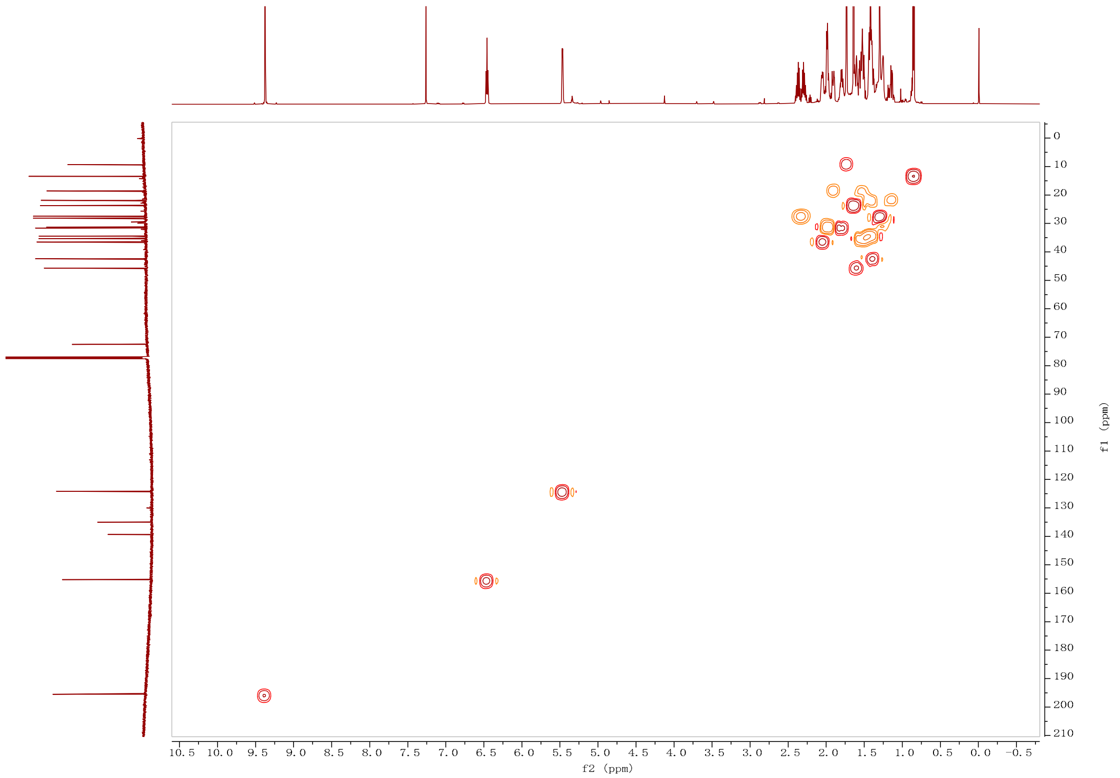

Fig. S17 HSQC spectrum (600 MHz) of compound **2** in $CDCl_3$.

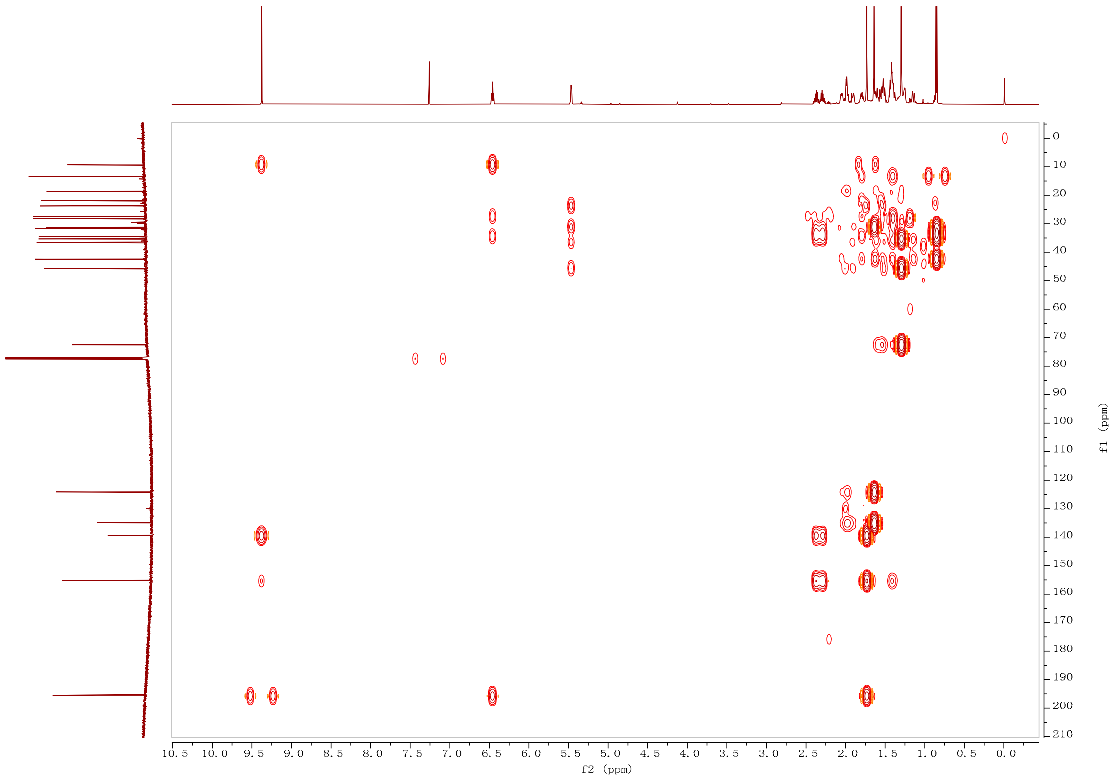

Fig. S18 HMBC spectrum (600 MHz) of compound **2** in $CDCl_3$.

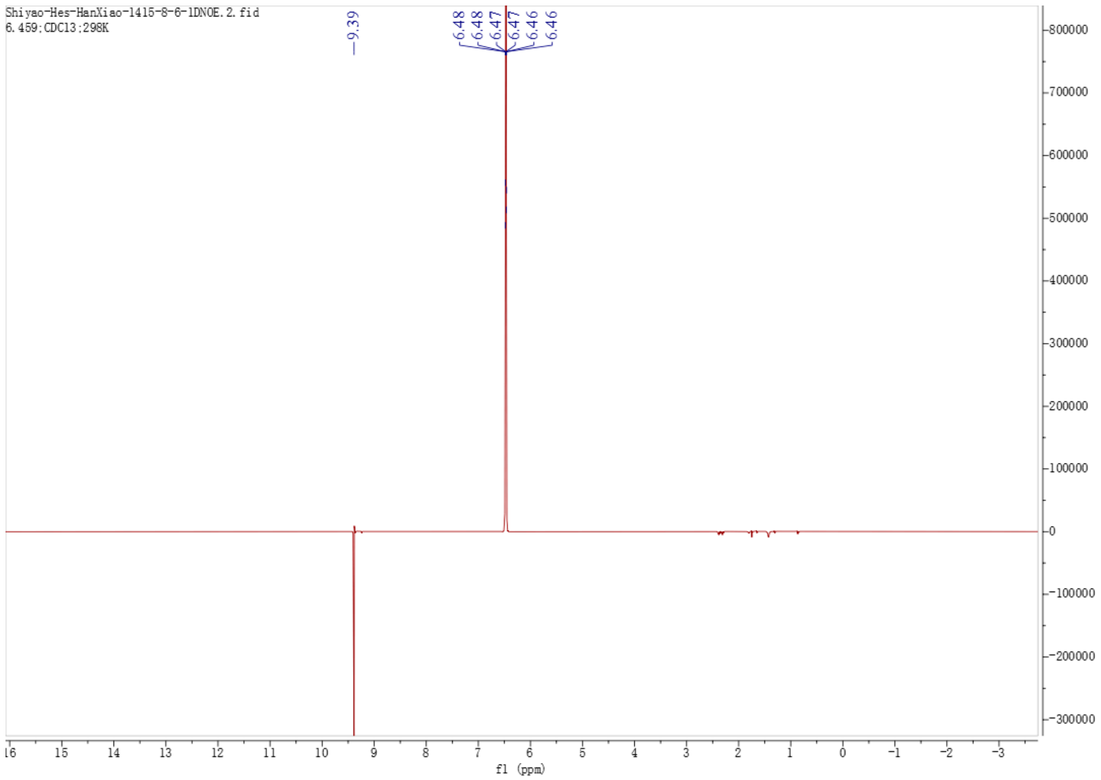

Fig. S19 [1]D-NOE (δ_H2.23) spectrum (600 MHz) of compound **2** in $CDCl_3$.

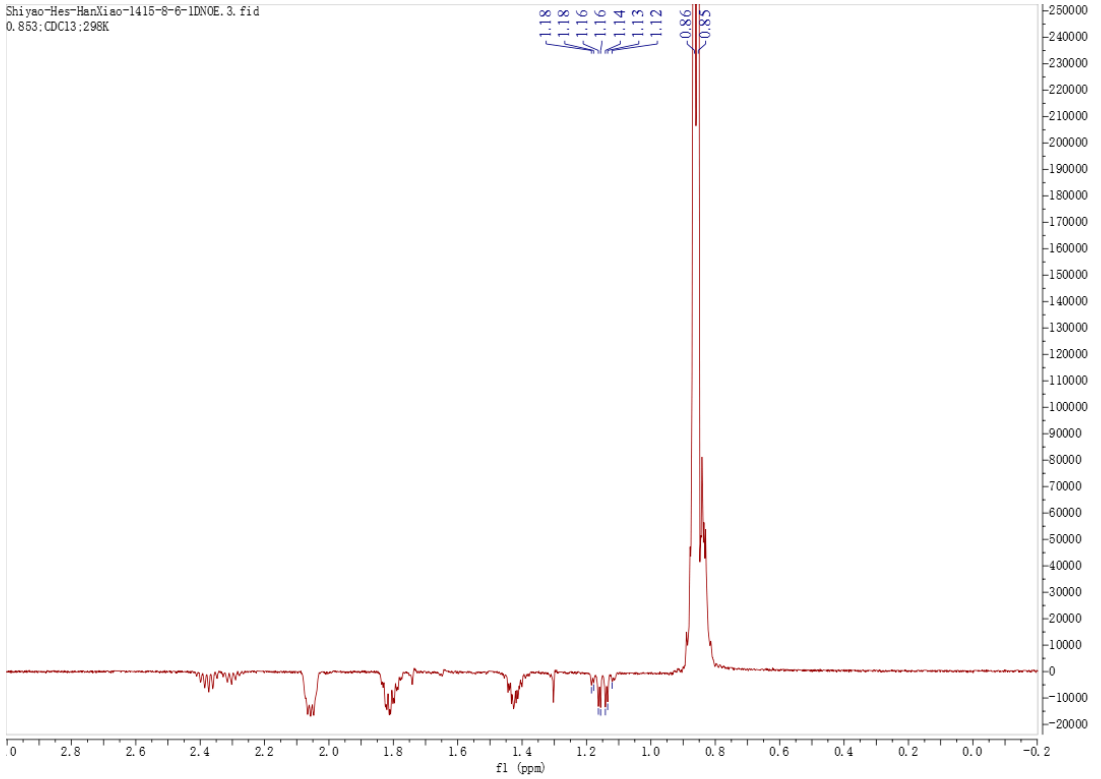

Fig. S20 [1]D -NOE (δ_H0.85) spectrum (600 MHz) of compound **2** in $CDCl_3$.

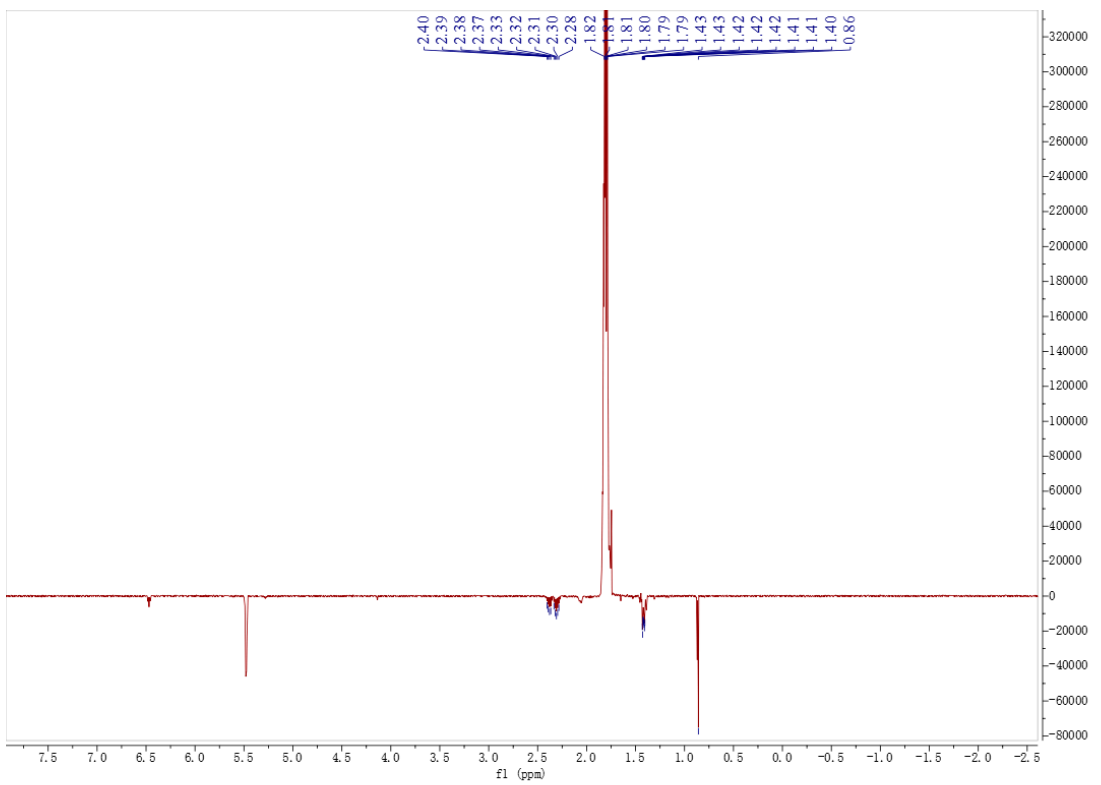

Fig. S21 [1]D -NOE (δ_H1.80) spectrum (600 MHz) of compound **2** in $CDCl_3$.

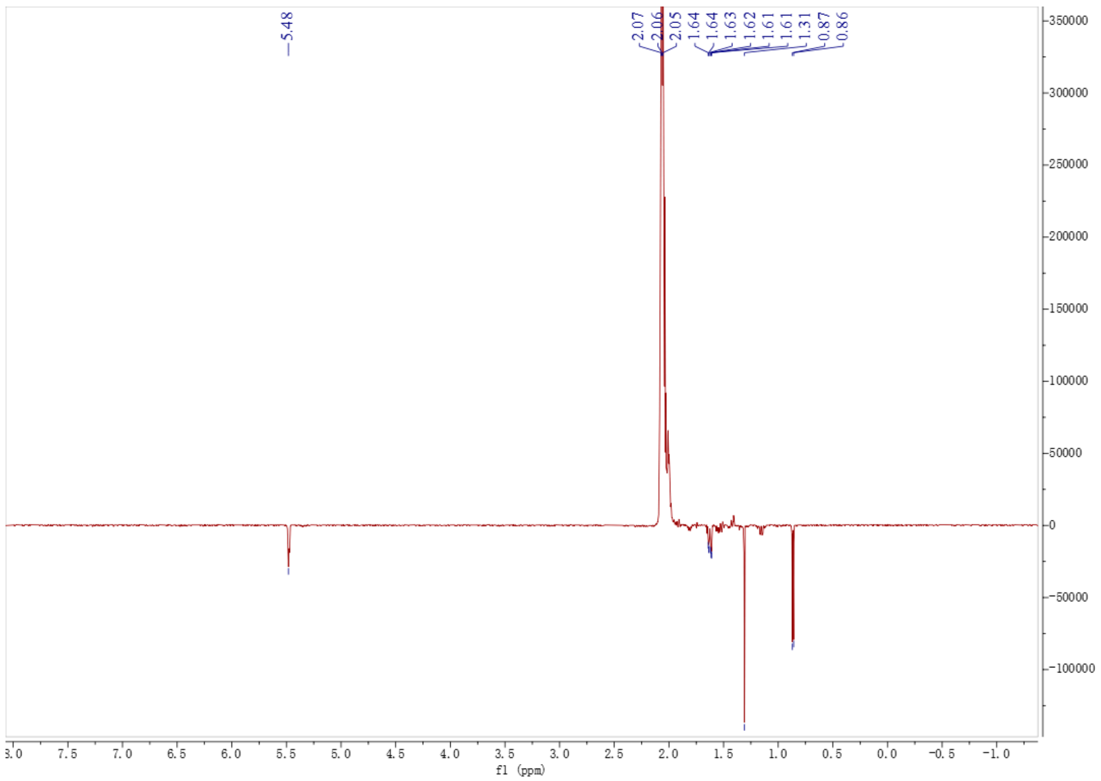

Fig. S22 [1]D -NOE (δ_H2.05) spectrum (600 MHz) of compound **2** in $CDCl_3$.

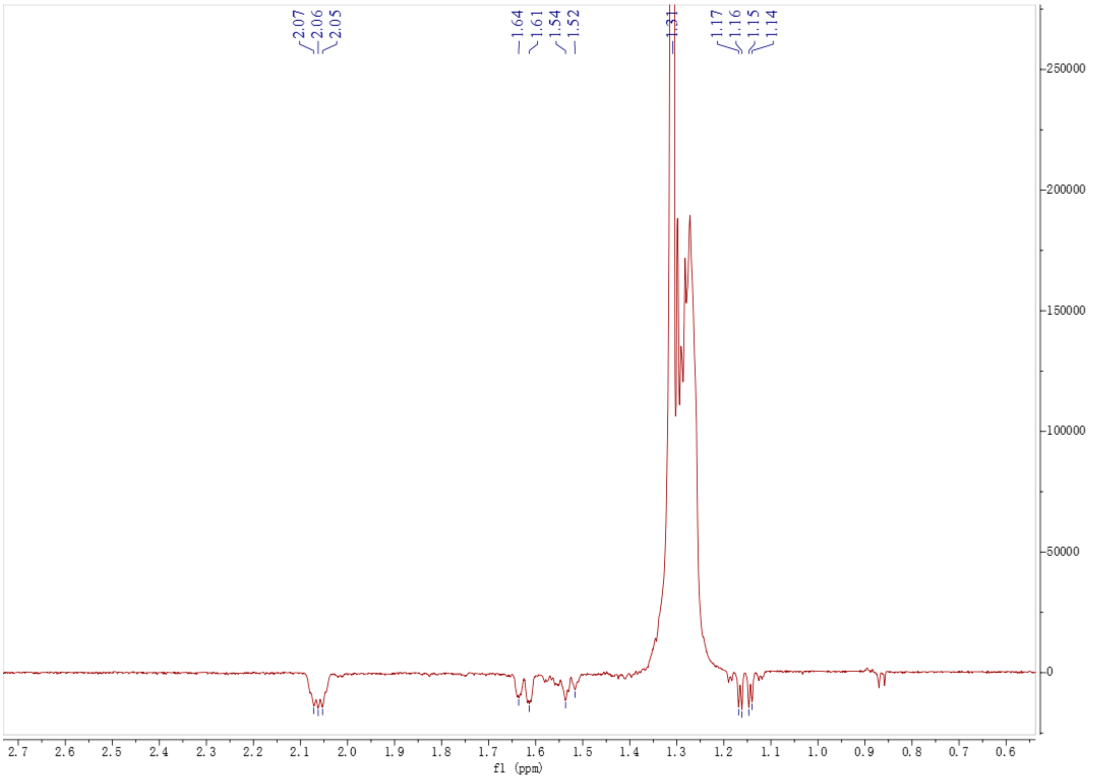

Fig. S23 [1]D -NOE (δ_H1.30) spectrum (600 MHz) of compound **2** in $CDCl_3$.

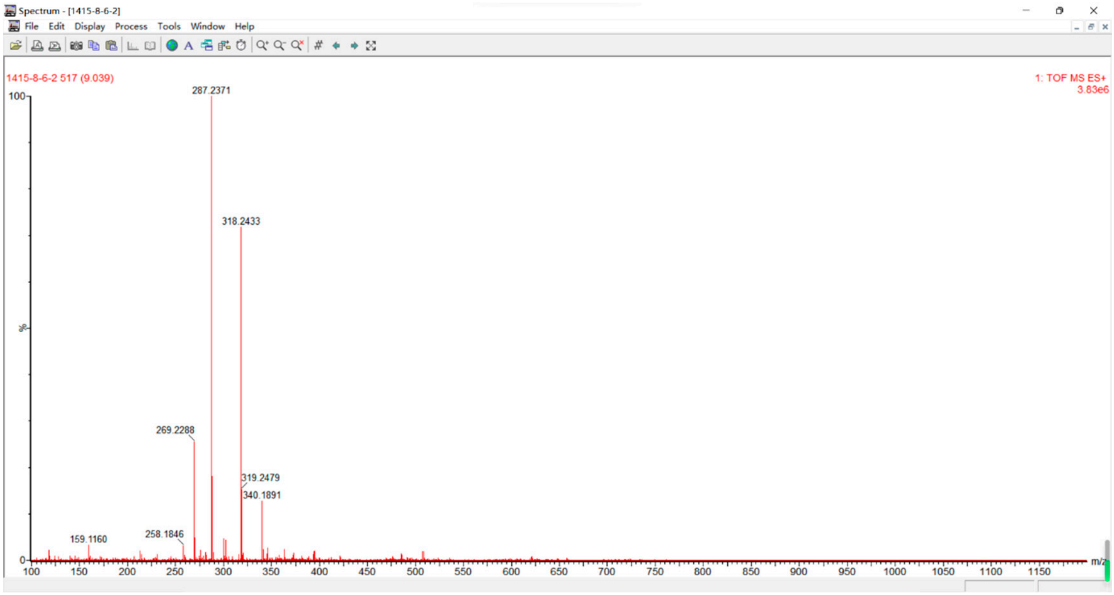

Fig. S24 HR-ESIMS spectrum of compound **2**.

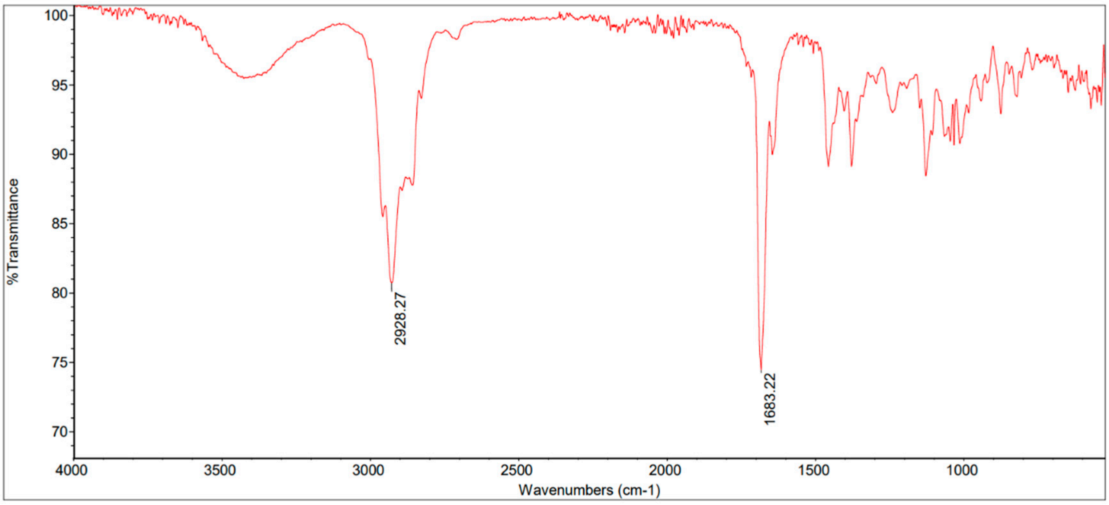

Fig. S25 Infrared spectrum of compound **2**.

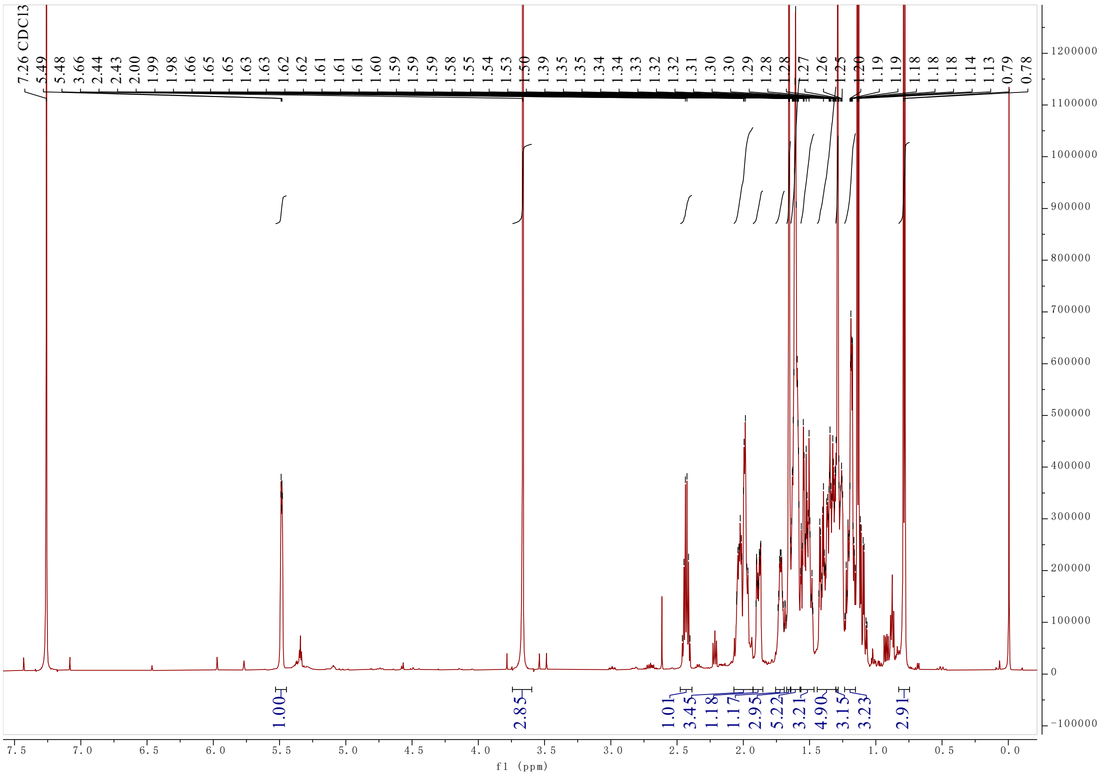

Fig. S26 ^{1}H NMR spectrum (600 MHz) of compound **3** in $CDCl_3$.

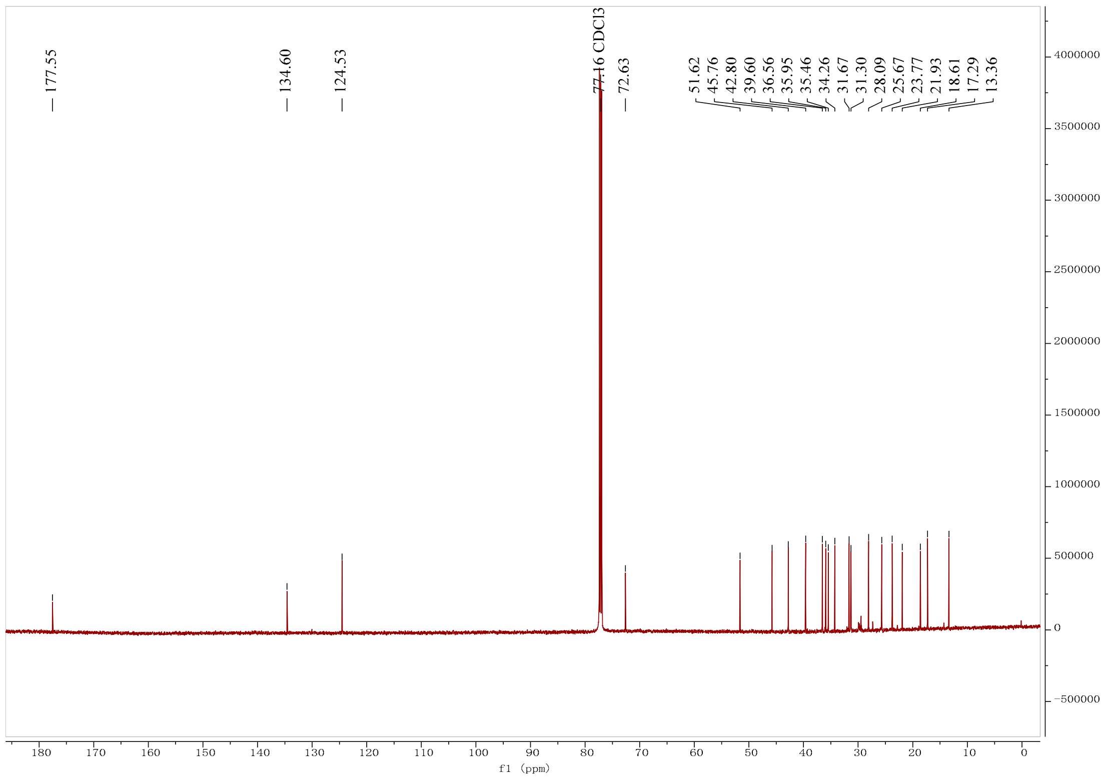

Fig. S27 ^{13}C NMR spectrum (150 MHz) of compound **3** in $CDCl_3$.

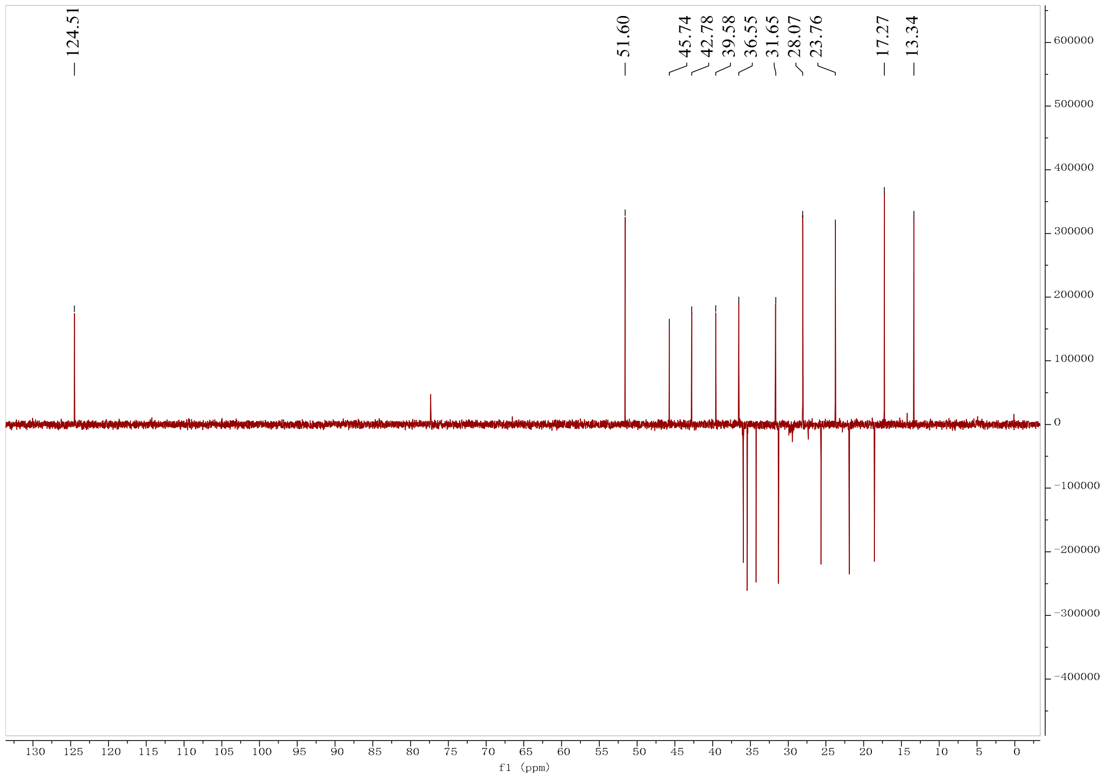

Fig. S28 DEPT spectrum (150 MHz) of compound **3** in $CDCl_3$.

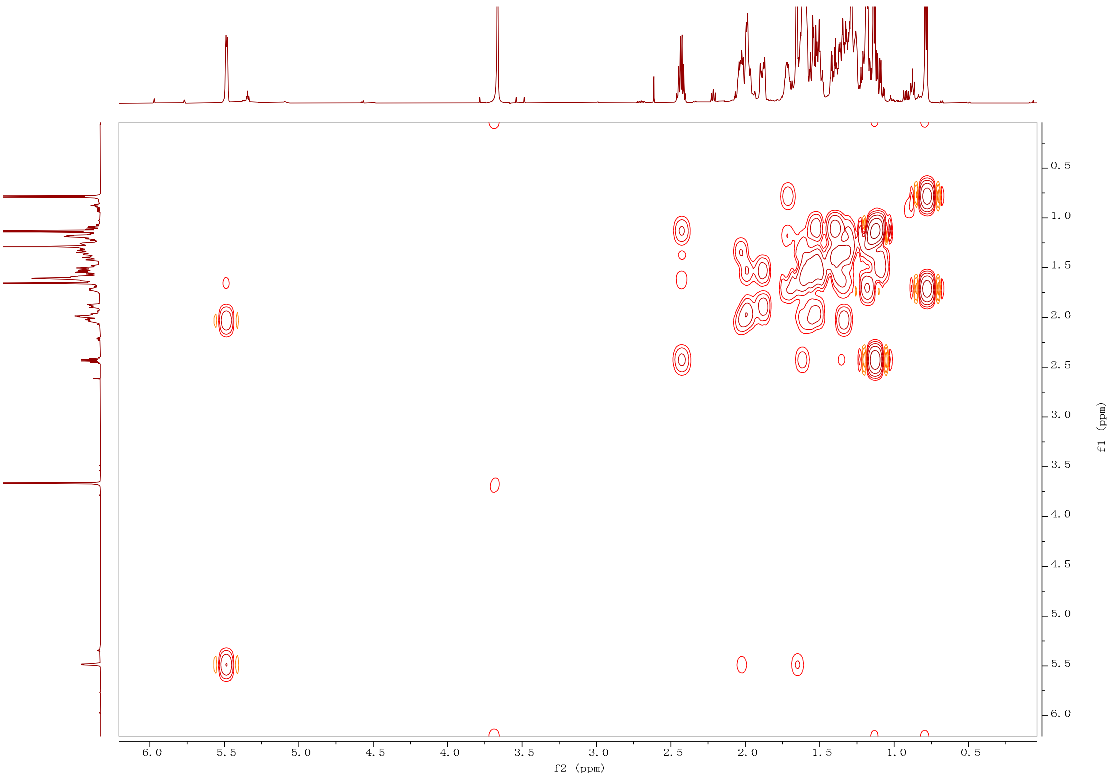

Fig. S29 ^{1}H-^{1}H COSY spectrum (600 MHz) of compound **3** in $CDCl_3$.

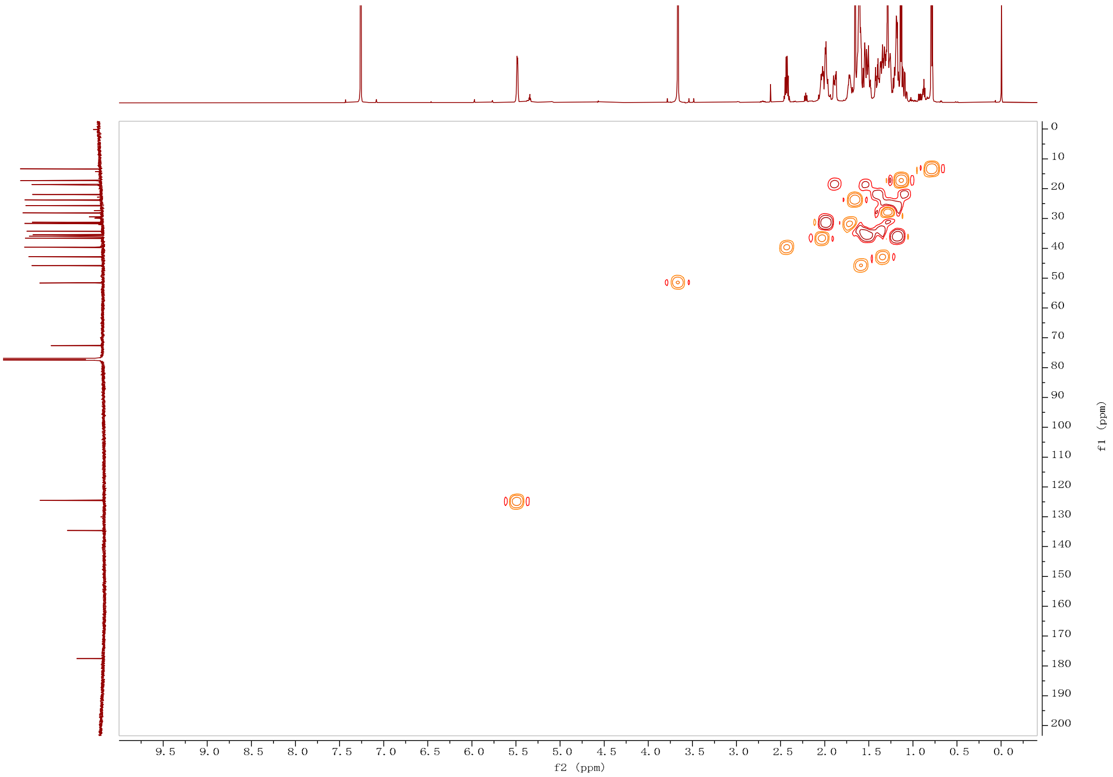

Fig. S30 HSQC spectrum (600 MHz) of compound **3** in $CDCl_3$.

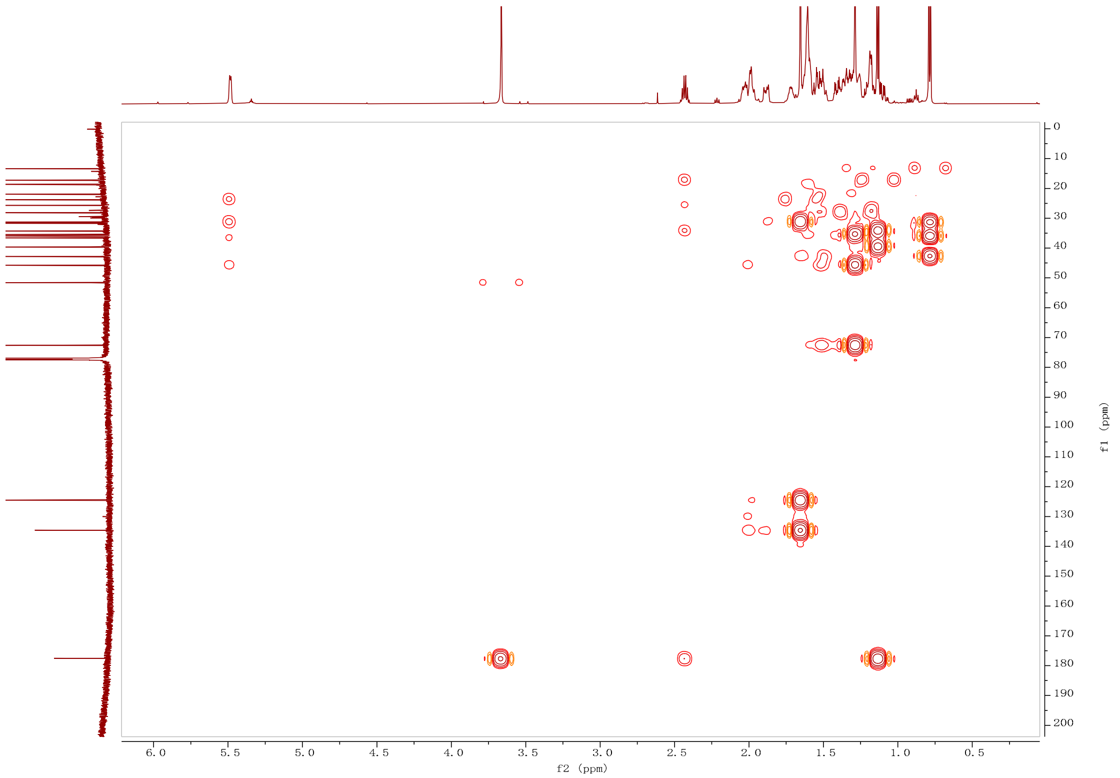

Fig. S31 HMBC spectrum (600 MHz) of compound **3** in $CDCl_3$.

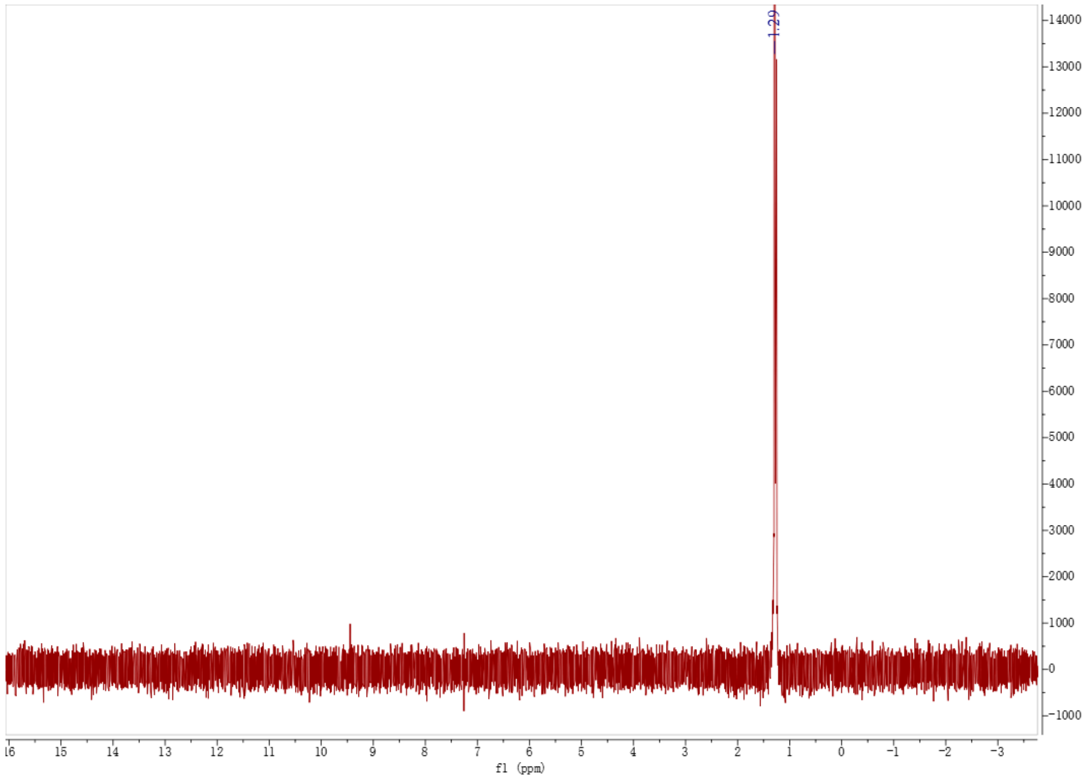

Fig. S32 [1]D -NOE (δ_H1.29) spectrum (600 MHz) of compound **3** in $CDCl_3$.

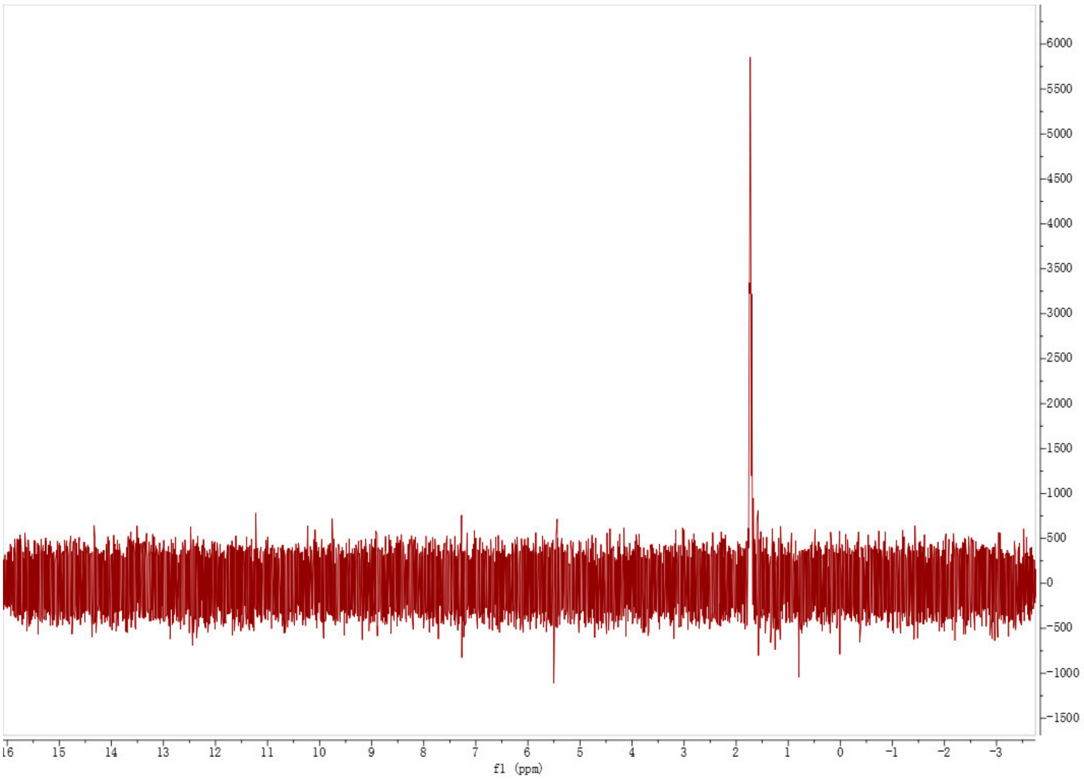

Fig. S33 [1]D -NOE (δ_H1.72) spectrum (600 MHz) of compound **3** in $CDCl_3$.

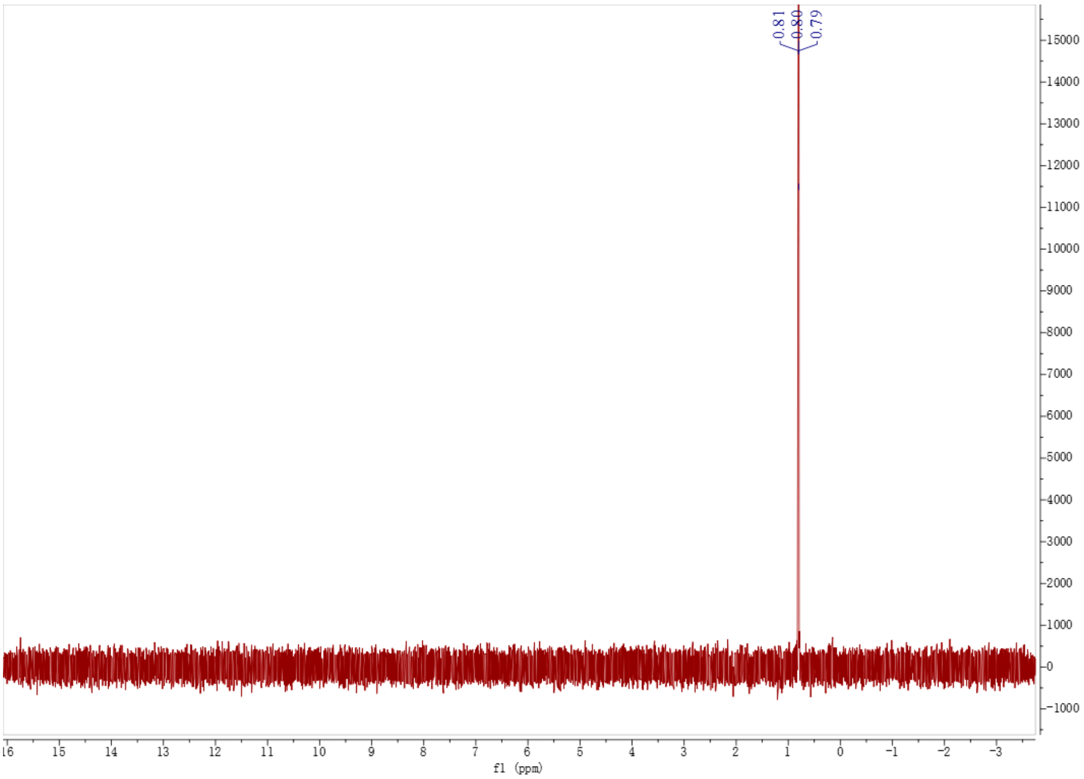

Fig. S34 [1]D -NOE (δ_H0.79) spectrum (600 MHz) of compound **3** in $CDCl_3$.

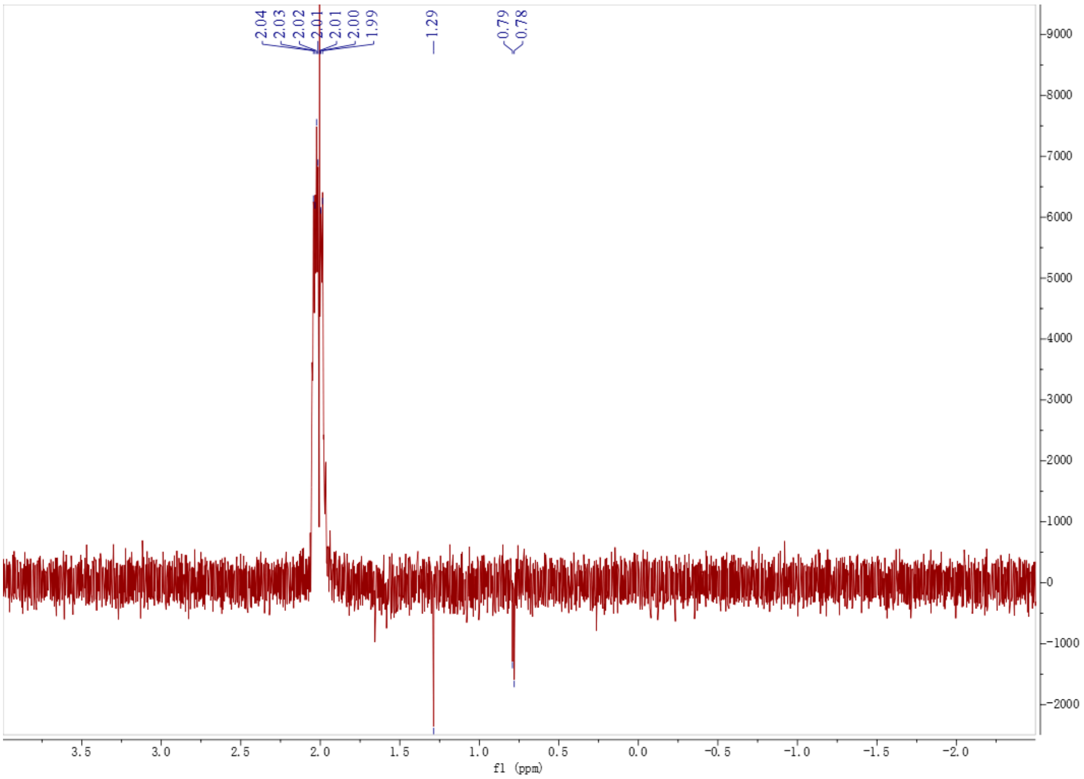

Fig. S35 [1]D -NOE (δ_H2.03) spectrum (600 MHz) of compound **3** in $CDCl_3$.

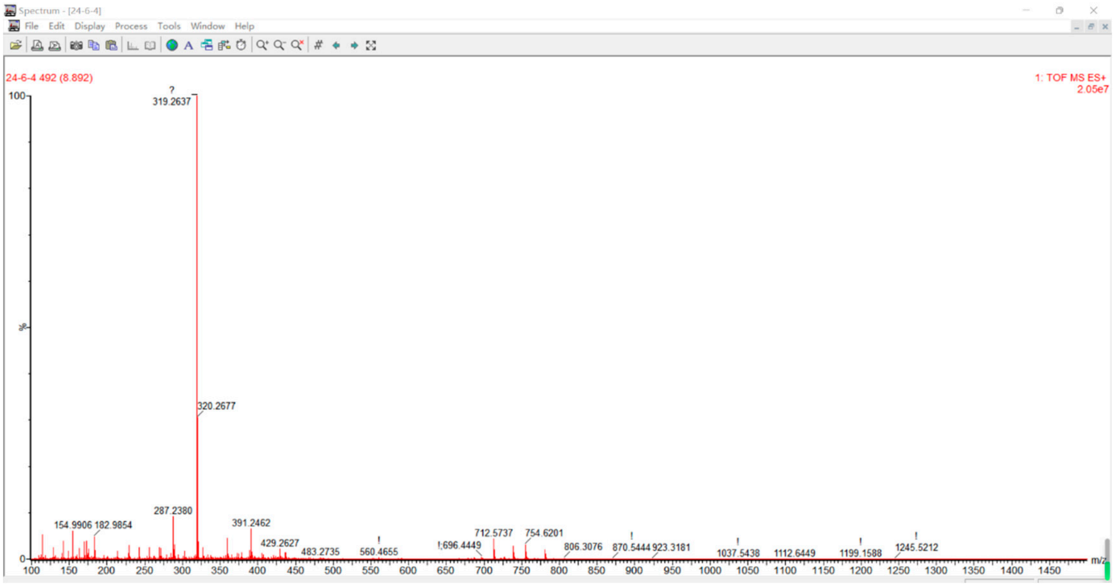

Fig. S36 HR-ESIMS spectrum of compound **3**.

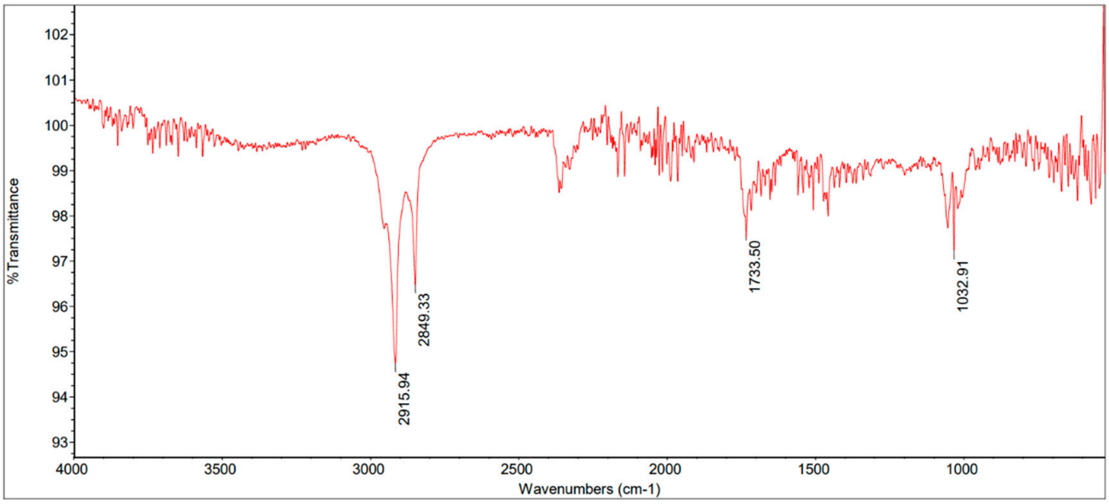

Fig. S37 Infrared spectrum of compound **3**.

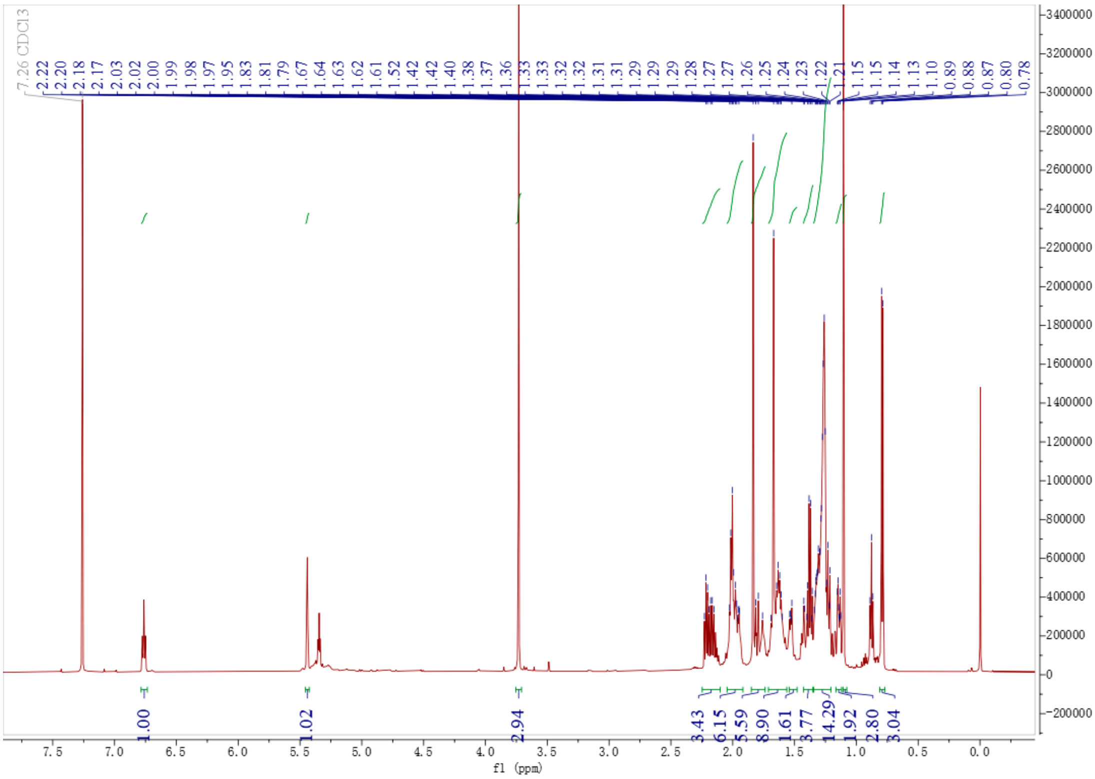

Fig. S38 ^{1}H NMR spectrum (600 MHz) of compound **4** in $CDCl_3$.

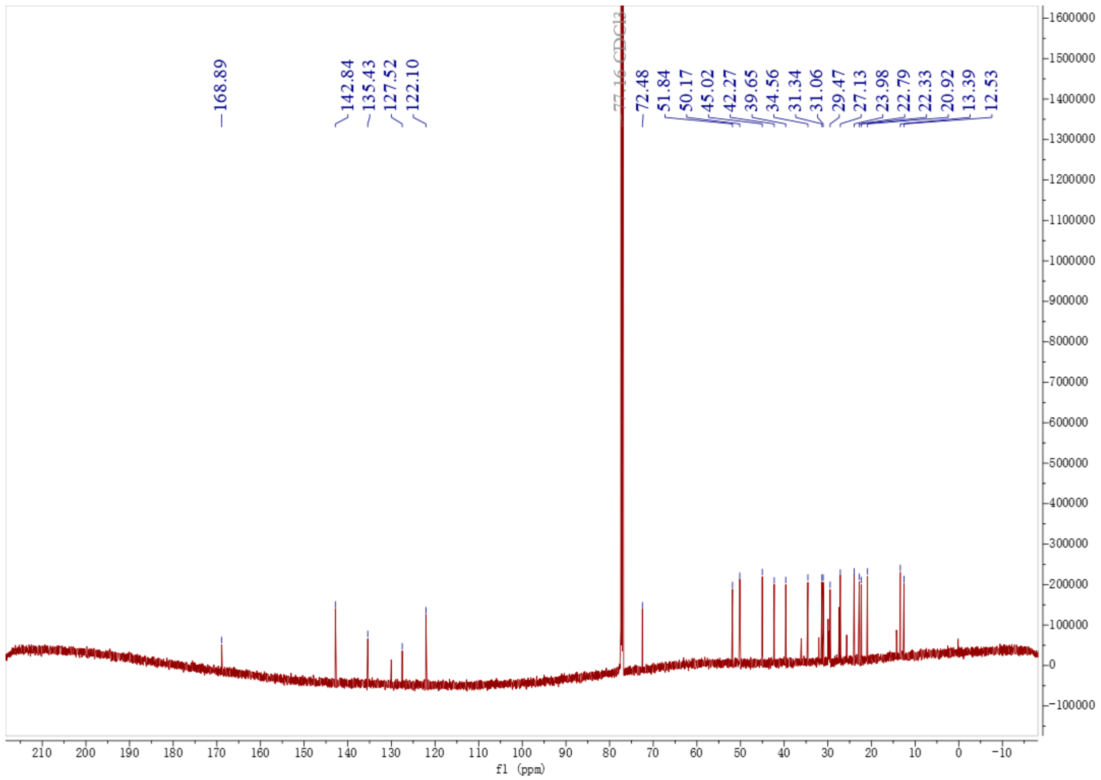

Fig. S39 ^{13}C NMR spectrum (150 MHz) of compound **4** in $CDCl_3$.

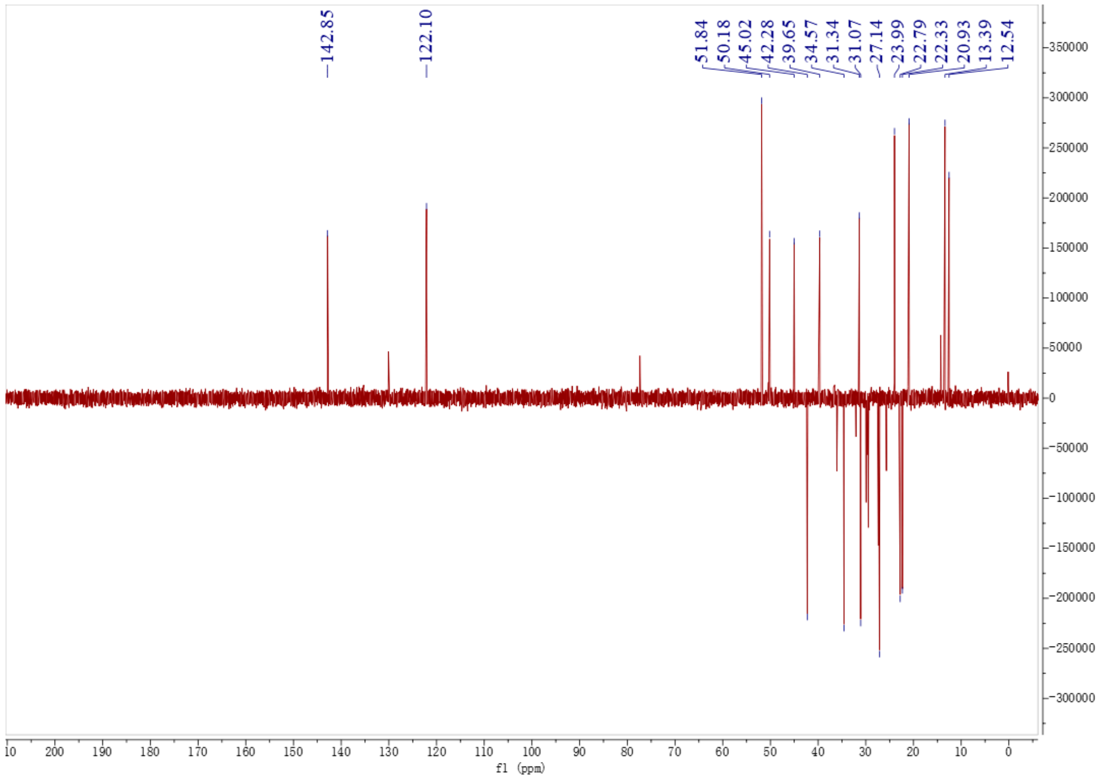

Fig. S40 DEPT spectrum (150 MHz) of compound **4** in $CDCl_3$.

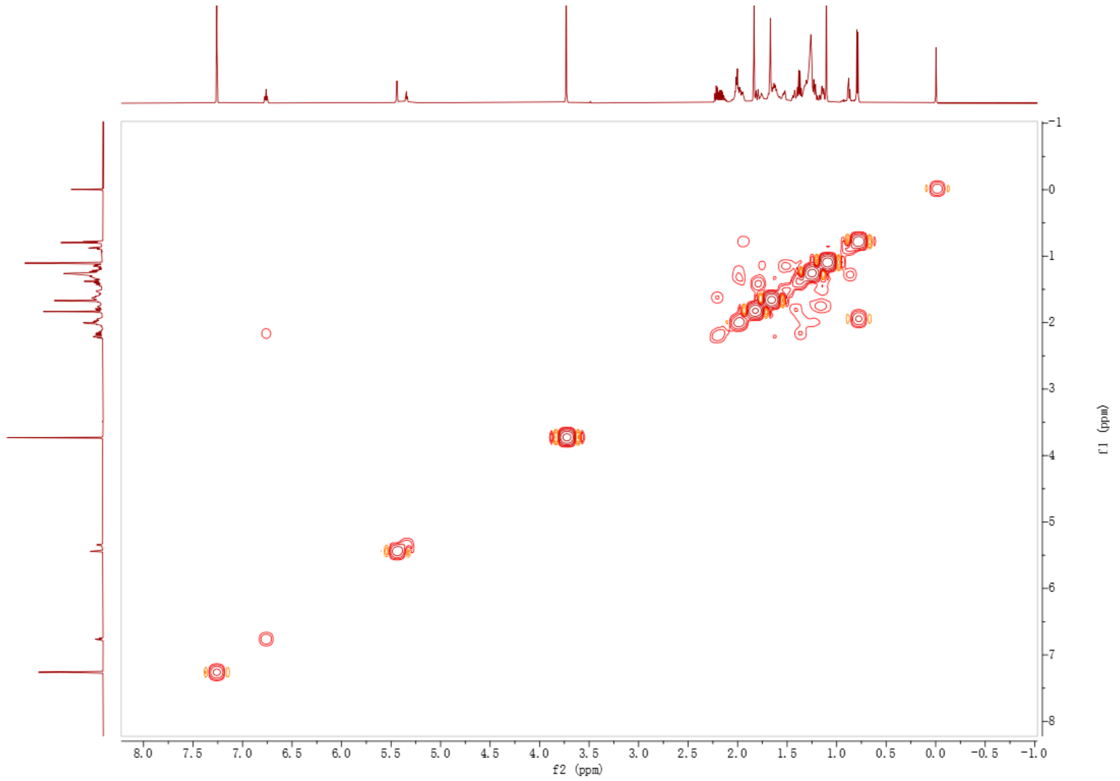

Fig. S41 ^{1}H-^{1}H COSY spectrum (600 MHz) of compound **4** in $CDCl_3$

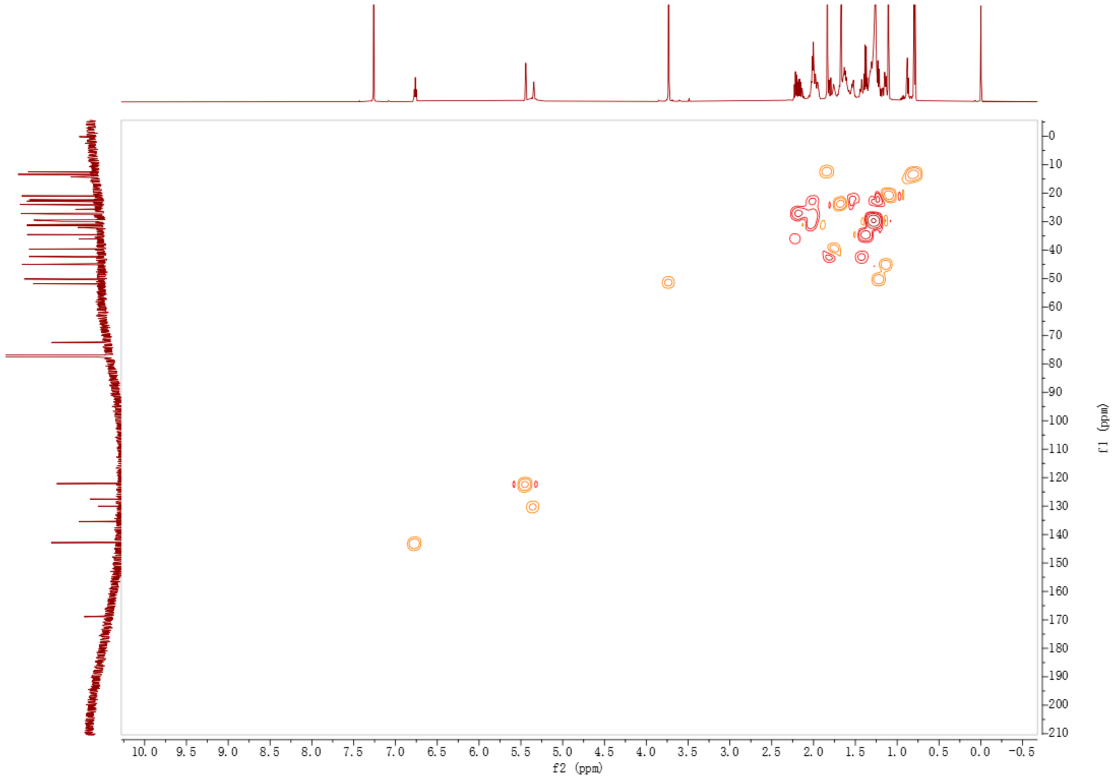

Fig. S42 HSQC spectrum (600 MHz) of compound **4** in $CDCl_3$.

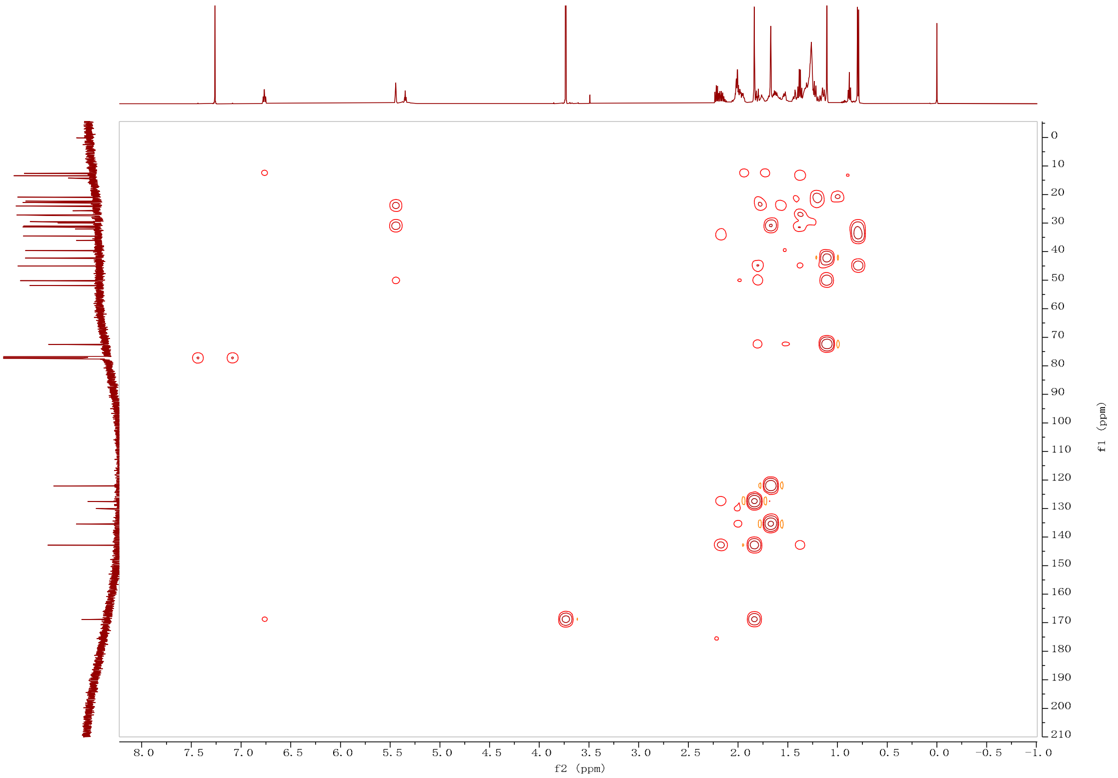

Fig. S43 HMBC spectrum (600 MHz) of compound **4** in $CDCl_3$.

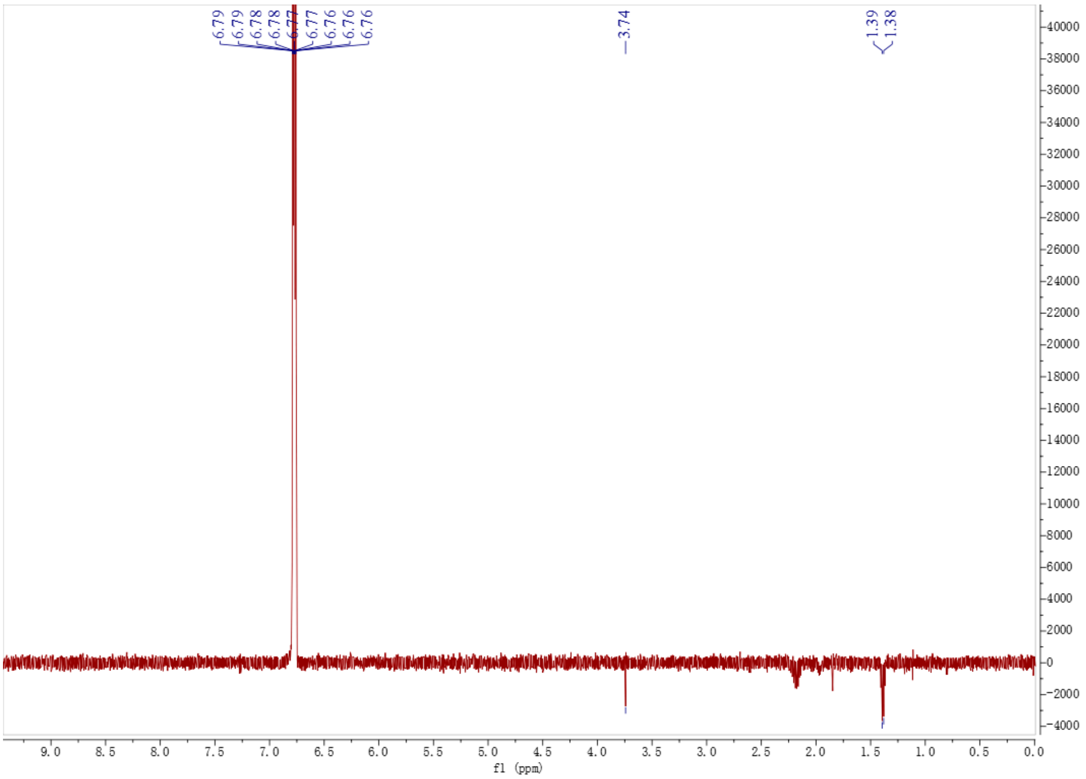

Fig. S44 [1]D -NOE (δ_H6.76) spectrum (600 MHz) of compound **4** in $CDCl_3$.

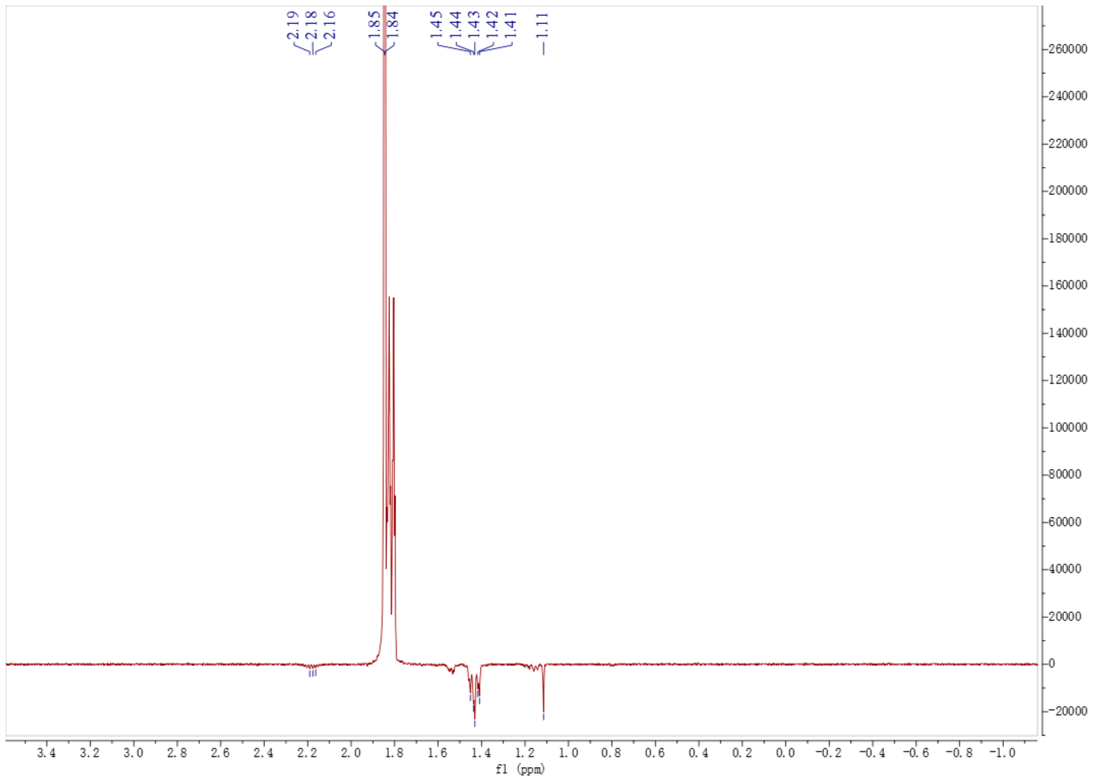

Fig. S45 [1]D -NOE (δ_H1.83) spectrum (600 MHz) of compound **4** in $CDCl_3$.

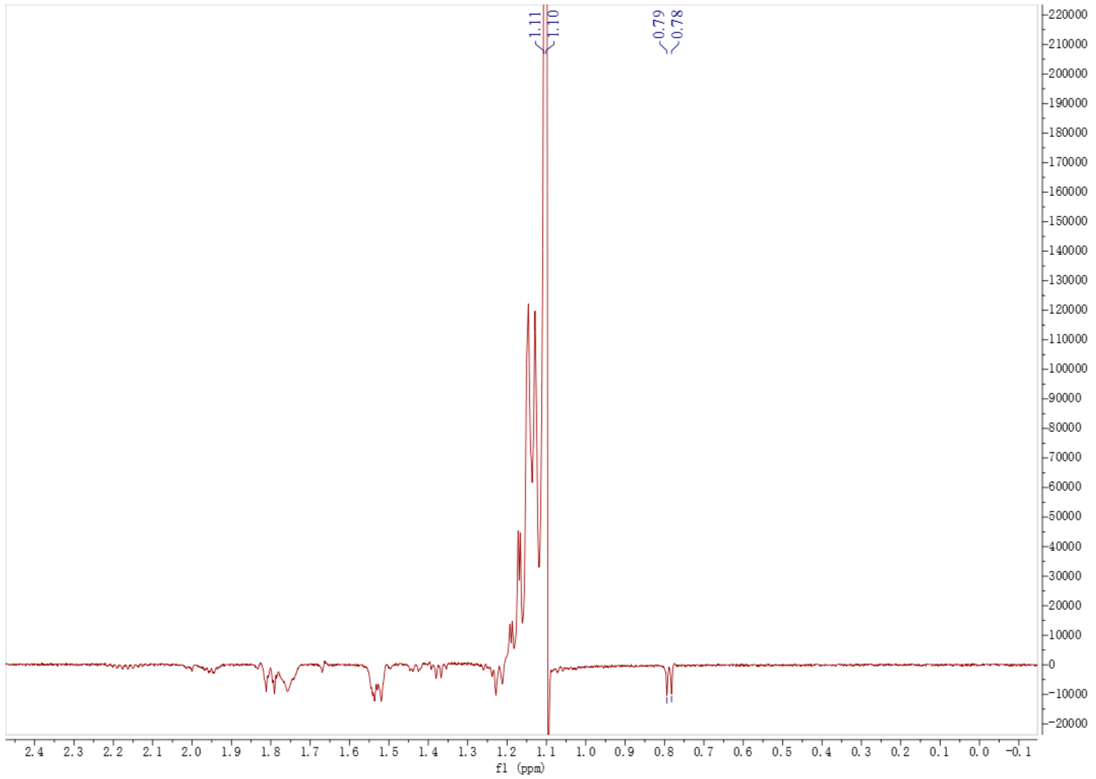

Fig. S46 [1]D -NOE (δ_H1.14) spectrum (600 MHz) of compound **4** in $CDCl_3$.

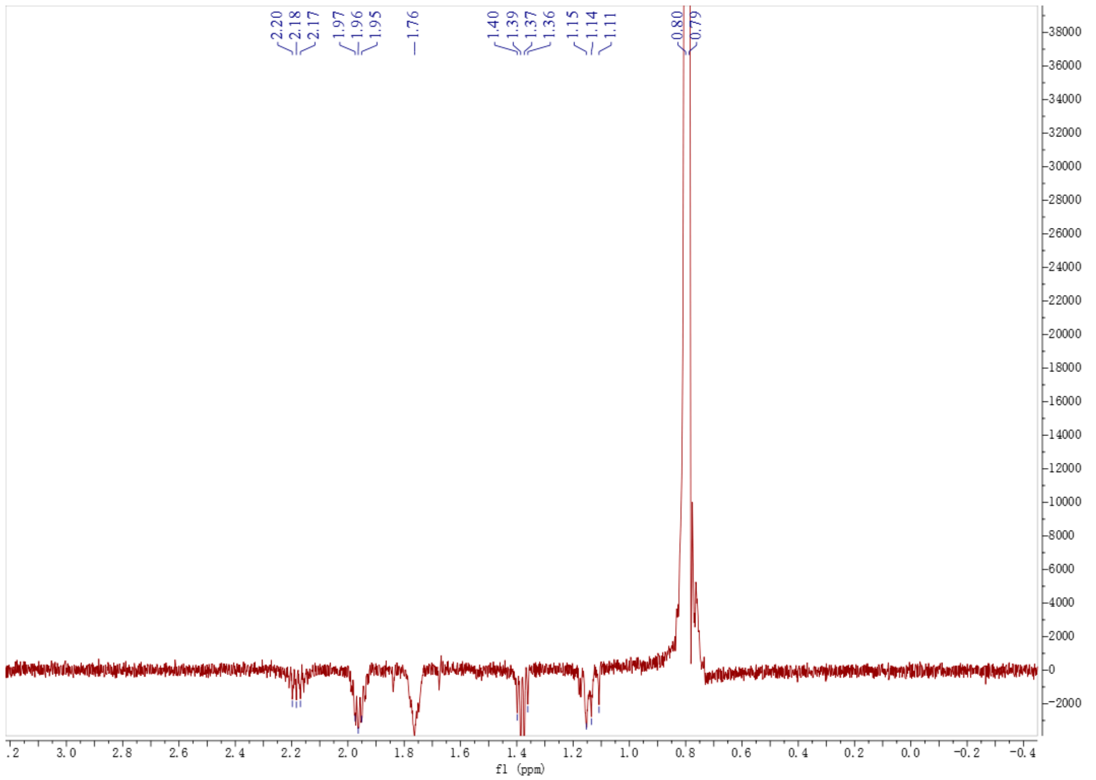

Fig. S47 [1]D -NOE (δ_H0.79) spectrum (600 MHz) of compound **4** in $CDCl_3$.

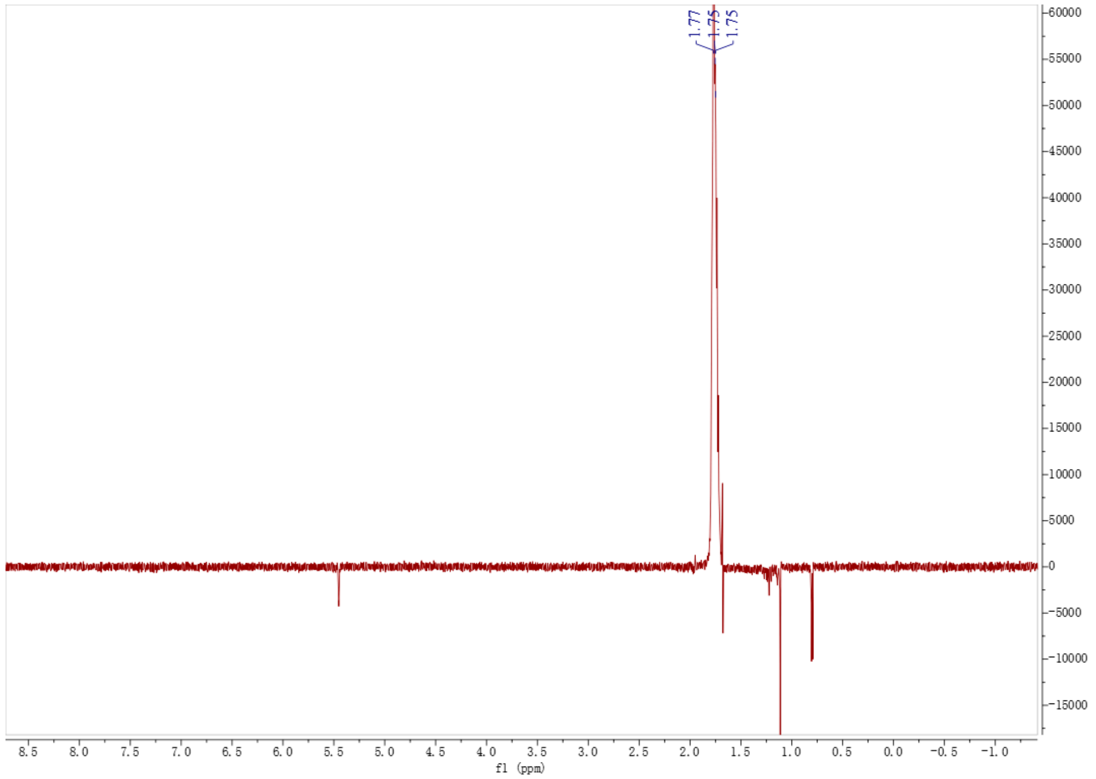

Fig. S48 [1]D -NOE (δ_H1.76) spectrum (600 MHz) of compound **4** in $CDCl_3$.

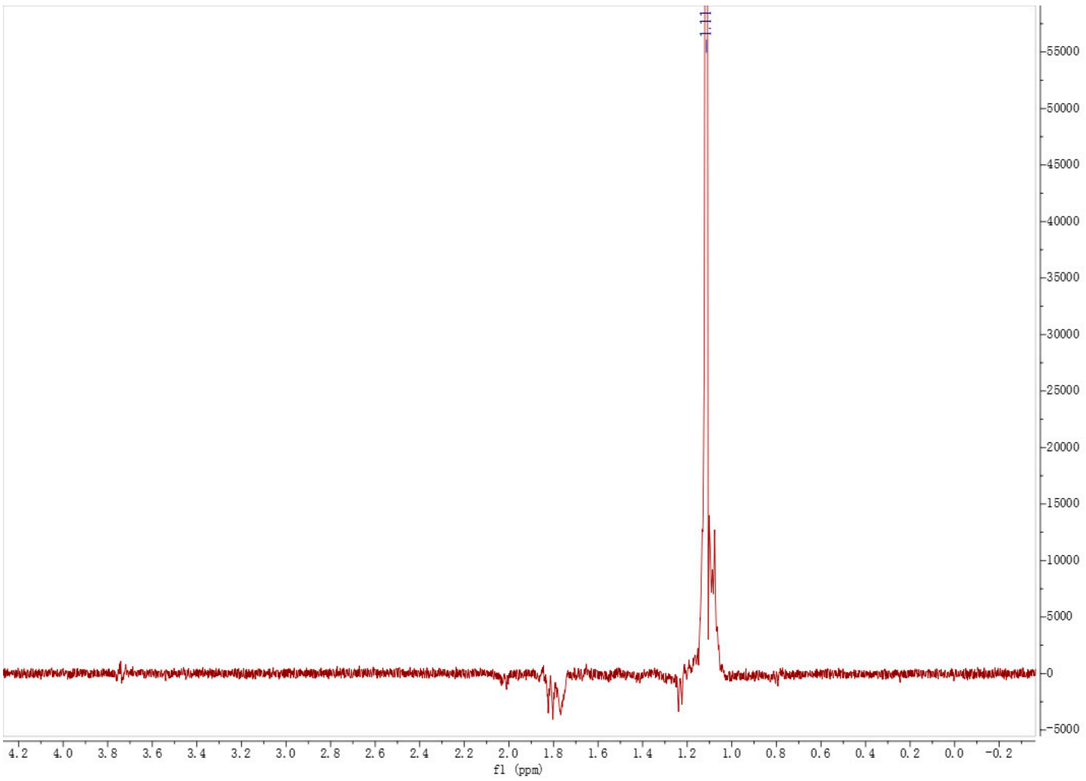

Fig. S49 [1]D -NOE (δ_H1.10) spectrum (600 MHz) of compound **4** in $CDCl_3$.

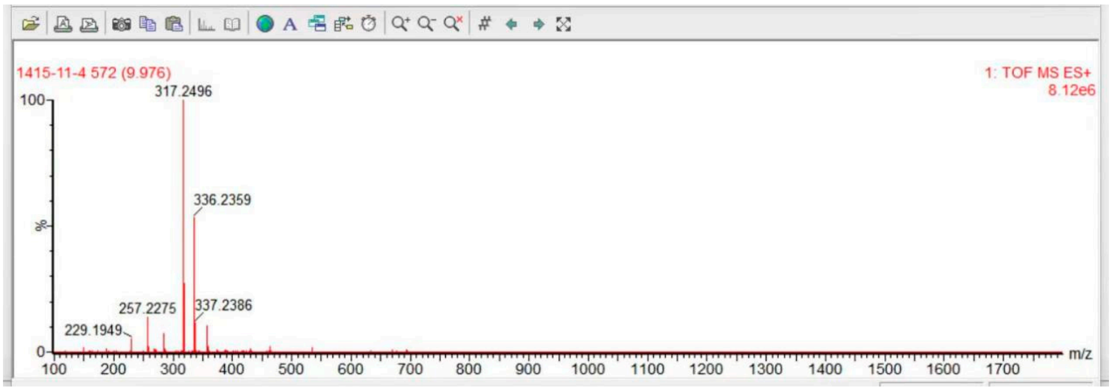

Fig. S50 HR-ESIMS spectrum of compound **4**.

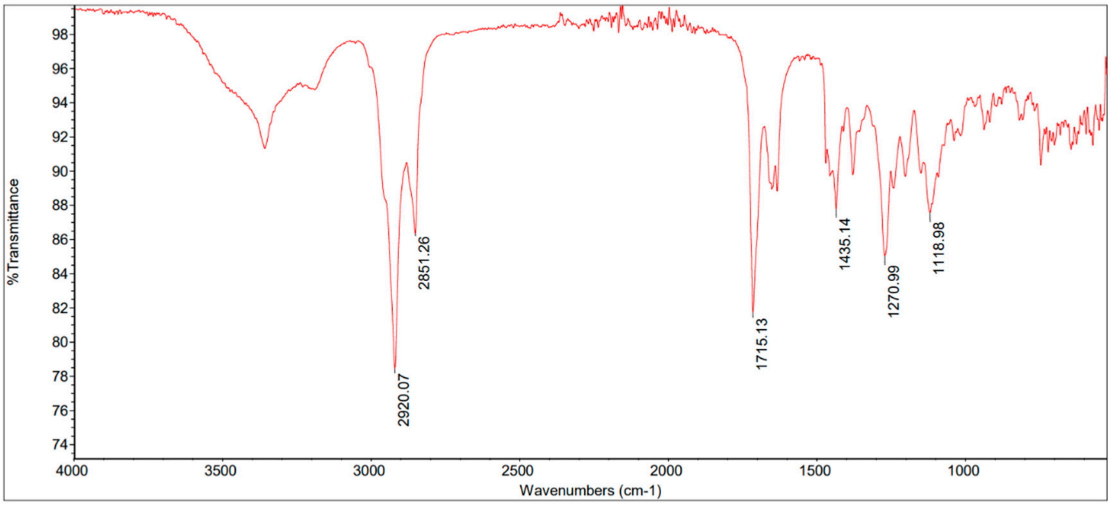

Fig. S51 Infrared spectrum of compound **4**.

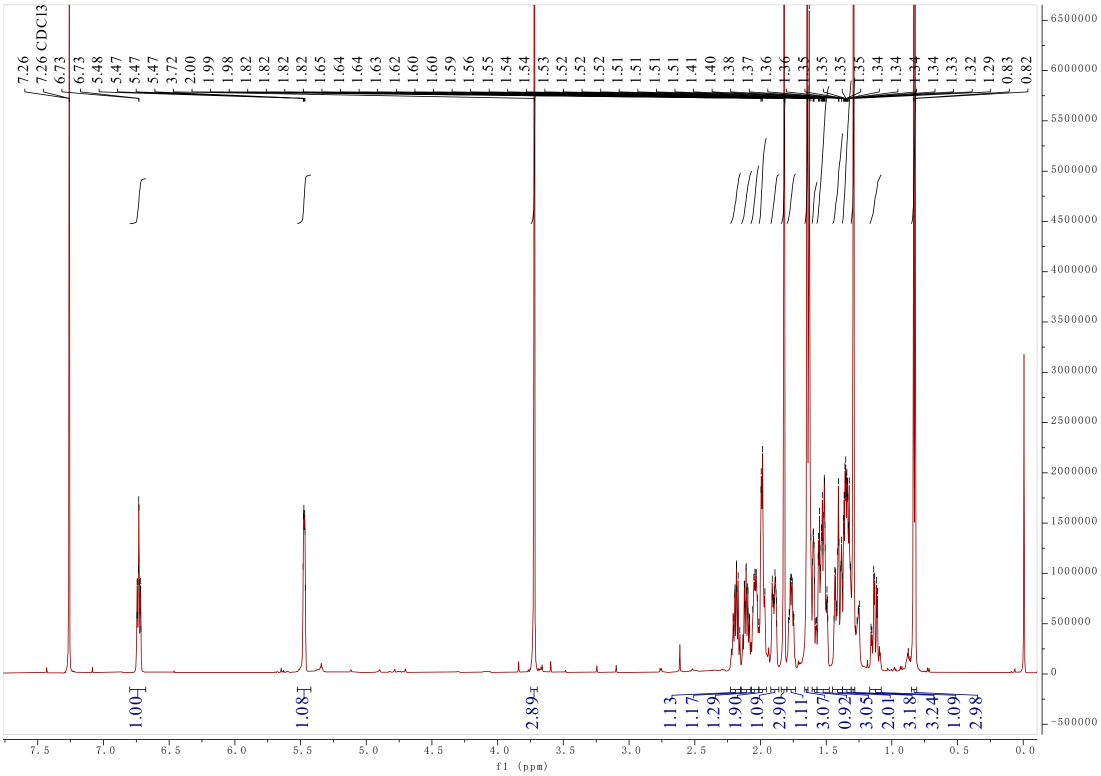

Fig. S52 ^{1}H NMR spectrum (600 MHz) of compound **5** in $CDCl_3$.

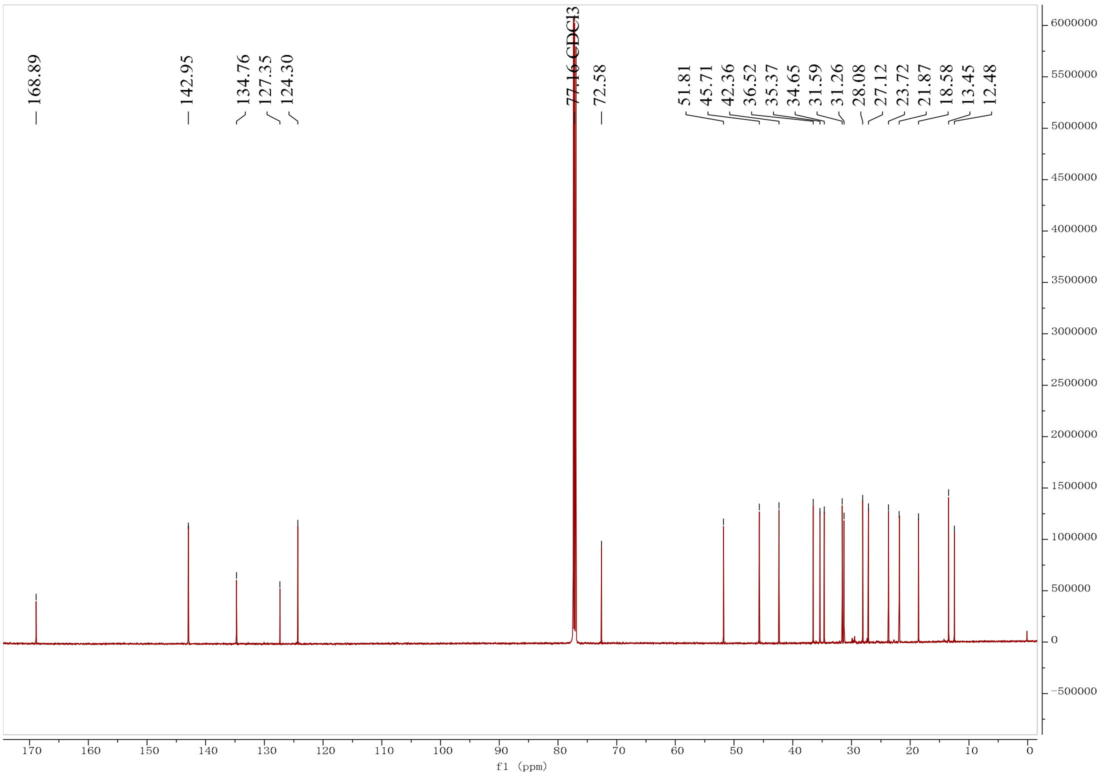

Fig. S53 ^{13}C NMR spectrum (150 MHz) of compound **5** in $CDCl_3$.

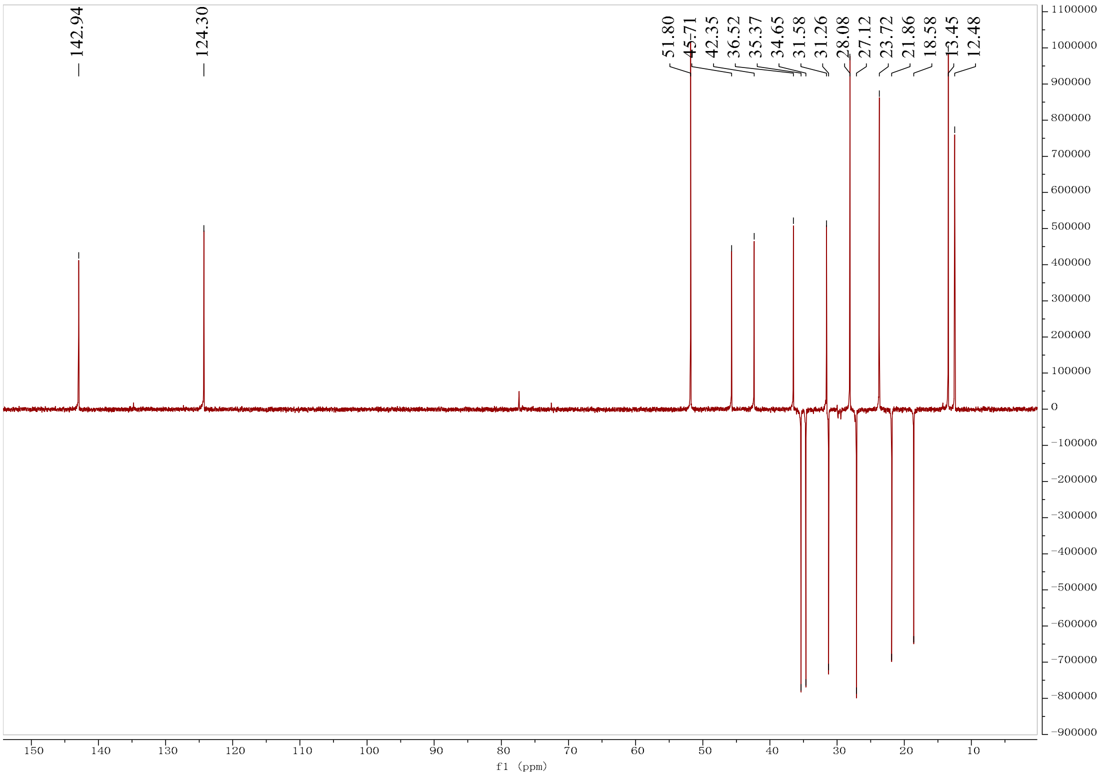

Fig. S54 DEPT spectrum (150 MHz) of compound **5** in $CDCl_3$.

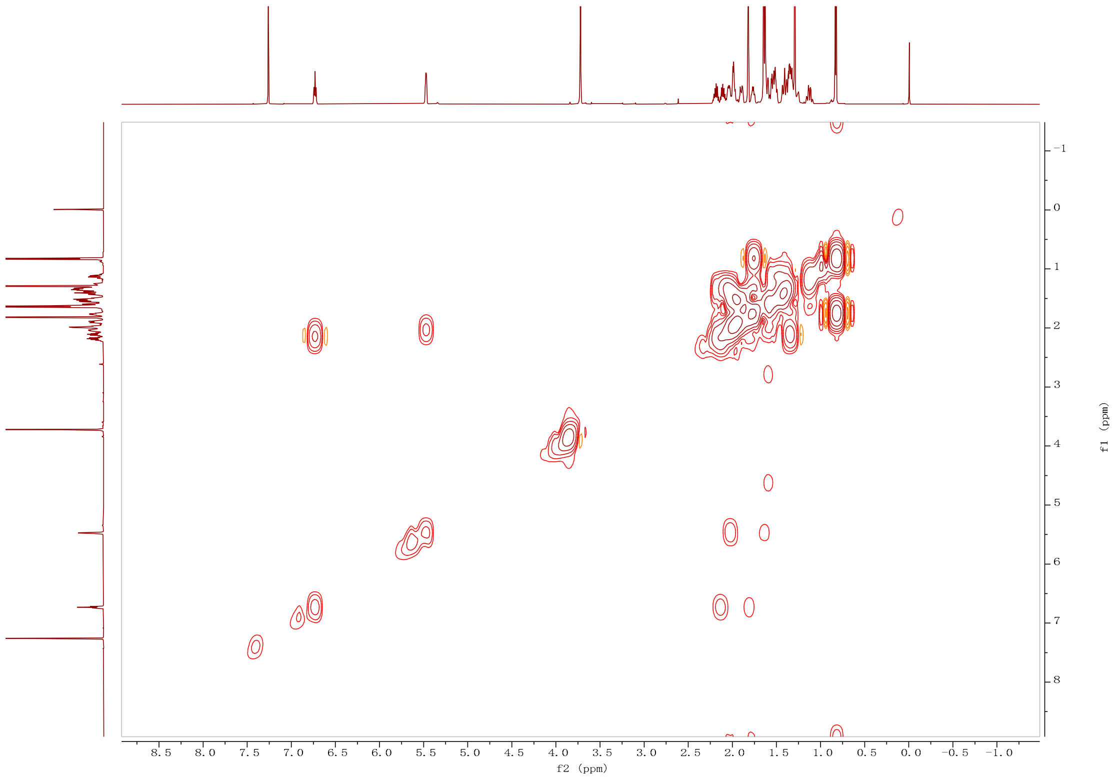

Fig. S55 ^{1}H-^{1}H COSY spectrum (600 MHz) of compound **5** in $CDCl_3$.

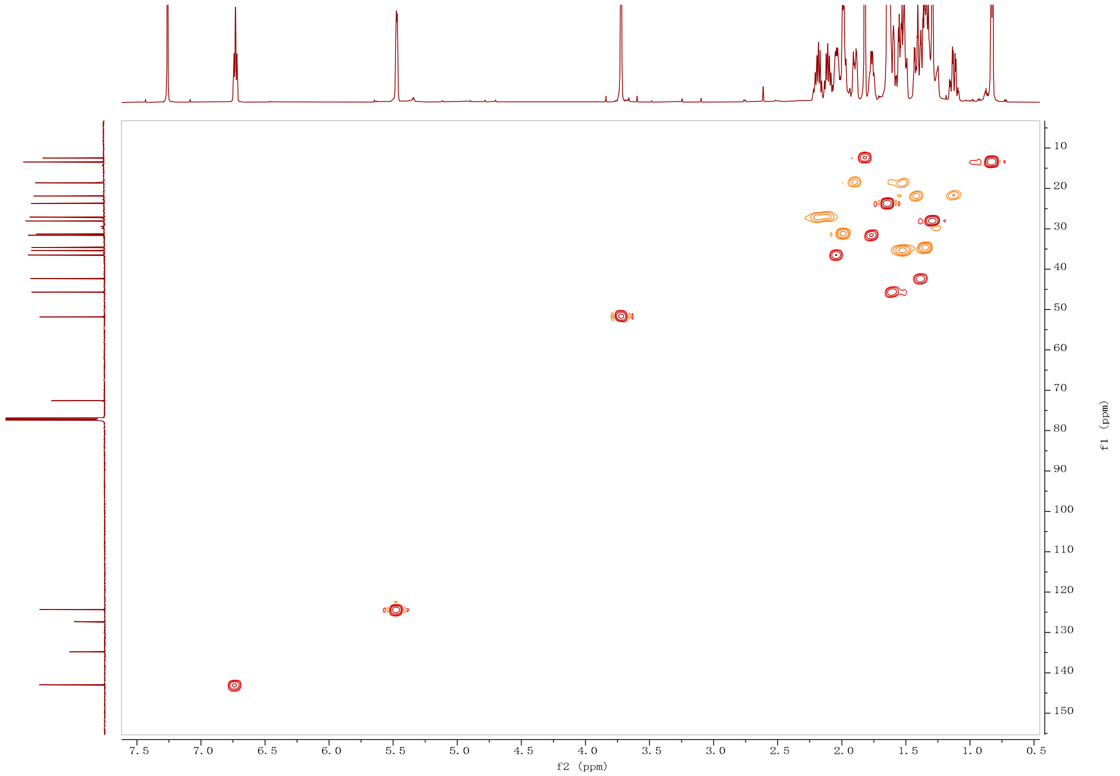

Fig. S56 HSQC spectrum (600 MHz) of compound **5** in $CDCl_3$.

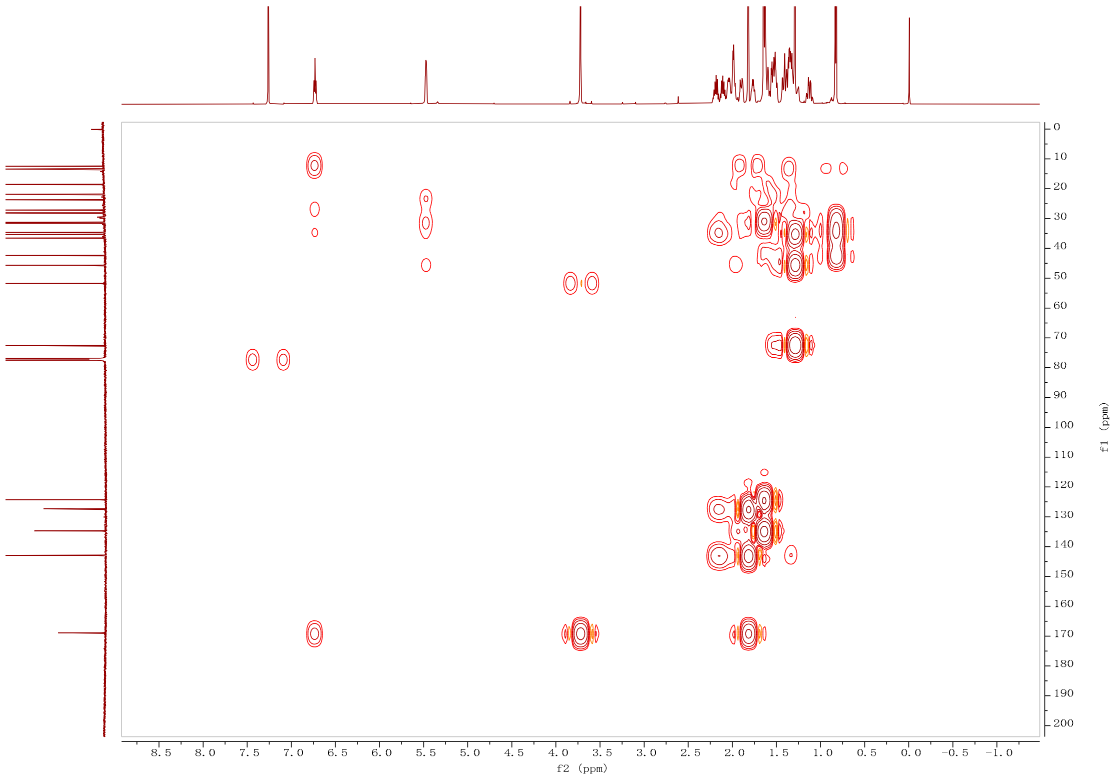

Fig. S57 HMBC spectrum (600 MHz) of compound **5** in $CDCl_3$.

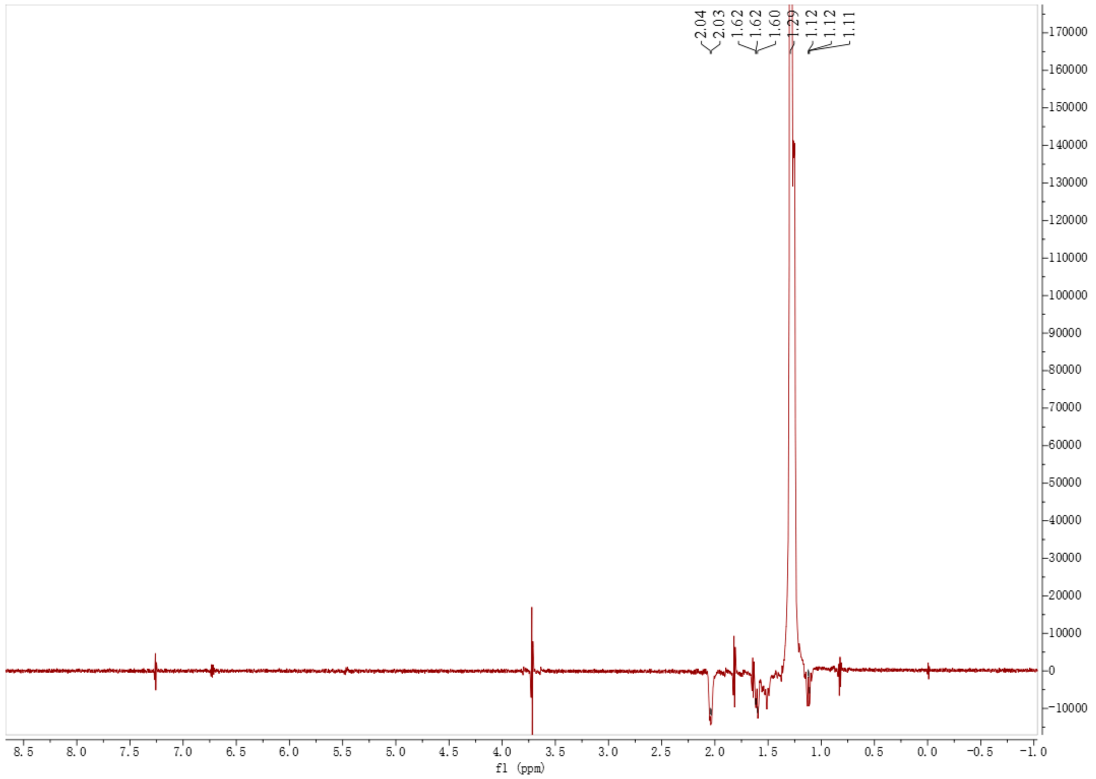

Fig. S58 [1]D -NOE (δ_H1.29) spectrum (600 MHz) of compound **5** in $CDCl_3$.

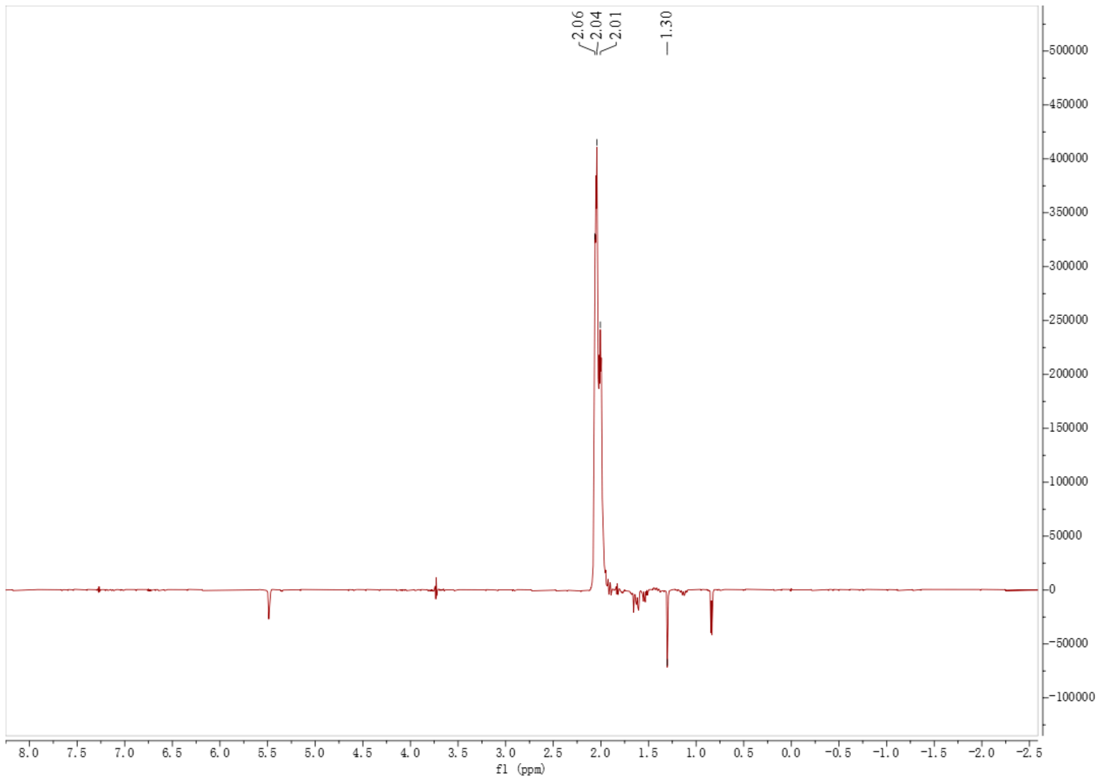

Fig. S59 [1]D -NOE (δ_H2.04) spectrum (600 MHz) of compound **5** in $CDCl_3$.

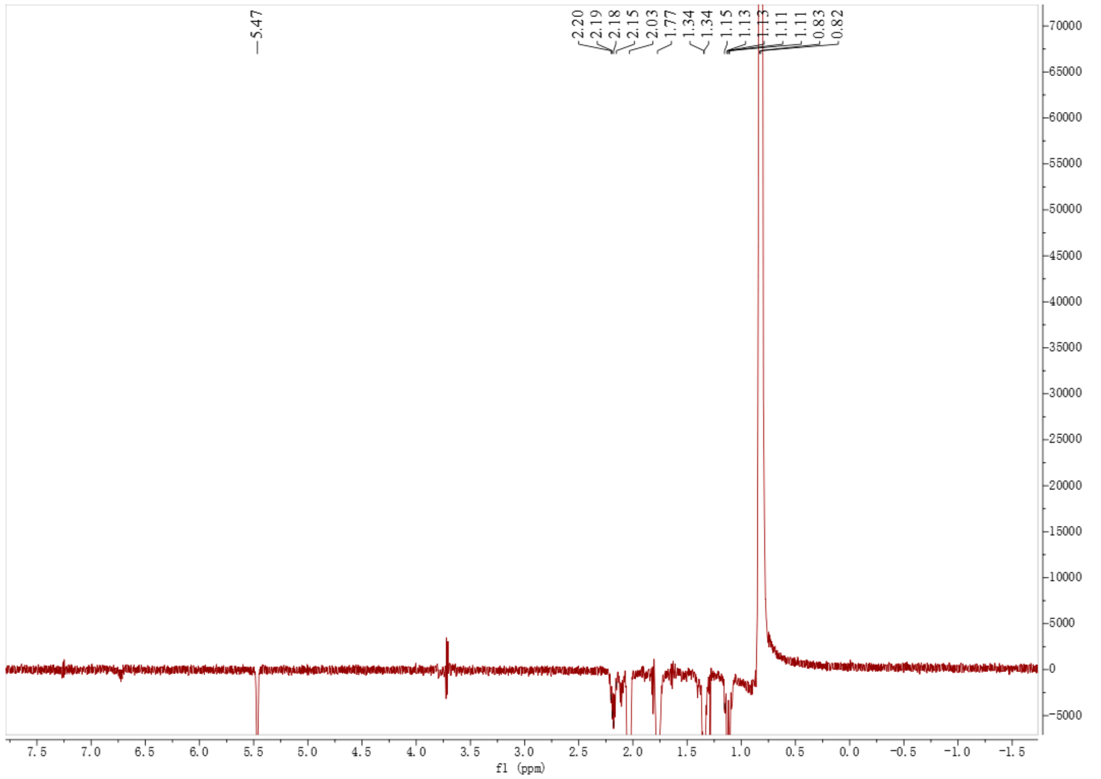

Fig. S60 [1]D -NOE (δ_H0.83) spectrum (600 MHz) of compound **5** in $CDCl_3$.

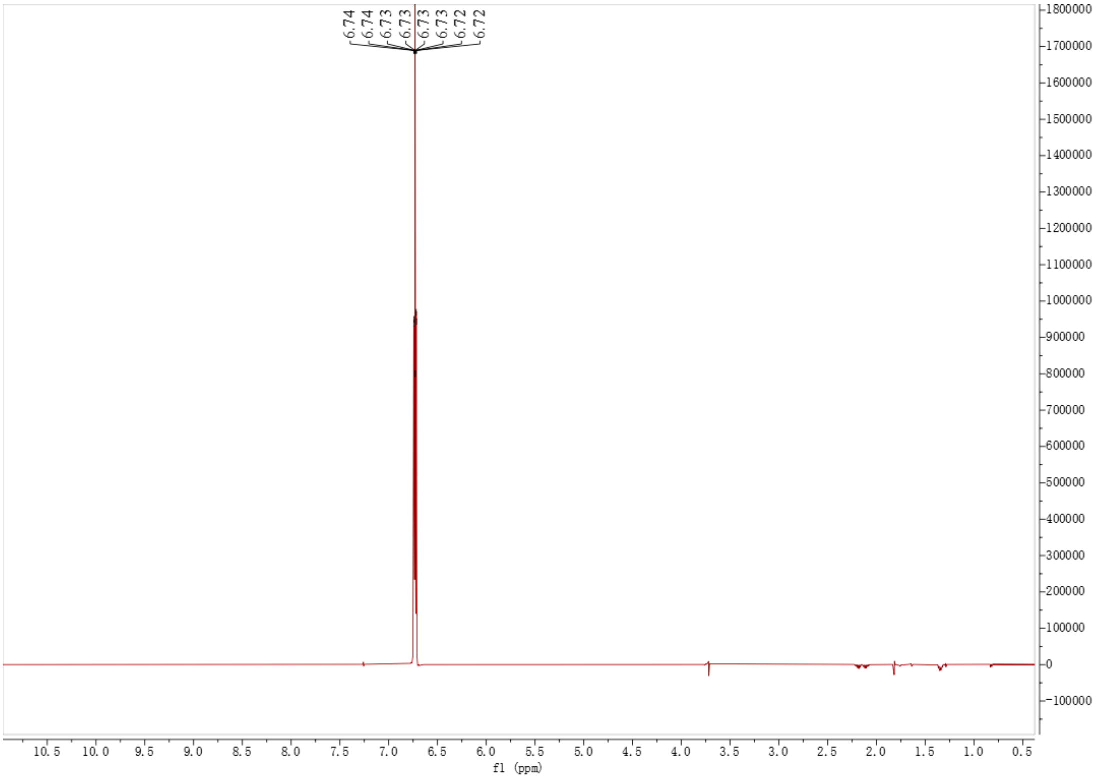

Fig. S61 [1]D -NOE (δ_H6.73) spectrum (600 MHz) of compound **5** in $CDCl_3$.

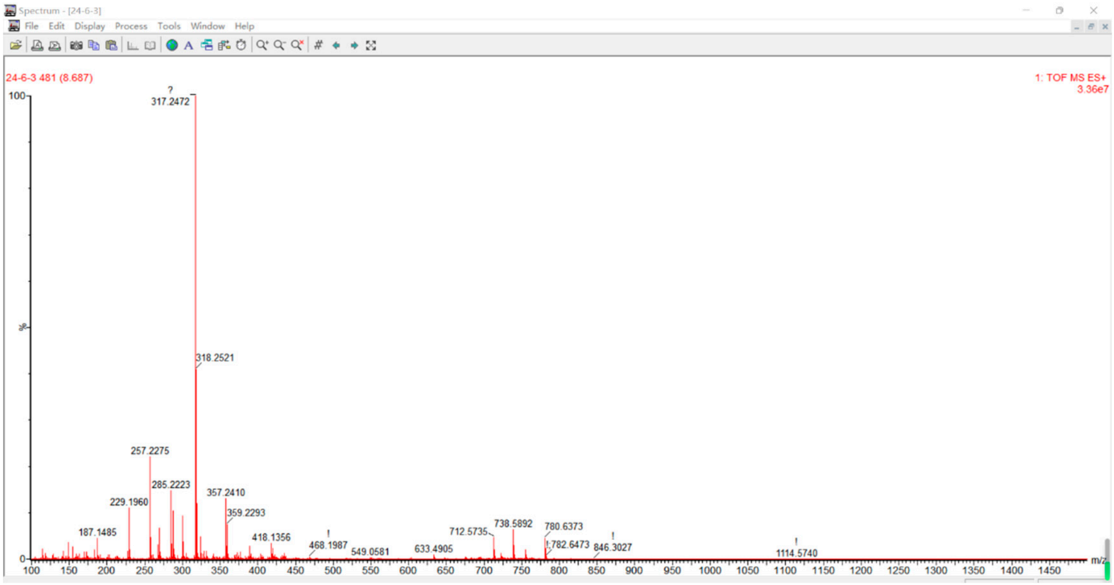

Fig. S62 HR-ESIMS spectrum of compound **5**.

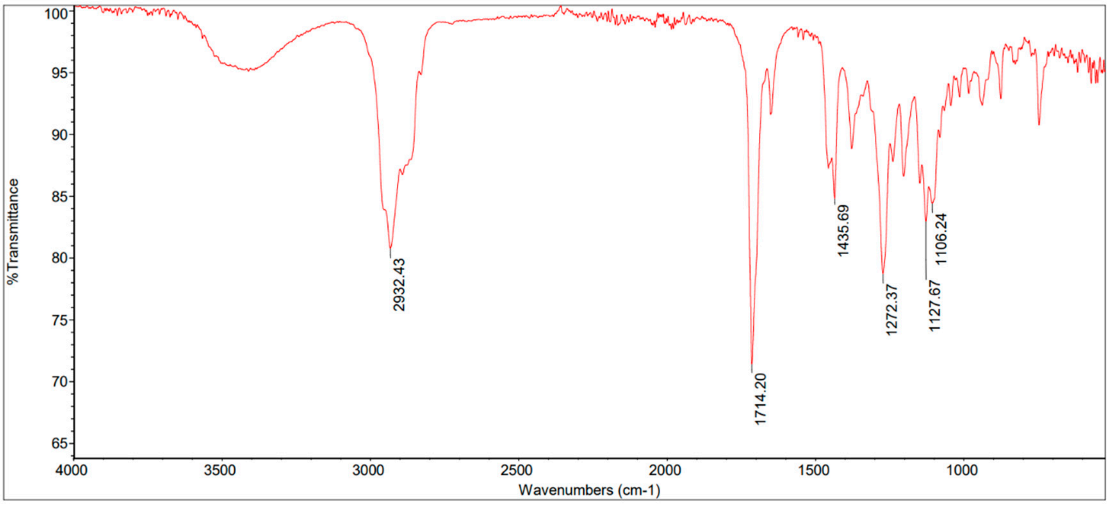

Fig. S63 Infrared spectrum of compound **5**.

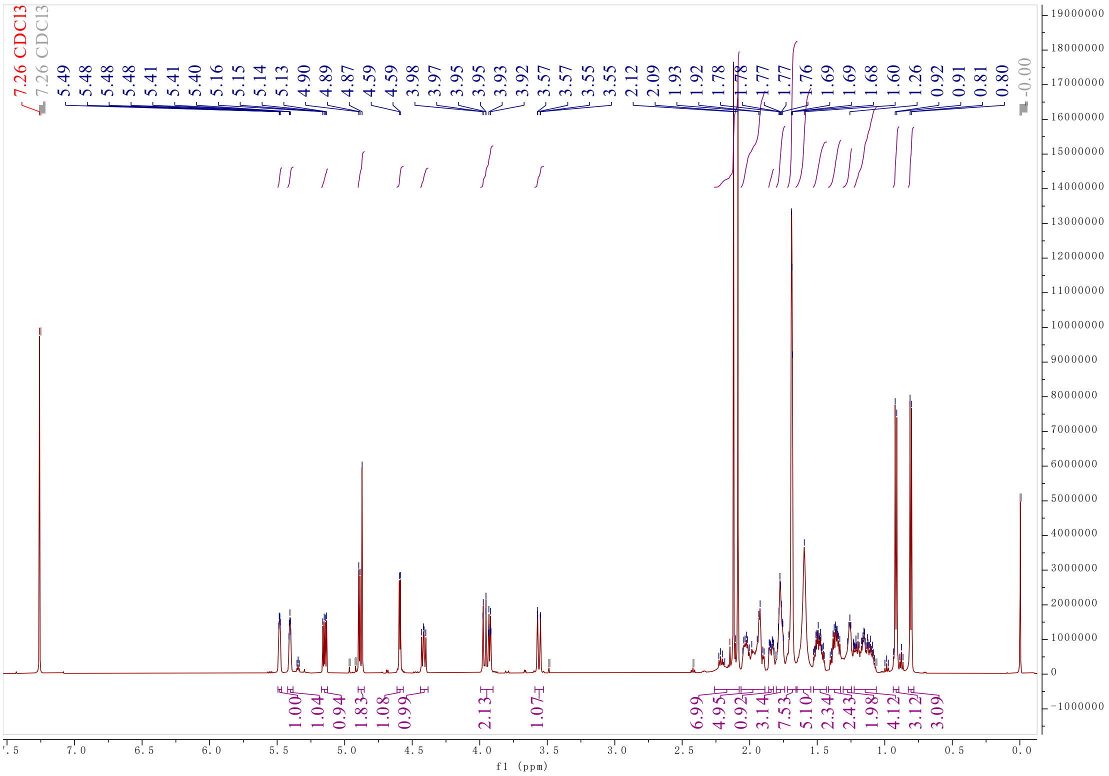

Fig. S64 ^{1}H NMR spectrum (600 MHz) of compound **6** in $CDCl_3$.

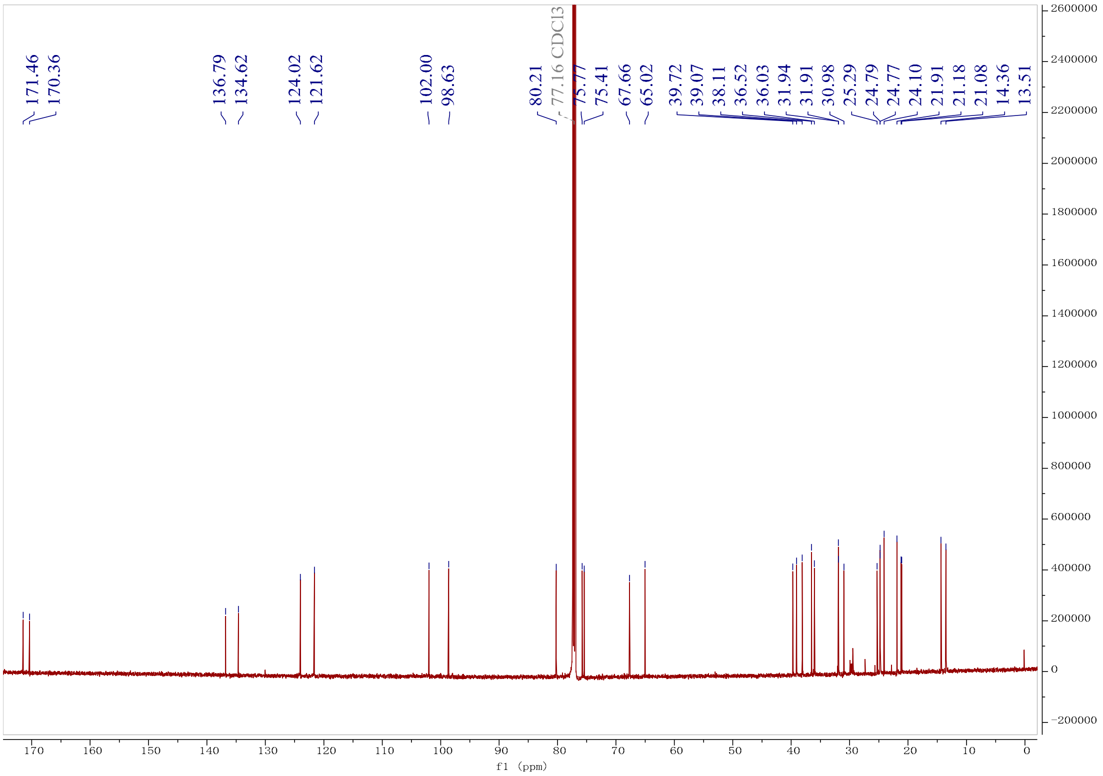

Fig. S65 ^{13}C NMR spectrum (150 MHz) of compound **6** in $CDCl_3$.

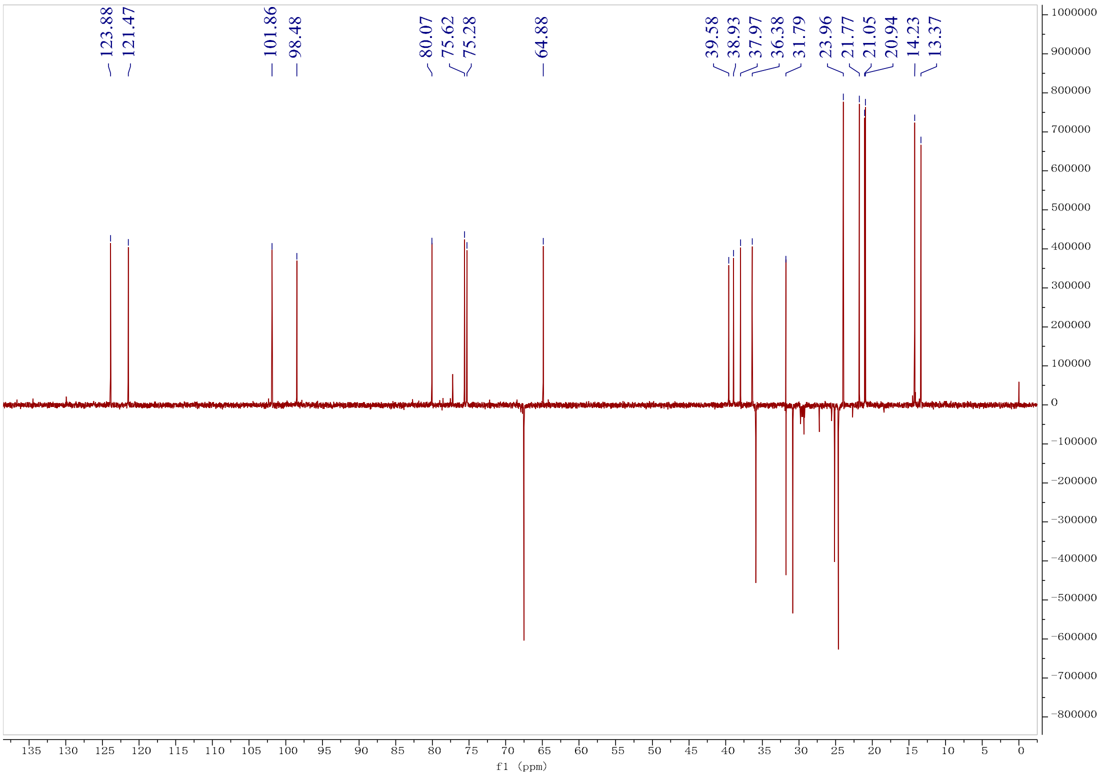

Fig. S66 DEPT spectrum (150 MHz) of compound **6** in $CDCl_3$.

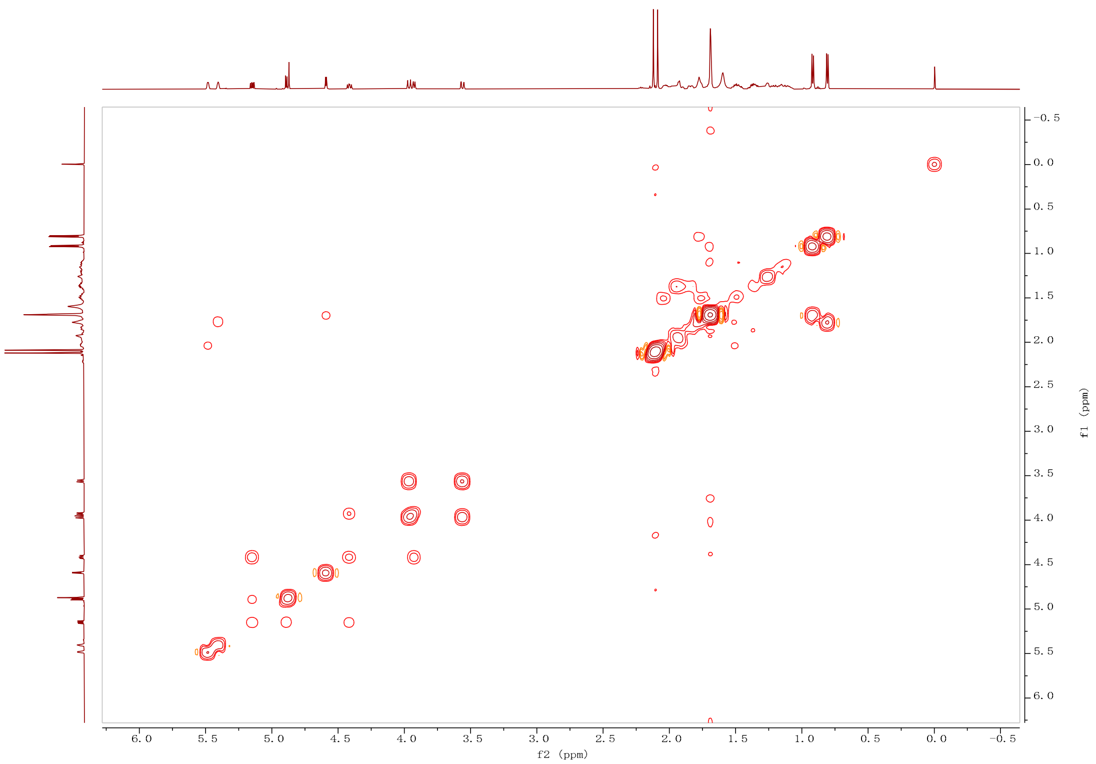

Fig. S67 ^{1}H-^{1}H COSY spectrum (600 MHz) of compound **6** in $CDCl_3$.

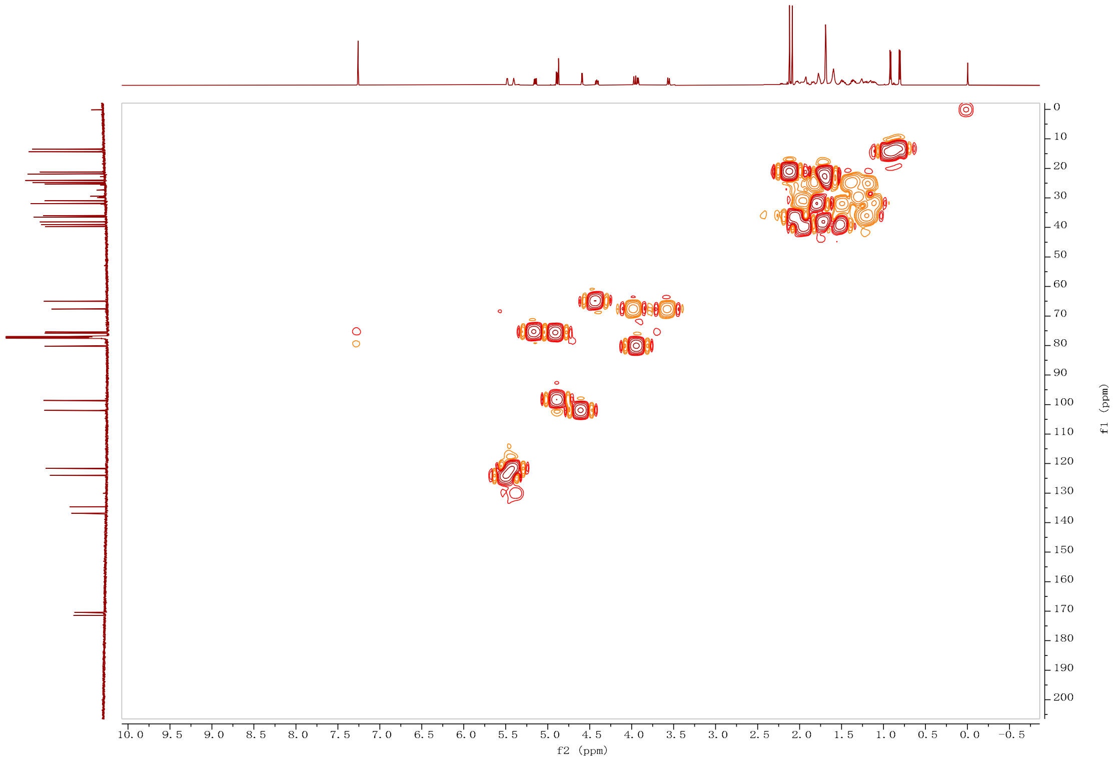

Fig. S68 HSQC spectrum (600 MHz) of compound **6** in $CDCl_3$.

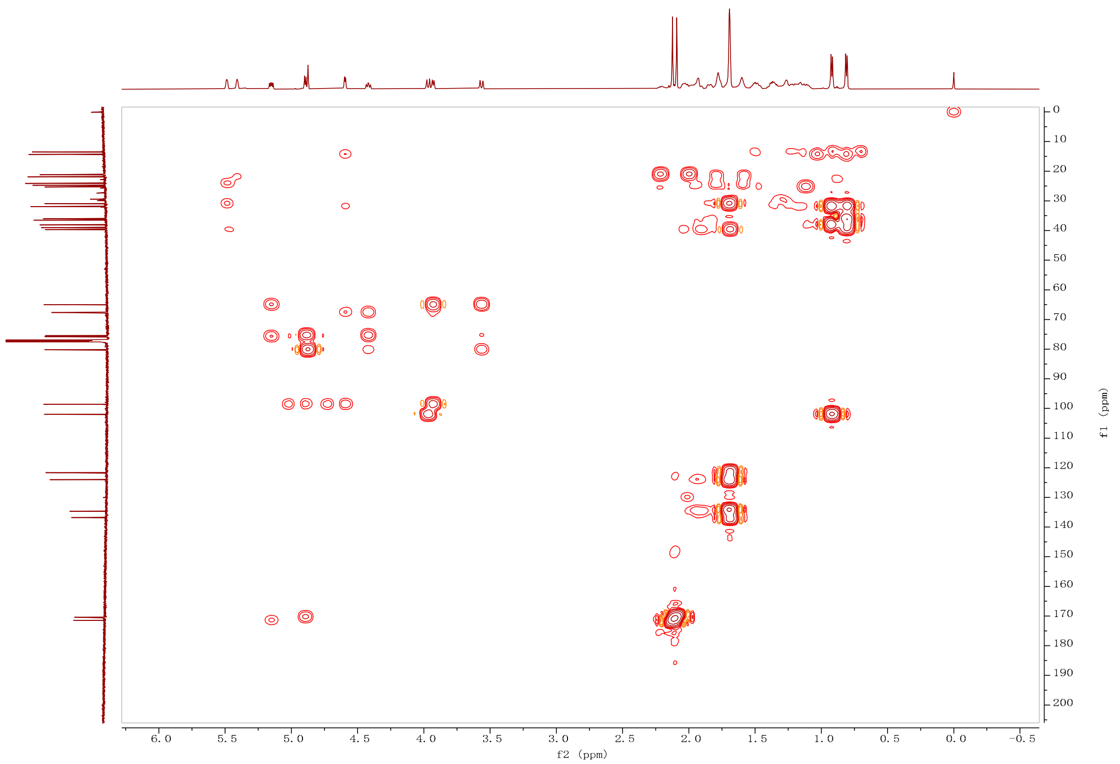

Fig. S69 HMBC spectrum (600 MHz) of compound **6** in $CDCl_3$.

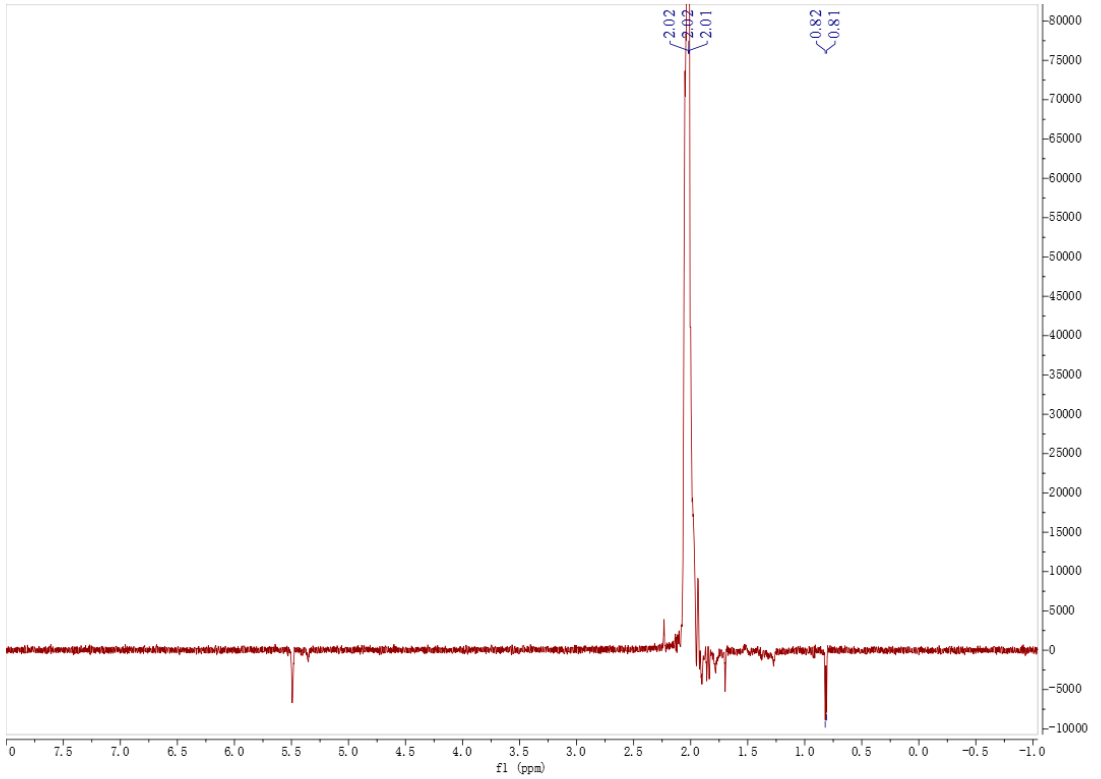

Fig. S70 ^{1}D -NOE (δ_H2.03) spectrum (600 MHz) of compound **6** in $CDCl_3$.

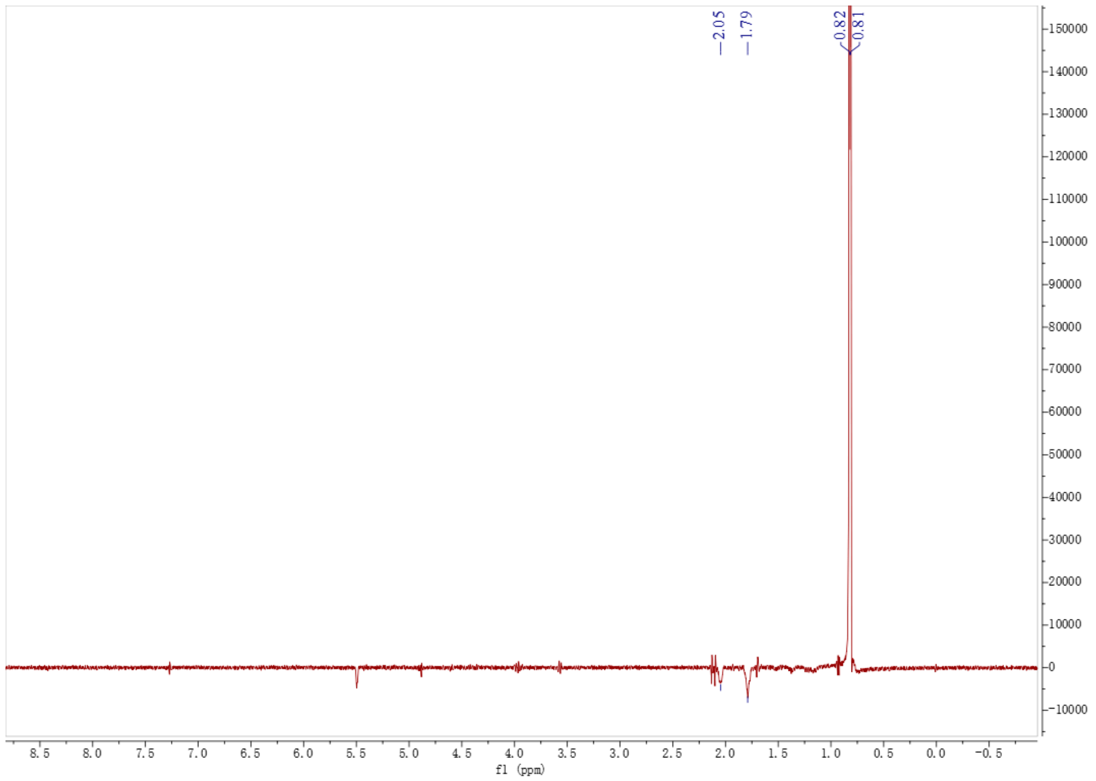

Fig. S71 ^{1}D -NOE (δ_H0.81) spectrum (600 MHz) of compound **6** in $CDCl_3$.

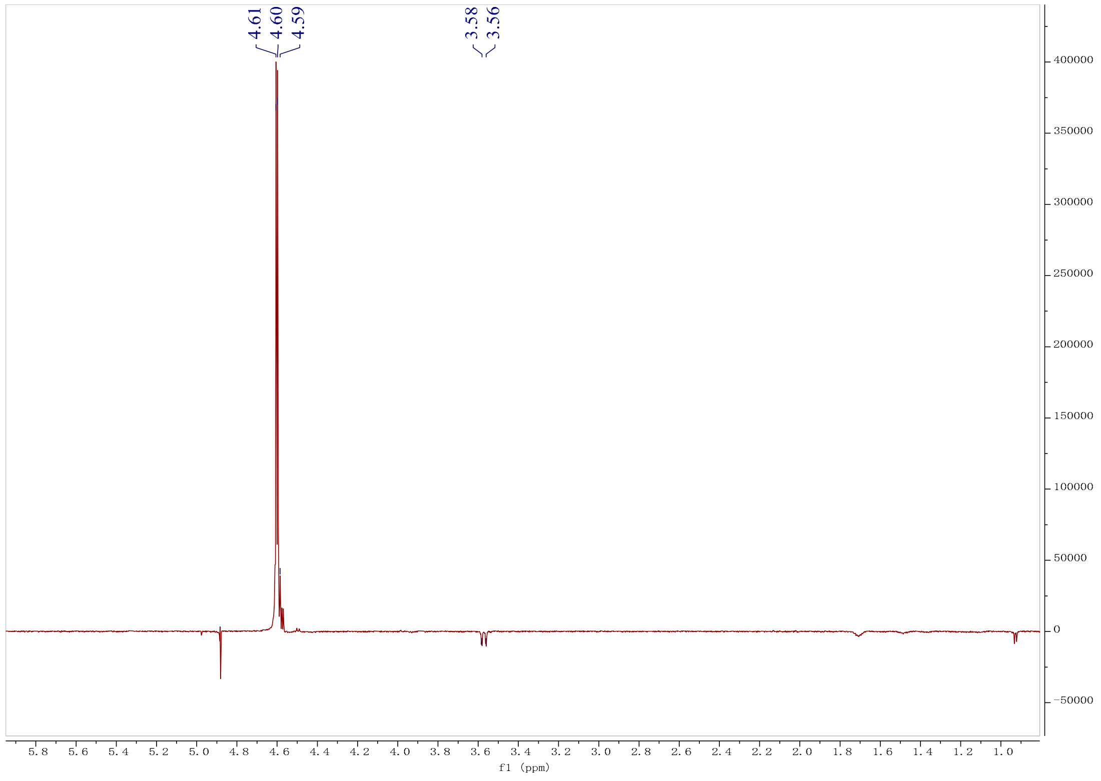

Fig. S72 ^{1}D -NOE (δ_H4.60) spectrum (600 MHz) of compound **6** in $CDCl_3$.

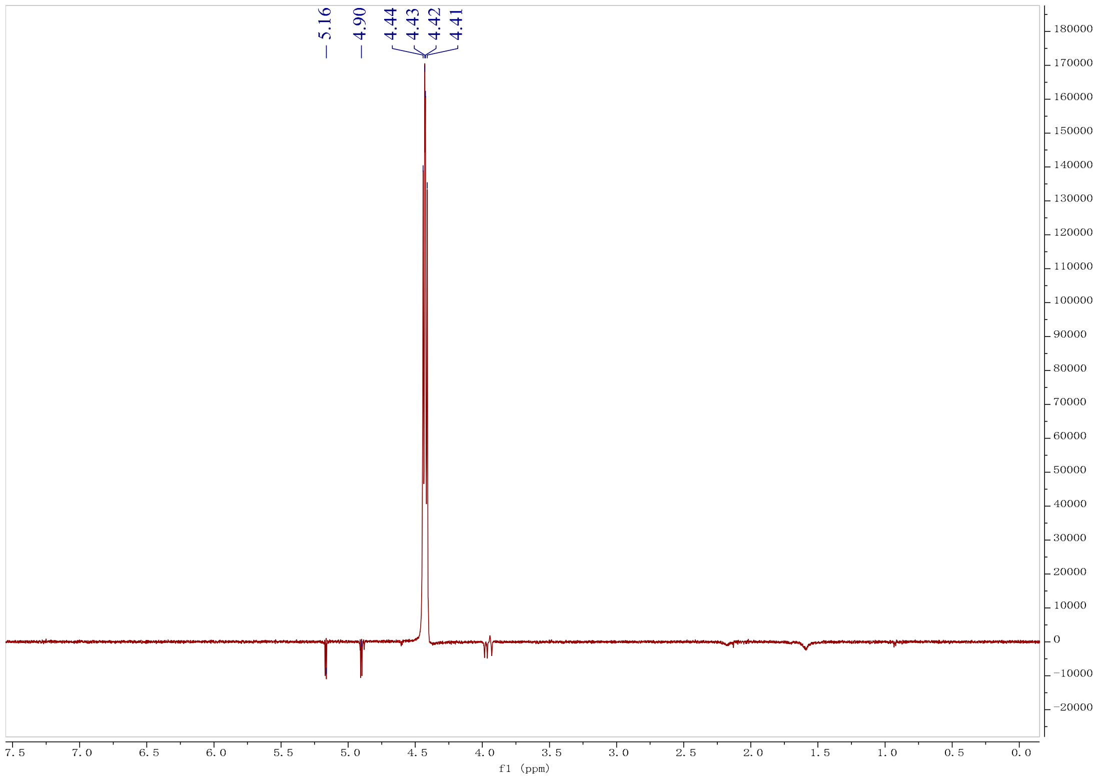

Fig. S73 [1]D -NOE (δ_H4.41) spectrum (600 MHz) of compound **6** in $CDCl_3$.

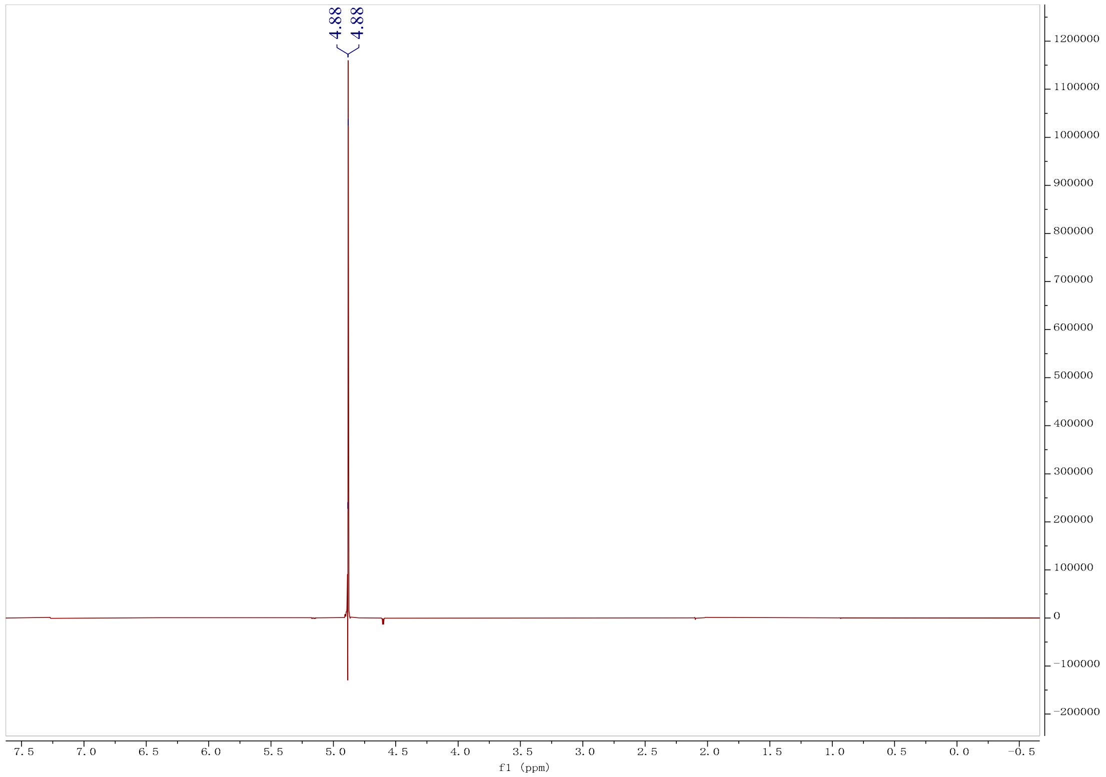

Fig. S74 [1]D -NOE (δ_H4.88) spectrum (600 MHz) of compound **6** in $CDCl_3$.

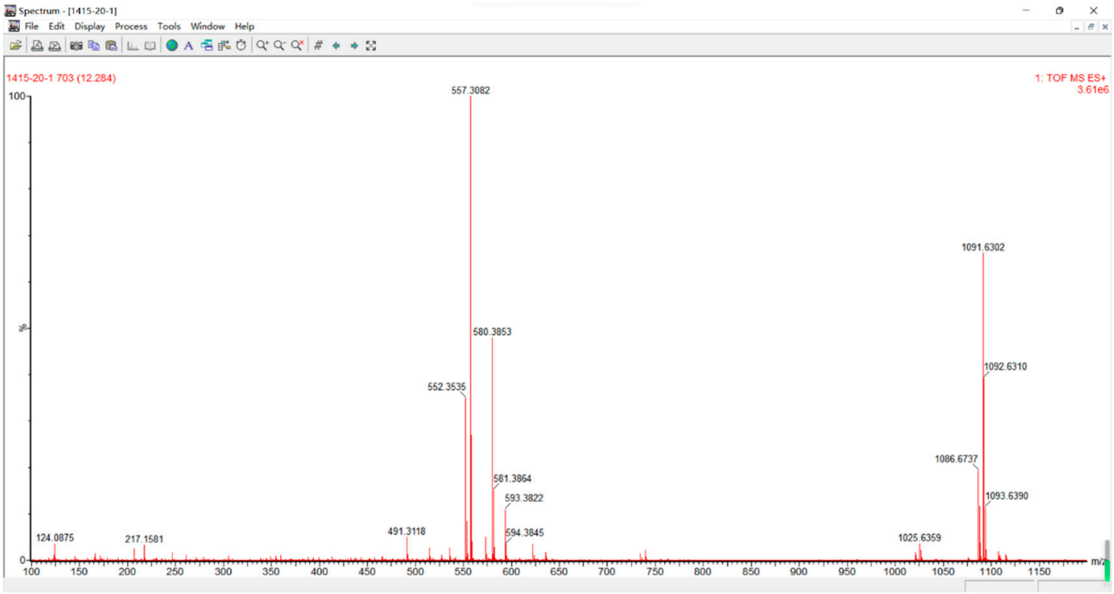

Fig. S75 HR-ESIMS spectrum of compound **6**.

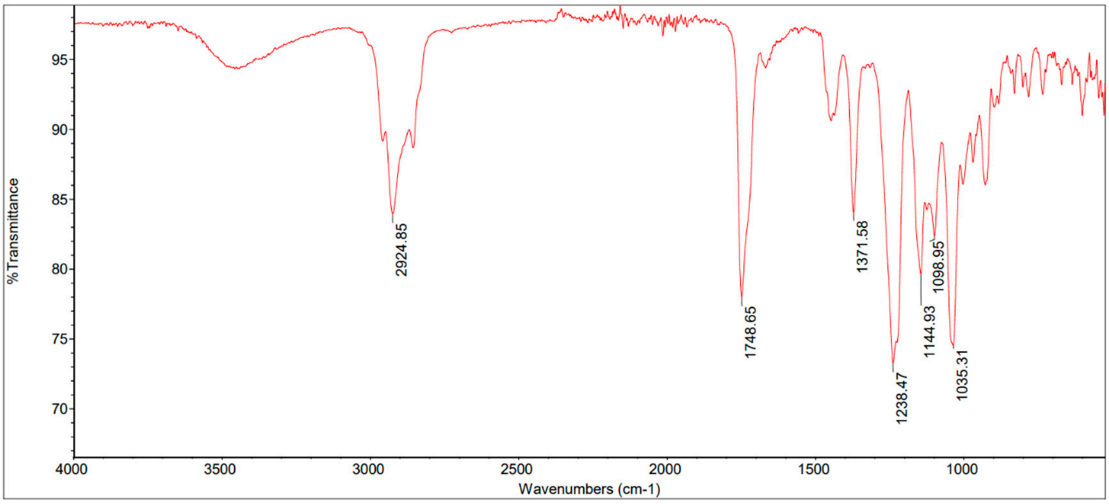

Fig. S76 Infrared spectrum of compound **6**.

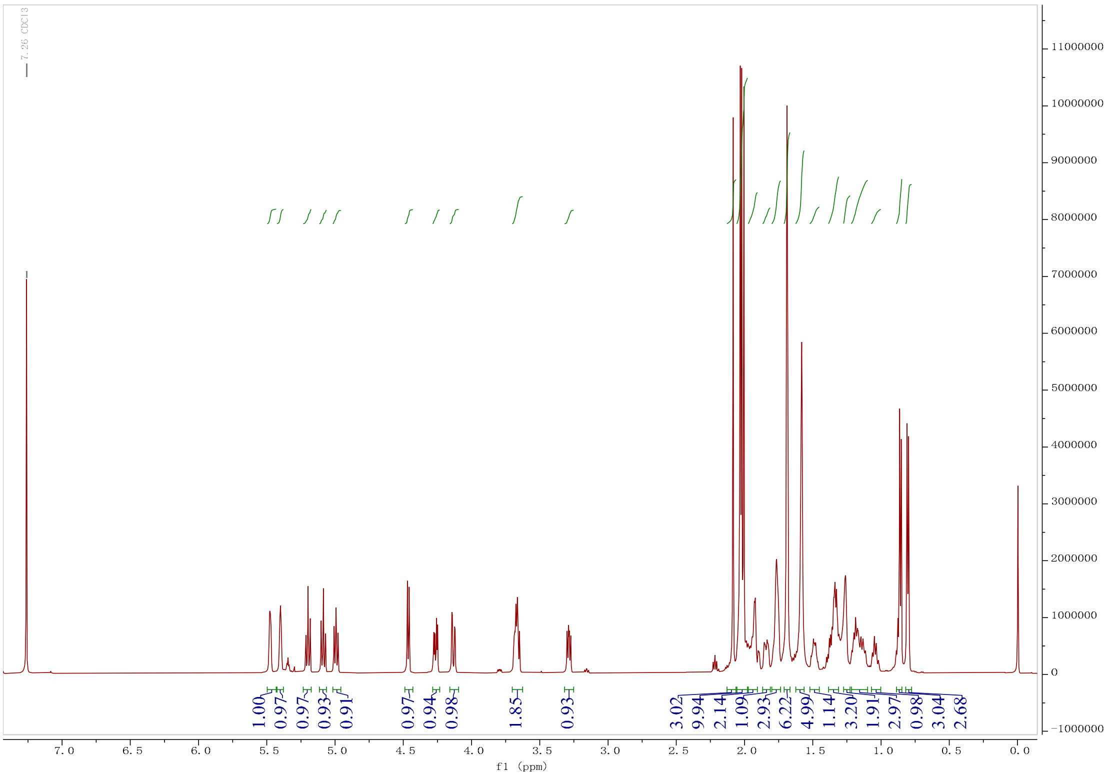

Fig. S77 ^{1}H NMR spectrum (600 MHz) of compound **7** in $CDCl_3$.

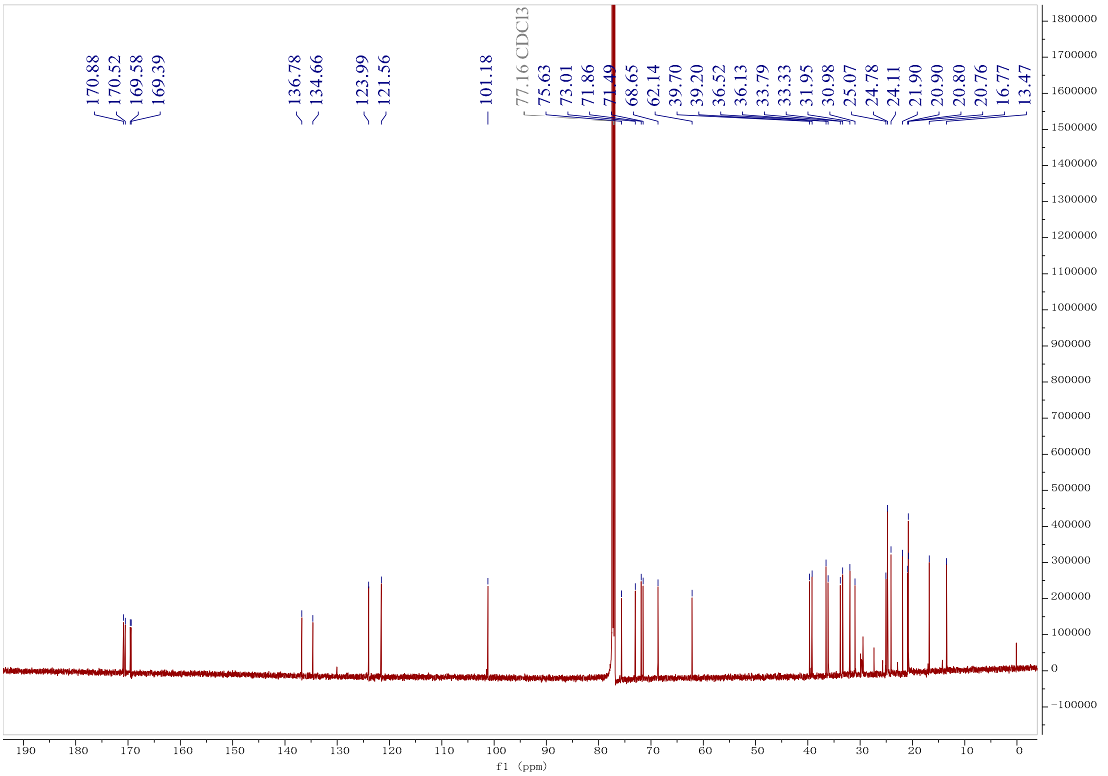

Fig. S78 ^{13}C NMR spectrum (150 MHz) of compound **7** in $CDCl_3$.

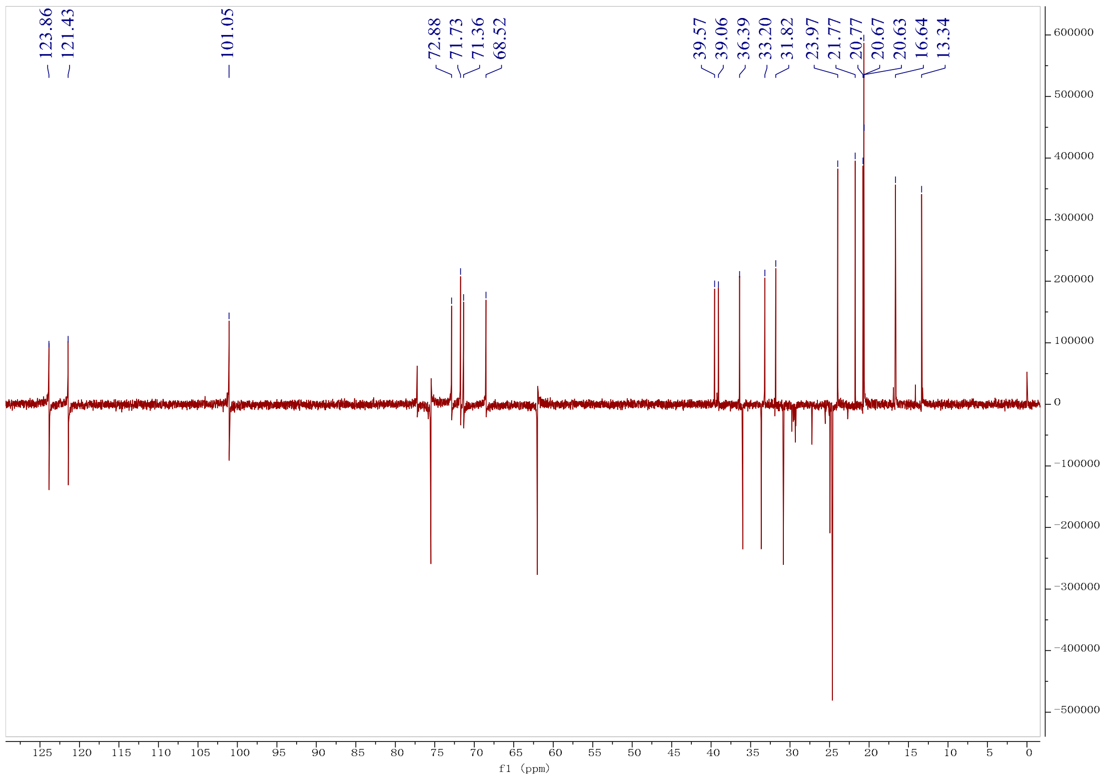

Fig. S79 DEPT spectrum (150 MHz) of compound **7** in $CDCl_3$.

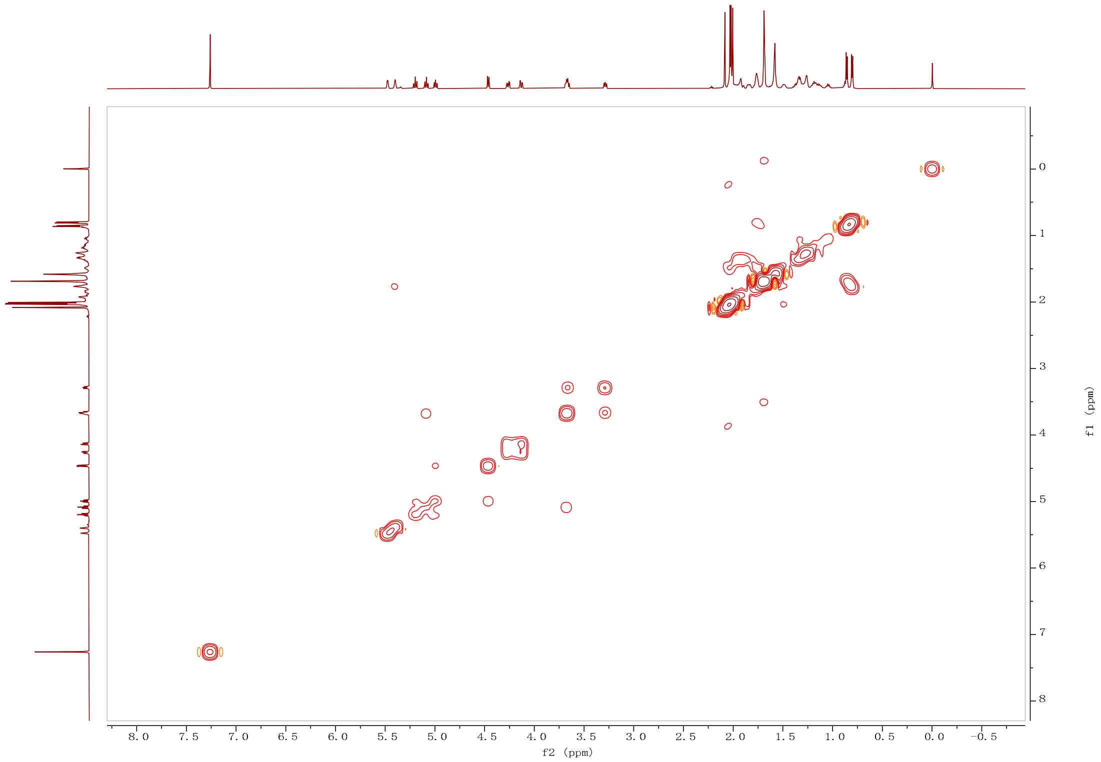

Fig. S80 ^{1}H-^{1}H COSY spectrum (600 MHz) of compound **7** in $CDCl_3$.

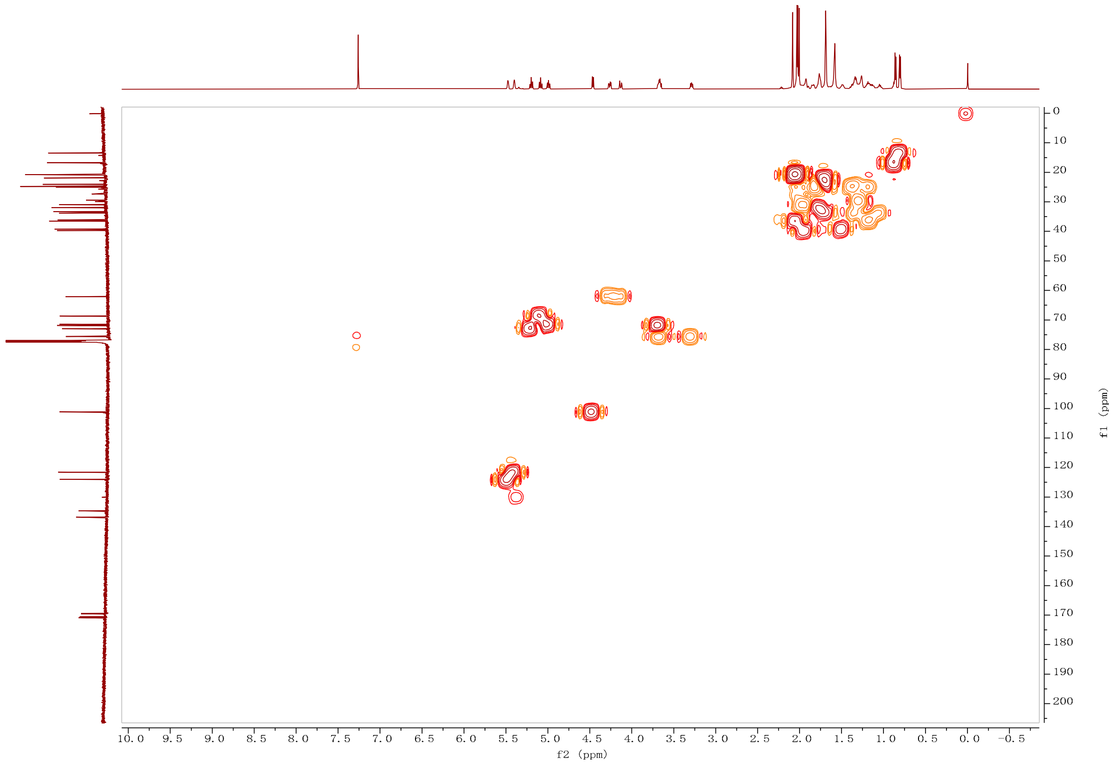

Fig. S81 HSQC spectrum (600 MHz) of compound **7** in $CDCl_3$.

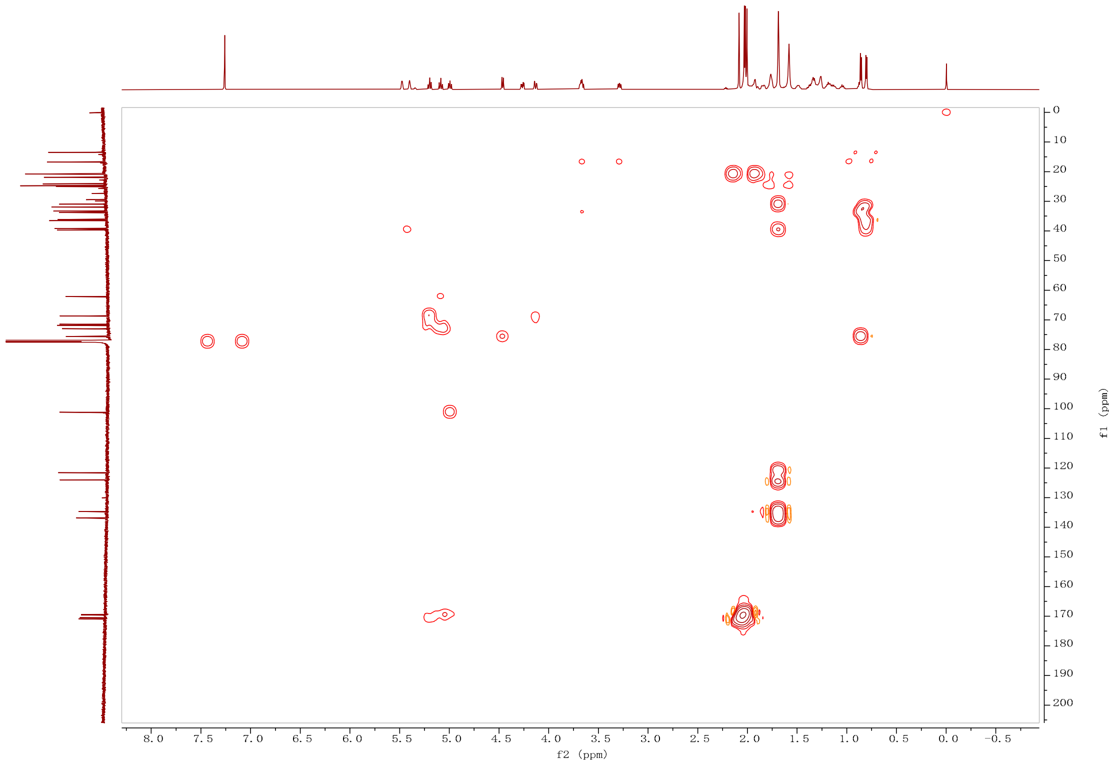

Fig. S82 HMBC spectrum (600 MHz) of compound **7** in $CDCl_3$.

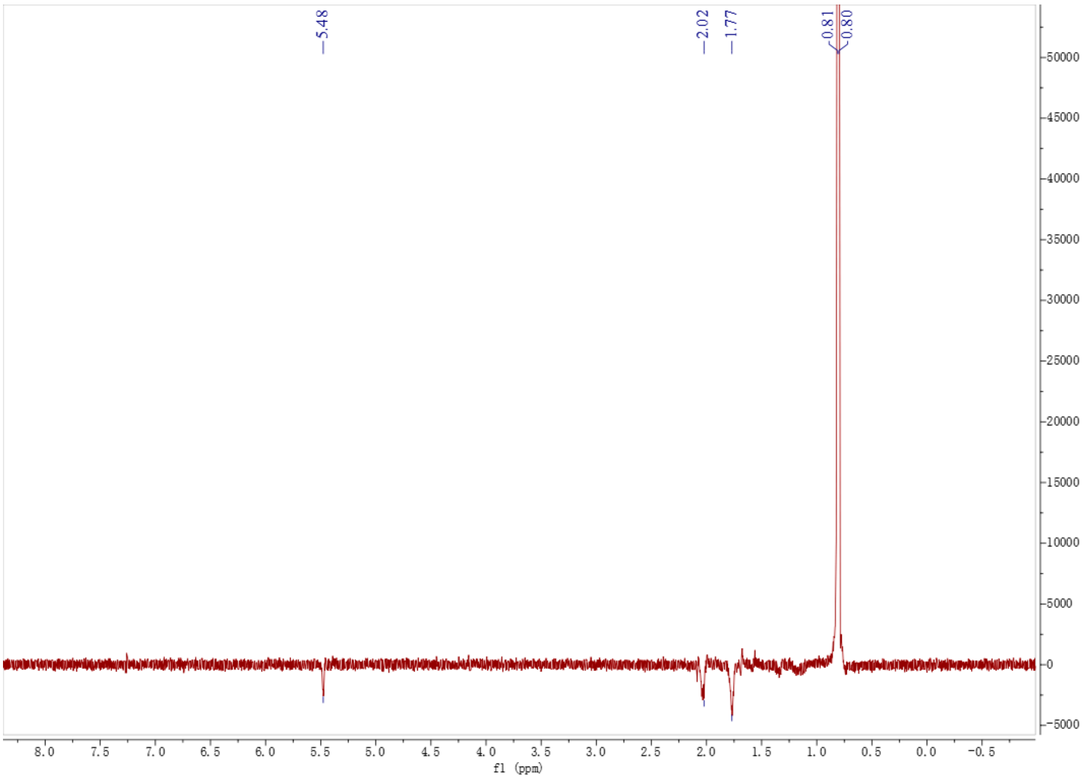

Fig. S83 [1]D -NOE (δ_H0.80) spectrum (600 MHz) of compound **7** in $CDCl_3$.

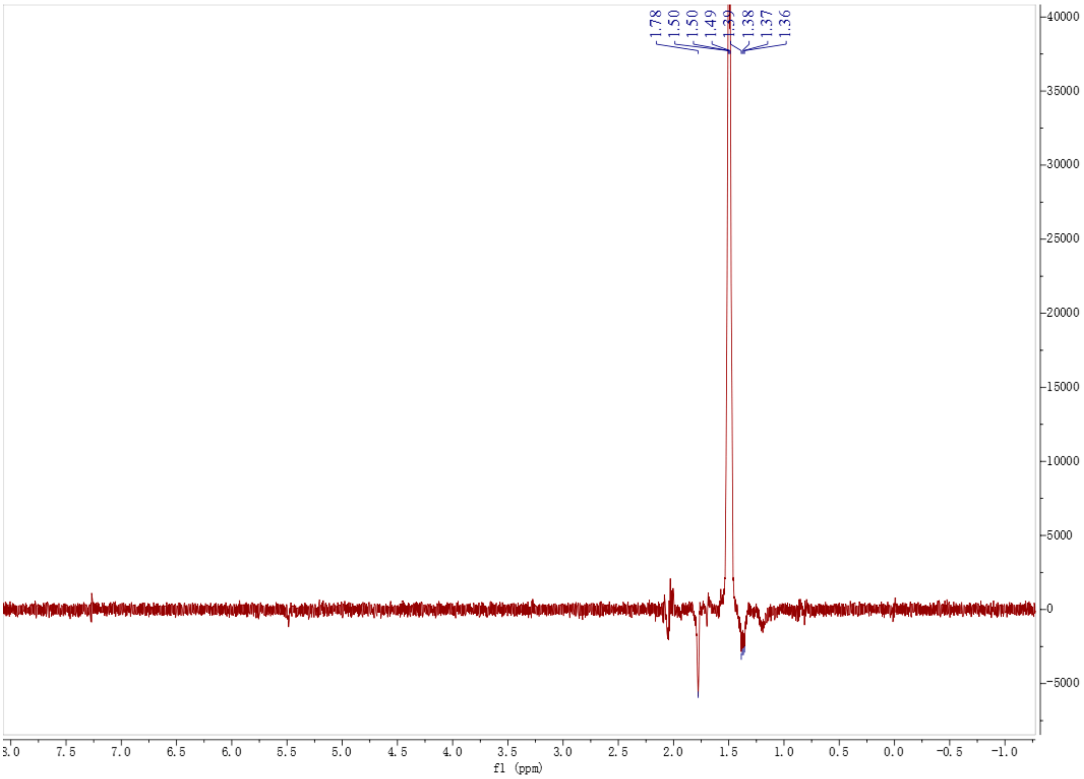

Fig. S84 [1]D -NOE (δ_H1.49) spectrum (600 MHz) of compound **7** in $CDCl_3$.

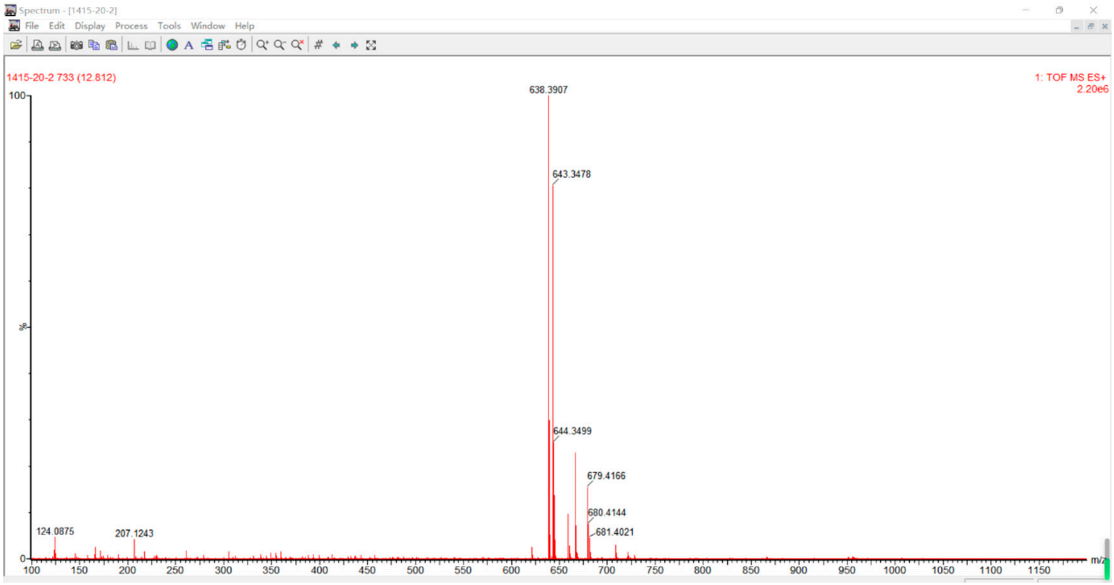

Fig. S85 HR-ESIMS spectrum of compound **7**.

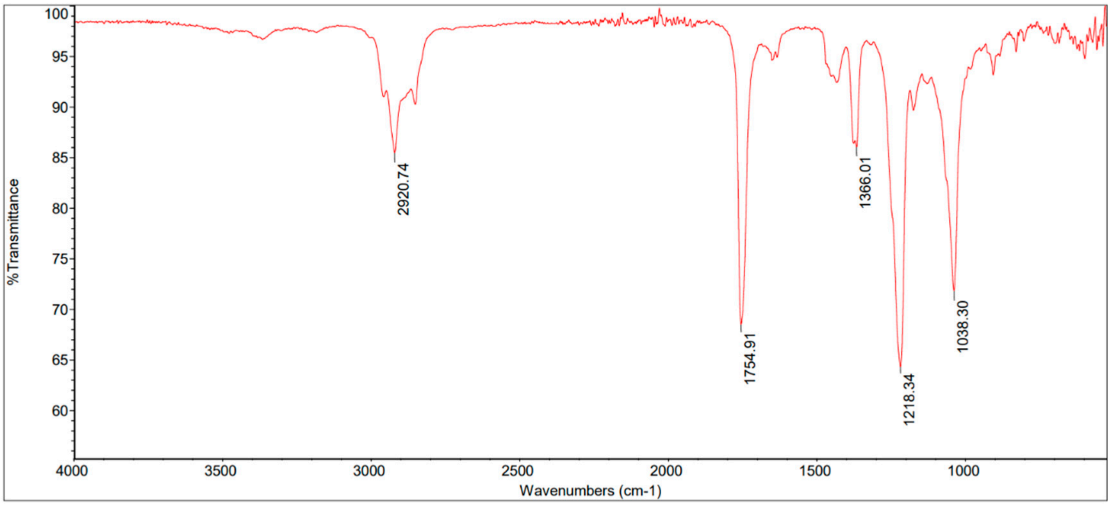

Fig. S86 Infrared spectrum of compound **7**.
